# Association of biological age with health outcomes and its modifiable factors

**DOI:** 10.1111/acel.13995

**Published:** 2023-09-18

**Authors:** Wei‐Shi Liu, Jia You, Yi‐Jun Ge, Bang‐Sheng Wu, Yi Zhang, Shi‐Dong Chen, Ya‐Ru Zhang, Shu‐Yi Huang, Ling‐Zhi Ma, Jian‐Feng Feng, Wei Cheng, Jin‐Tai Yu

**Affiliations:** ^1^ Department of Neurology and National Center for Neurological Diseases, Huashan Hospital, State Key Laboratory of Medical Neurobiology and MOE Frontiers Center for Brain Science Shanghai Medical College, Fudan University Shanghai China; ^2^ Institute of Science and Technology for Brain‐Inspired Intelligence, Fudan University Shanghai China; ^3^ Key Laboratory of Computational Neuroscience and Brain‐Inspired Intelligence (Fudan University), Ministry of Education Shanghai China; ^4^ Department of Neurology, Qingdao Municipal Hospital Qingdao University Qingdao China; ^5^ Department of Computer Science University of Warwick Coventry UK; ^6^ Fudan ISTBI—ZJNU Algorithm Centre for Brain‐Inspired Intelligence Zhejiang Normal University Jinhua China; ^7^ Shanghai Medical College and Zhongshan Hosptital Immunotherapy Technology Transfer Center Shanghai China

**Keywords:** Aging, biological age, disease, modifiable factor, mortality, unmodifiable factor

## Abstract

Identifying the clinical implications and modifiable and unmodifiable factors of aging requires the measurement of biological age (BA) and age gap. Leveraging the biomedical traits involved with physical measures, biochemical assays, genomic data, and cognitive functions from the healthy participants in the UK Biobank, we establish an integrative BA model consisting of multi‐dimensional indicators. Accelerated aging (age gap >3.2 years) at baseline is associated incident circulatory diseases, related chronic disorders, all‐cause, and cause‐specific mortality. We identify 35 modifiable factors for age gap (*p* < 4.81 × 10^−4^), where pulmonary functions, body mass, hand grip strength, basal metabolic rate, estimated glomerular filtration rate, and C‐reactive protein show the most significant associations. Genetic analyses replicate the possible associations between age gap and health‐related outcomes and further identify *CST3* as an essential gene for biological aging, which is highly expressed in the brain and is associated with immune and metabolic traits. Our study profiles the landscape of biological aging and provides insights into the preventive strategies and therapeutic targets for aging.

## Graphical abstract

The study included 59,316 healthy participants in the UK Biobank and considered 8276 phenotypes for developing biological age model. LightGBM algorithm was conducted to identify the most important predictors for biological age and build the model and the top 20 predictors were selected. We tested the longitudinal associations of age gap with 70 common health‐related outcomes, all‐cause mortality and cause‐specific mortality, and the genetic correlations of age gap with common health‐related outcomes. We identified 34 modifiable factors and 9 genomic risk loci for age gap and profiled the pleiotropy of rs3761280 in the UK Biobank.

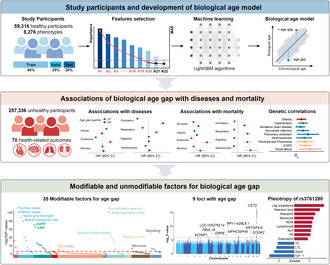

## 1 INTRODUCTION

Aging is characterized by the progressive loss of physiological functions and regeneration potential in multiple tissues and organs (Khan et al., 2017; López‐Otín, Blasco, et al., 2023). An unappreciated but important association between aging and multiple chronic disorders has been proposed for several decades, and aging is a shared mechanism and the major risk factor of various common diseases, including malignant neoplasms, atherosclerotic cardiovascular diseases, neurodegenerative disorders, and metabolic syndromes (Aunan et al., 2017; Hou et al., 2019; Liberale et al., 2022). Fortunately, recent studies suggested that aging was modifiable (Partridge et al., 2020) and have revealed some candidate behaviors or lifestyles that showed anti‐aging properties in animal models, like caloric restriction (Fontana & Partridge, 2015), physical activity (Neufer et al., 2015), and amino acid restriction (Levine et al., 2014). However, the modifiable factors for biological aging have not been systematically studied, and some other factors that may delay aging await discovery. In addition, genetics is also an essential approach for identifying the underlying mechanisms and pathways of biological aging, thus providing novel therapeutic targets and prevention opportunities for aging research (Melzer et al., 2020). As global aging population is still growing and the burden of age‐related diseases is increasing rapidly and has gradually become the most important causes of mortality and morbidity in elderly individuals (GBD 2017 Disease and Injury Incidence and Prevalence Collaborators, 2018; Guo et al., 2022), revealing the clinical implications and modifiable factors of aging and the underlying mechanisms is of great importance for reducing the socioeconomic and healthcare burden of age‐related diseases, thus promoting healthy aging.

As individuals age at different rates (Hamczyk et al., 2020), biological age (BA) was proposed as a term to estimate the rate and extent of biological aging and reflect the biological and physiological functions of individuals (Khan et al., 2017). Till now, multiple BA models have been proposed, including frailty index, Phenotypic Age, Klemera‐Doubal method Biological Age (KDM‐BA), and epigenetic PhenoAge (Cesari et al., 2014; Chen et al., 2022; Klemera & Doubal, 2006; Levine et al., 2018), and their associations with some common diseases were also reported. However, most BA measurements were generated with solely clinical indicators (i.e., Phenotypic Age or frailty index) or domain‐specific data (i.e., epigenomics, transcriptomics, or metabolomics aging clocks). In addition, aging is characterized by functional deterioration of multiple organs (Hernandez‐Segura et al., 2018). Therefore, a comprehensive BA measurement based on physical measures, biochemical assays, and omics data will thoroughly reflect the nature of biological aging and provide deeper insights into the association of biological aging with health outcomes, and its underlying determinants and therapeutic targets.

In addition, previous BA measurements were mainly generated by linear regression statistical modeling methods, which were limited by the curse of dimensionality and the complex correlation structure of the indicators (Rutledge et al., 2022). Machine learning (ML) can learn the patterns from multi‐dimensional data to build the model with the relevant features and make predictions on the new data (Baecker et al., 2021; Rutledge et al., 2022). Compared with the traditional linear regression models, ML makes inferences at individual levels, thus having great potential for clinical application (Baecker et al., 2021). Therefore, ML‐based algorithms are widely used to reduce the number of features in the development of predictive models (You et al., 2022, 2023), which may better capture the complexity of aging (Rutledge et al., 2022).

**FIGURE 1:**
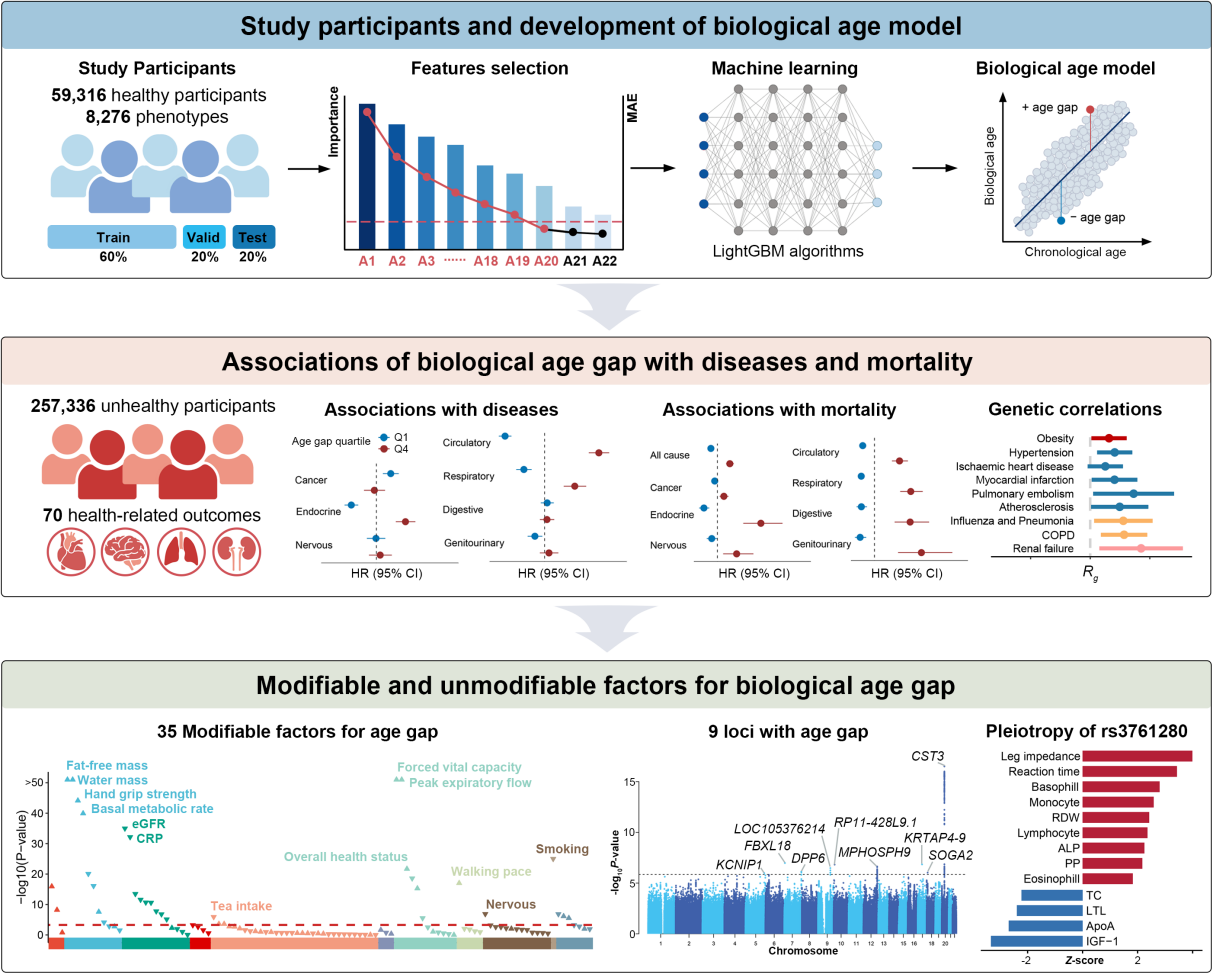
Graphical abstract of the study. Top part, the study participants and development of biological age model. The study included 59,316 healthy participants in the UK Biobank and considered 8276 phenotypes for developing biological age model. All healthy participants were further divided into training set (60%), validation set (20%), and testing set (20%). LightGBM algorithm was conducted to identify the most important predictors for biological age and build the model and the top 20 predictors were selected. Then the age gap, the difference between the estimated biological age and chronological age, was calculated within the participants. Middle part, the associations of age gap with diseases and mortality. We tested the longitudinal associations of age gap with 70 common health‐related outcomes, all‐cause mortality and cause‐specific mortality, and the genetic correlations of age gap with common health‐related outcomes. Bottom part, the modifiable and unmodifiable factors for age gap. We identified 34 modifiable factors and 9 genomic risk loci for age gap and profiled the pleiotropy of rs3761280 in the UK Biobank. ALP, alkaline phosphatase; ApoA, apolipoprotein A; CI, confidence interval; COPD, chronic obstructive pulmonary disease; CRP, C‐reactive protein; eGFR, estimated glomerular filtration rate; HR, hazard ratio; IGF‐1, insulin growth factor 1; LightGBM, Light Gradient Boosting Machine; LTL, leukocyte telomere length; PP, pulse pressure; RDW, red blood cell distribution width; TC, total cholesterol.

In the present study, we used the Light Gradient Boosting Machine (LightGBM) algorithm to develop an integrative BA model using multi‐dimensional data in the UK Biobank, a large longitudinal cohort of middle‐aged and older adults with a median follow‐up period of more than 10 years (Figure 1). We screened 59,316 healthy individuals throughout baseline and follow‐up, developed the BA model with physical measures, biochemical assays, cognitive functions, and genomics data, and then calculated the age gap, the deviation of BA from chronological age (CA). Next, we tested the longitudinal disease and mortality risk of age gap in unhealthy individuals. We further evaluated the genetic associations between age gap and the common health‐related outcomes. Then, we identified the modifiable factors for age gap and evaluated to what extent these factors delayed biological aging. Finally, we identified the genetic determinants of age gap and their underlying phenotypic mechanisms. Our work sheds light on the complexity of biological aging and its relevance to health‐related outcomes, modifiable traits, and genetic architecture, thus providing insights into the potential interventions for aging.

## 2 RESULTS

### 2.1 Predictors selection for biological age

**FIGURE 2:**
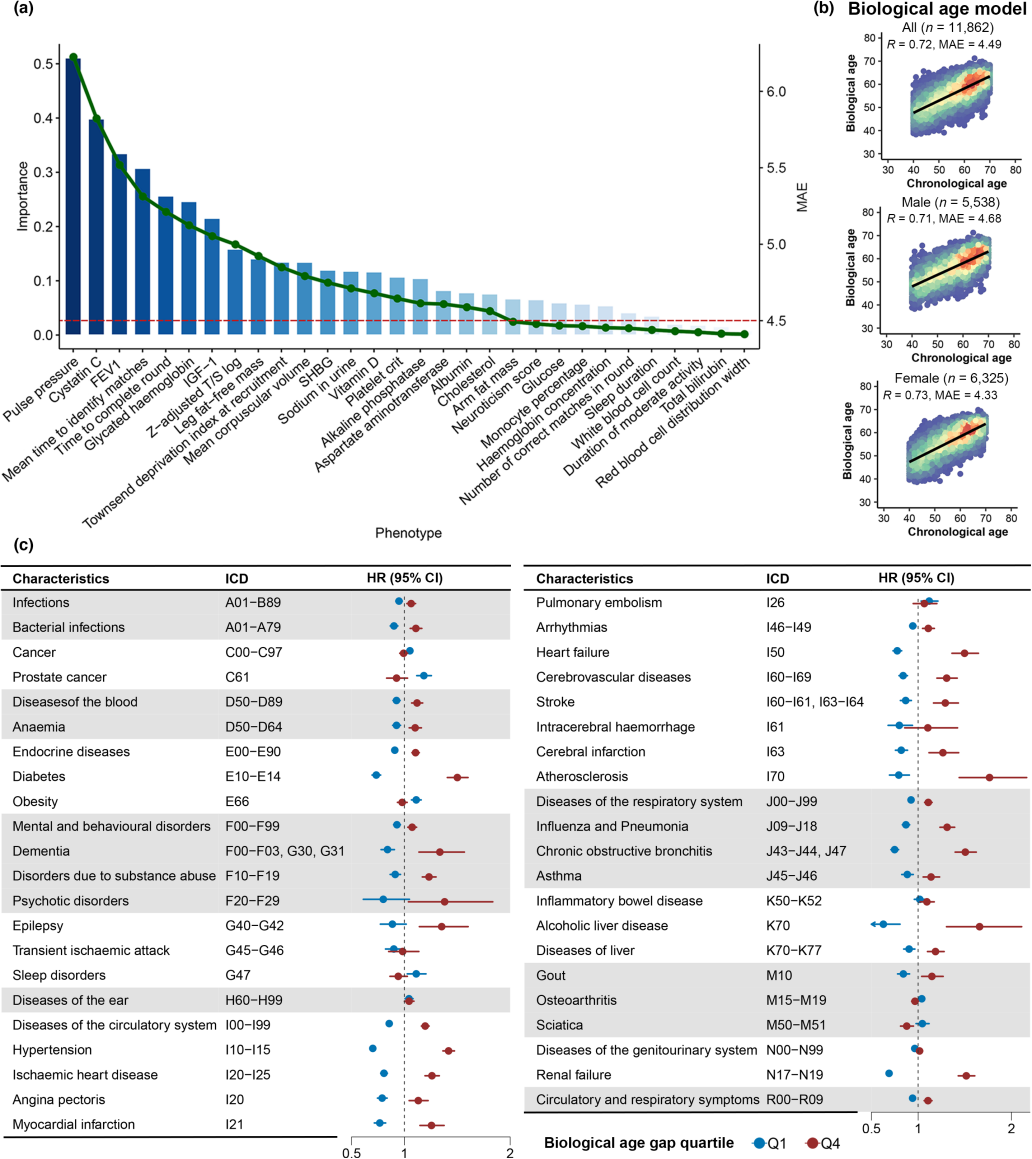
Predictor selection, performance, and implications of biological age. (a) The barplot showed the importance of phenotypes, which was the square root of the gain value generated from the LightGBM algorithm. The line chart showed the MAE when adding the phenotypes into the biological age model. (b) The scatter plot shows the distributions of biological age and chronological age of the participants. Each scatter indicated a single participant. The MAE and correlation coefficient of the model are shown in the left top part of the plot. (c) Associations of age gap with common health‐related outcomes. The forest plot shows the results of Cox proportional hazards regression analyses. Only the outcomes with nominally statistical significance (*p* < 0.05) are shown in the figure with the corresponding ICD‐10 codes. The Cox proportional model was adjusted for age at the recruitment, gender, ethnicity, education score, smoking status, alcohol drinking status, Townsend deprivation index, overall health rating, and number of medications/treatments taken. The second and the third quartiles of age gap (Q2 and Q3) are set as the reference, and other quartiles are marked with different colors. CI, confidence interval; HR, hazard ratio; ICD, international classification of diseases; IGF‐1, insulin growth factor; MAE, mean absolute error; SHBG, sex hormone binding globulin.

After screening, 59,316 healthy participants without any health‐related outcomes at baseline or during the follow‐up were included (Figure S1 and Table S1). The participants had a median age of 57 years and were predominantly women (31,530 [53.2%]) and of white ethnicity (55,609 [93.8%]). We divided all healthy participants into training sets (60%), testing sets (20%), and validation sets (20%). Of the 118 candidate predictors (Table S2), we first selected the top 50 predictors based on their importance to chronological age (CA). Some highly correlated predictors were identified, like forced vital capacity (FVC) and forced expiratory volume in 1‐second (FEV1), systolic blood pressure (SBP), diastolic blood pressure (DBP), and pulse pressure (PP). Therefore, hierarchical clustering was further conducted to eliminate multicollinearity (Figure S2). A total of 37 predictors remained and were ranked based on their importance to CA (Figure 2a). To determine the predictors for constructing the BA model, a sequential forward selection scheme was implemented. The performance of the BA model, which was determined by mean absolute error (MAE) shown on the right axis, was profiled by the line chart in Figure 2a (the full data can be found in Table S3). Finally, the top 20 phenotypes were selected as predictors for constructing the BA model. We profiled the BA model performance in the validation set (*n* = 11,862; Figure 2b). In all individuals, the MAE was 4.49. The model performance was relatively better among females, with an MAE of 4.33 than that of males (MAE = 4.68). In addition, lasso regression selected 48 features (Table S4), yielding an MAE of 4.90 in all individuals (Figure S3).

### 2.2 Longitudinal associations of age gap with diseases

To test the associations of age gap with the risk of common health‐related outcomes, we predicted BA in unhealthy participants with complete data (*n* = 257,336; Table S5) and calculated the age gap (BA minus CA). The participants were divided into four groups based on the age gap quartiles (Q1: age gap < −3.9; Q2: −3.9 ≤ age gap < −0.5; Q3: −0.5 ≤ age gap <3.2; Q4: age gap ≥3.2). Among the 70 common health‐related outcomes included in the analysis (see Table S6 for further details), during a median of 12.88 (Q1, 12.24; Q3, 13.53) years of follow‐up, the lowest and highest age gap quartiles were nominally associated with 43 outcomes (*p* < 0.05; Figure 2c and Table S7). After Bonferroni corrections (adjusted *p* = 0.05/70 = 7.14 × 10^−4^), we found that the highest age gap quartile was mainly associated with diseases of the circulatory system, including hypertension (HR [95% CI]: 1.42 [1.36–1.47], *p* = 2.00 × 10^−69^), ischemic heart diseases (1.26 [1.20–1.33], *p* = 1.46 × 10^−17^), myocardial infarction (1.26 [1.15–1.38], *p* = 7.41 × 10^−7^), arrhythmias (1.11 [1.05–1.17], *p* = 3.23 × 10^−4^), heart failure (1.50 [1.36–1.65], *p* = 4.37 × 10^−17^), atherosclerosis (1.77 [1.44–2.16], *p* = 4.12 × 10^−8^), stroke (1.29 [1.17–1.43], *p* = 1.04 × 10^−6^), and cerebral infarction (1.27 [1.12–1.43], *p* = 2.16 × 10^−4^). In addition to diseases of circulatory system, a higher age gap was also associated with some relevant chronic conditions, like anemia (1.10 [1.05–1.16], *p* = 2.68 × 10^−4^), diabetes (1.50 [1.41–1.60], *p* = 1.47 × 10^−34^), chronic obstructive bronchitis (1.51 [1.40–1.62], *p* = 8.39 × 10^−27^), and renal failure (1.52 [1.43–1.61], *p* = 6.02 × 10^−45^). Some associations between the highest age gap quartile and brain disorders were also observed, including dementia (1.34 [1.14–1.57], *p* = 3.95 × 10^−4^), disorders due to substance abuse (1.23 [1.17–1.30], *p* = 5.26 × 10^−15^), and epilepsy (1.35 [1.14–1.60], *p* = 4.70 × 10^−4^). However, we found that the lowest age gap quartile was associated with incident cancer, including any cancers (1.05 [1.02–1.08], *p* = 1.49 × 10^−4^) and prostate cancer (1.18 [1.12–1.26], *p* = 1.20 × 10^−8^).

### 2.3 Longitudinal associations of age gap with mortality

**FIGURE 3:**
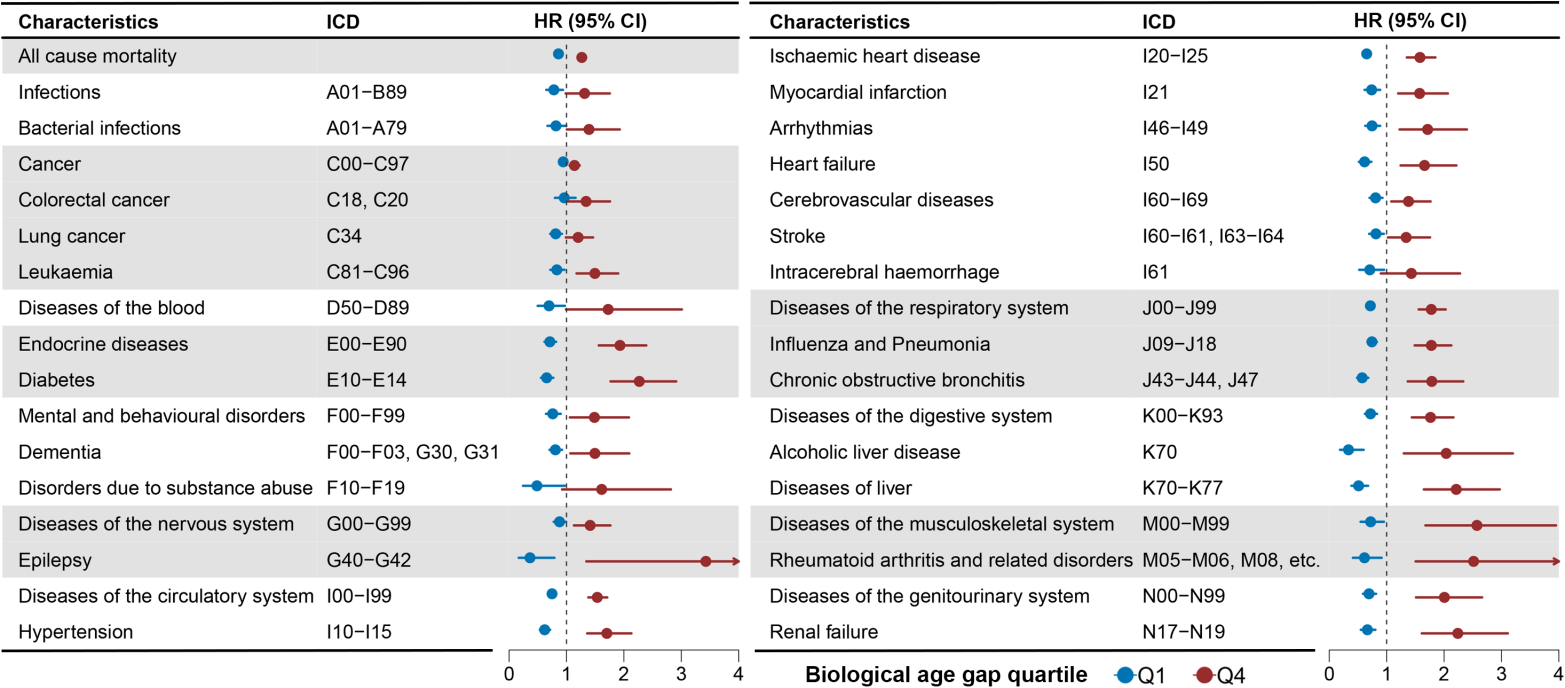
Associations of age gap with all‐cause and cause‐specific mortality. The forest plot shows the results of Cox proportional hazards regression analyses. Only the outcomes with nominally statistical significance (*p* < 0.05) were shown in the figure with the corresponding ICD‐10 codes. The Cox proportional model was adjusted for age at the recruitment, gender, ethnicity, education score, smoking status, alcohol drinking status, Townsend deprivation index, overall health rating, and number of medications/treatments taken. The lowest quantile of age gap (Q1) is set as the reference, and other quantiles are marked with different colors. CI, confidence interval; HR, hazard ratio; ICD, international classification of diseases.

We further tested the longitudinal associations of age gap with all‐cause and cause‐specific mortality among the unhealthy participants and found that the highest age gap quartile was associated with a 27% higher hazard for all‐cause mortality (HR [95% CI]: 1.27 [1.19–1.34], *p* = 4.29 × 10^−15^; Figure 3 and Table S8). Consistent with the results of incident diseases, the highest age gap quartile was significantly associated with the mortality due to diseases of the circulatory system (1.54 [1.38–1.71], *p* = 3.55 × 10^−15^), hypertension (1.70 [1.36–2.13], *p* = 3.30 × 10^−6^), and ischemic heart disease (1.58 [1.35–1.85], *p* = 1.07 × 10^−8^) after Bonferroni corrections (adjusted *p* = 0.05/70 = 7.14 × 10^−4^). In addition, we also observed positive associations between the highest age gap quartile and some chronic disorders (including diabetes, chronic obstructive bronchitis, disease of the liver, and renal failure). Overall, the longitudinal survival analysis of the age gap among the unhealthy participants suggested that the highest age gap was positively associated with the risk of incident diseases of the circulatory system, but negatively associated with the risk of incident cancers. While age gap was positively associated with all‐cause and cause‐specific mortality.

### 2.4 Modifiable factors for age gap

**FIGURE 4:**
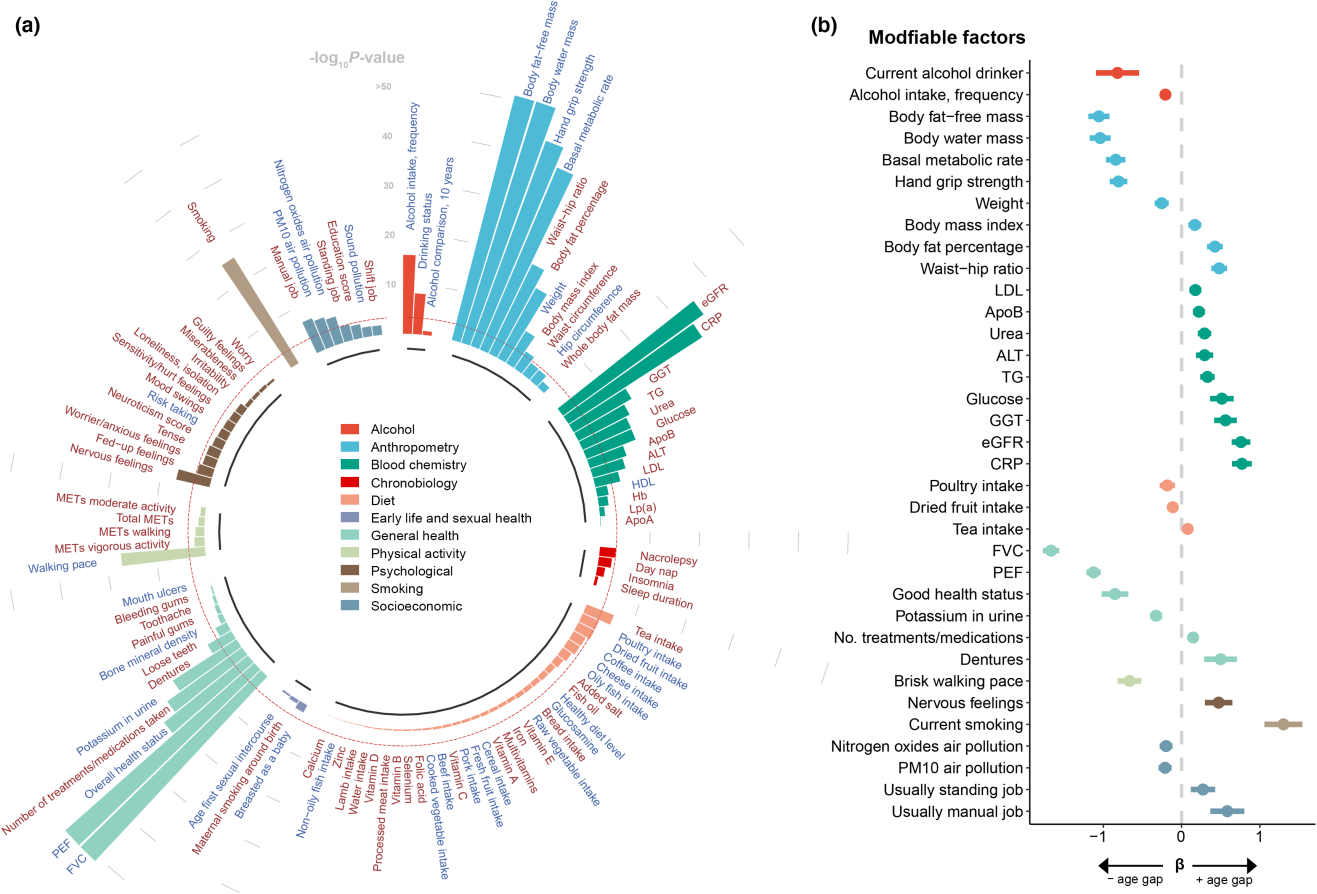
The associations of the modifiable factors and biological age gap in healthy participants. (a) The circular barplot shows the associations of the modifiable factors with biological age gap. The association with a *p*‐value of <1 × 10^−50^ was rounded to 1 × 10^−50^. The red dashed line indicates the threshold of adjusted *p*‐value (4.81 × 10^−4^). The modifiable factors were filled with different colors based on the categories. The red text indicates positive associations with age gap (*β* > 0), and the light blue text indicated negative association with age gap (*β* < 0). (b) The forest plot showed the estimated effects of the factors significantly associated with biological age gap. The *x*‐axis indicates the *β* coefficient of the traits. The bar indicated the 95% CI. Continuous traits were estimated for 1‐SD increase in the trait. Binary traits were estimated as yes versus no. Good health status was compared with fair or poor. A brisk walking pace was compared with a steady or slow pace. Usually standing and manual jobs were compared with sometimes, rarely, or never. ALT, alanine aminotransferase; ApoA, apolipoprotein A; ApoB, apolipoprotein B; CRP, C‐reactive protein; eGFR, estimated glomerular filtration rate; FVC, forced vital capacity; GGT, gamma glutamyltransferase; Hb, hemoglobin concentration; HDL, high‐density lipoprotein cholesterol; LDL, low‐density lipoprotein cholesterol; Lp(a), lipoprotein A; MET, metabolic equivalent task; PEF, peak expiratory flow; PM10, particulate matter with diameter less than or equal to 10 micrometers; TG, triglycerides.

Then we investigated the modifiable factors for biological age gap. We first screened 118 modifiable factors with low missing values in the UK Biobank. The factors included in the BA model were excluded, leaving 104 modifiable factors categorized into 11 clusters (Table S9). After Bonferroni correction, we identified 35 modifiable factors for age gap (*p* < 4.81 × 10^−4^, Figure 4a and Table S10). The most significant factors were pulmonary functions (FVC: *p* = 1.7 × 10^−210^; PEF: *p* = 8.01 × 10^−127^). Some modifiable factors associated with anthropometry were inversely associated with higher age gap, including body fat‐free mass (*p* = 7.23 × 10^−53^), body water mass (*p* = 6.18 × 10^−52^), hand grip strength (*p* = 7.45 × 10^−45^), and basal metabolic rate (*p* = 1.20 × 10^−40^). While higher body fat percentage was positively associated with a higher age gap (*p =* 7.90 × 10^−17^). In addition, several biochemical assays were also candidate modifiable factors for age gap, like eGFR (*p* = 1.02 × 10^−35^) and CRP (*p* = 6.88 × 10^−33^).

The effects of the significant modifiable factors on age gap were further profiled in Figure 4b. We found that the current alcohol drinkers had a 0.82‐year lower age gap. However, the current smokers had a 1.30‐year higher age gap. A higher body fat‐free mass per one‐standard deviation (1‐SD) was associated with a 1.06‐year decrease age gap, and the effect of body water mass on age gap was similar (*β* = −1.04 per 1‐SD increase). Regarding pulmonary functions, FVC and PEF were associated with a lower age gap, with 1.67 and 1.12 years per 1‐SD increase in FVC and PEF, respectively. Compared with the participants reporting fair or poor health ratings, those with good or excellent health status had a 0.85‐year lower age gap. The participants with a history of dentures had a 0.50 increase in age gap compared with those without a history of tooth diseases. In addition, the participants with nervous feelings had a 0.47‐year higher age gap than those without nervous feelings. We also found that compared with participants who sometimes or rarely did manual or physical jobs, those who usually or always did manual or physical jobs had a 0.58‐year higher age gap.

We also characterized the modifiable factors for age gap in men and women, respectively (Figures S4 and S5; Tables S11 and S12). After multiple comparisons, we identified 24 and 29 modifiable factors for men and women, respectively. Therefore, a total of 19 modifiable factors were shared by the entire and gender‐specific populations (Figure S6).

### 2.5 Genetic determinants for age gap

**FIGURE 5:**
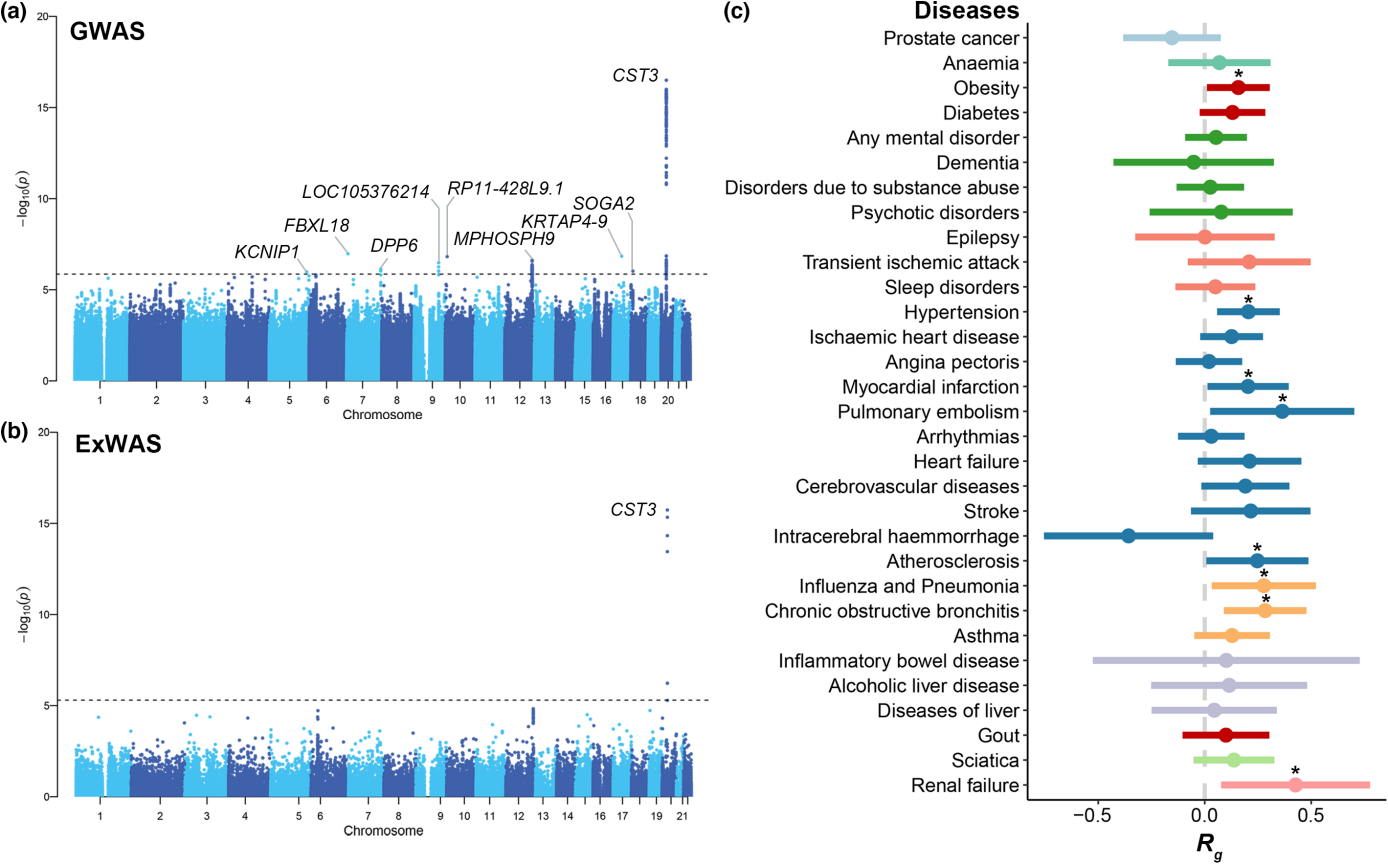
The genetic determinants and correlations of biological age gap. (a)The Manhattan plot shows the results of GWAS analysis of biological age gap in healthy participants. The *y*‐axis indicated the associations of the association of the locus with biological age gap. The loci with FDR < 0.05 were marked. (b) The Manhattan plot shows the results of ExWAS common variant analysis of biological age gap in healthy participants. The *y*‐axis indicated the associations of the association of the locus with biological age gap. The loci with FDR < 0.05 were marked. (c) The forest plot shows the results of LDSC analysis of biological age gap with the common health‐related outcomes that age gap was associated with the longitudinal survival analysis. The health‐related outcomes were filled with different colors based on the category. The outcomes that biological age gap nominally associated were marked with an asterisk. ExWAS, exomewide association study; GWAS, genome‐wide association study; LDSC, linkage disequilibrium score correlation.

To provide a comprehensive picture of age gap and to better understand the etiology of age‐related diseases, we next sought to identify the genetic determinants of biological age gap. To this end, we performed a genomewide association study (GWAS) analysis and exomewide association study (ExWAS) analysis of biological age gap in White British healthy participants in the UK Biobank (*n* = 9008; Figure 5a,b). The quantile‐quantile plot of GWAS and ExWAS is shown in Figures S7 and S8. In GWAS analysis, nine genomic risk loci significantly associated with age gap were identified after False Discovery Rate corrections (FDR <0.05, Figure 5a). The loci tagged by rs3761280 showed the most significant association (*p* = 3.17 × 10^−17^). See Table S13 for complete details of the loci. The single nucleotide polymorphism (SNP)‐based heritability for age gap was 20.9%. In ExWAS common variant analysis, only variants in *CST3* were significantly associated with age gap after False Discovery Rate corrections (FDR <0.05, Figure 5b). See Table S14 for complete details. In ExWAS gene‐based rare variant analysis, we failed to identify any genes significantly associated with age gap after multiple comparisons (Figure S9).

Next, we performed a linkage disequilibrium score correlation (LDSC) analysis of age gap with the common health‐related outcomes (see Table S15 for the details about the summary‐level GWAS data sets) identified in survival analyses (Figure 5c). The full information about LDSC analysis is shown in Table S16. Our analyses further supported the possible associations between biological age gap and diseases of circulatory system (hypertension: *R*_g_ = 0.21, *p* = 0.01; myocardial infarction: *R*_g_ = 0.20, *p* = 0.04; pulmonary embolism: *R*_g_ = 0.36, *p* = 0.04; atherosclerosis: *R*_g_ = 0.25, *p* = 0.04) and chronic diseases (obesity: *R*_g_ = 0.16, *p* = 0.04; chronic obstructive bronchitis: *R*_g_ = 0.28; *p* < 0.01; renal failure: *R*_g_ = 0.43, *p* = 0.02), though the genetic correlations did not survive correction for multiple comparisons.

FUnctional Mapping and Annotation (FUMA) was used to annotate the candidate single nucleotide polymorphisms (SNPs) in linkage disequilibrium with one of the independent significant SNPs via positional mapping (Table S17). Gene Ontology (GO) analysis suggested that the genes were mainly associated with the biological processes associated with translation (translational initiation and protein localization to plasma membrane; Figure S10). Kyoto Encyclopedia of Genes and Genomes (KEGG) analysis indicated that the genes were associated with the pathways associated with metabolism (cAMP signaling pathway and glycosphingolipid biosynthesis; Figure S10). See Table S18 for complete results of GO and KEGG analysis of the mapped genes.

### 2.6 Expression‐based and pleiotropy analysis of *CST3*

**FIGURE 6:**
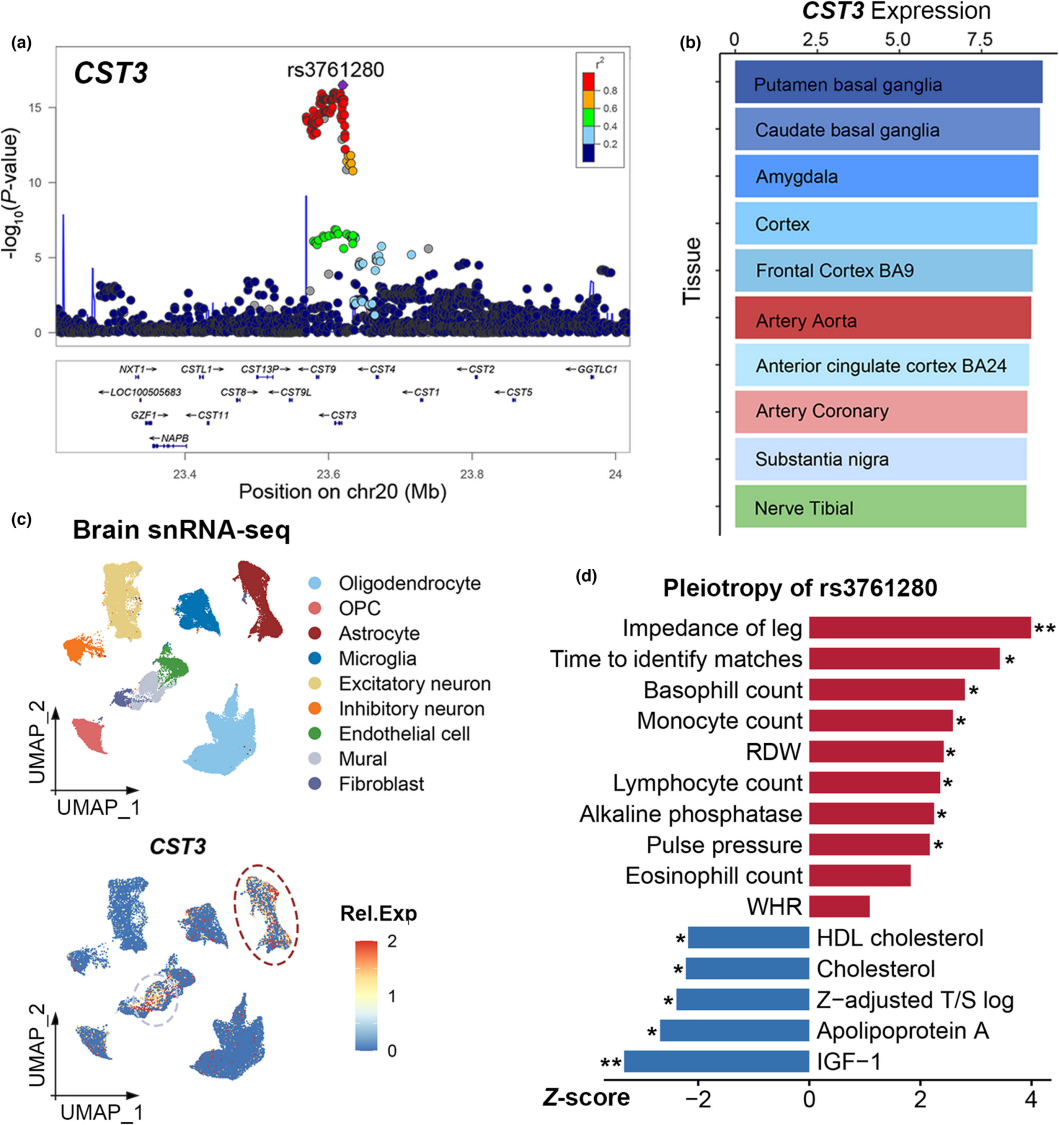
Expression analysis and pleiotropy of *CST3*. (a) Regional association plot of *CST3* in healthy White British participants. The region covering *CST3* ± 0.4 Mb was shown in the locus zoom plot. The SNP rs3761280 was highlighted and filed with purple color. The colors within the dots indicated the levels of linkage disequilibrium. (b) Tissue expression levels of *CST3*. The barplot shows the top 10 most *CST3*‐expressed tissues in GTEx v7. The *x*‐axis indicates the relative expression levels [log2(TPM + 1)]. (c) Cell type expression of *CST3*. The top section shows the UMAP of brain single‐nucleus transcriptomic data. Each dot represented an individual cell and was filled with different colors based on the result of clustering and annotation. The bottom section shows the expression level of *CST3* in the brain. (d) The pleiotropy of rs3761280 in the UK Biobank. The *x*‐axis indicated the *z‐*score of the association between rs3761280 and the characteristics. GTEx, genotype tissue expression; HDL, high‐density lipoprotein; OPC, oligodendrocyte progenitor cell; RDW, red blood cell distribution width; Rel.Exp, relative expression; snRNA‐seq, single‐nucleus RNA sequencing; TPM, transcripts per million; UMAP, uniform manifold approximation and projection; WHR, waist–hip ratio. *p < 0.05, **p < 0.01.

In GWAS analysis of age gap, rs3761280 located within gene *CST3* showed the most significant association (Figure 6a). We also conducted GWAS analysis of age gap in 9008 healthy and 201,795 unhealthy White British participants and also found a significant association of rs3761280 with age gap (Figure S11).

Leveraging Genotype Tissue Expression (GTEx) database, we found that *CST3* was highly expressed in the brain, particularly basal ganglia and cortex (Figure 6b). Then we analyzed single‐nucleus RNA sequencing (snRNA‐seq) data of the human brain, suggesting astrocytes and mural cells expressed relatively higher levels of *CST3* (Figure 6c). Colocalization analysis indicated that *CST3* expression levels in 10 tissues and age gap shared a causal variant with strong or suggestive evidence (Table S19).

Then we further investigated the pleiotropy of rs3761280 in the UK Biobank (Tables S20 and S21). We found that rs3761280 was nominally associated with 14 biomedical traits (Figure 6d). As expected, rs3761280 was significantly associated with cystatin C level (*Z*‐score = −121.157, *p* < 0.001; Table S21). Of note, rs3761280 showed an association with another biomarker of aging, leukocyte telomere length (*Z*‐score = −2.391, *p* = 0.017). Some associations with predictors in the BA model were also observed, like mean time to identify matches, alkaline phosphatase, PP, total cholesterol, and IGF‐1. In addition, some associations with inflammatory traits were observed, like the count of basophil, monocyte, and lymphocyte.

## 3 DISCUSSION

Our study has used large‐scale biobank data to develop an integrative BA model. Compared with the previous BA model generated with solely physical measures or biochemical assays, our BA model was a comprehensive model composed of multi‐dimensional indicators, including cognition, anthropometry, physical measures, biochemical markers, and genomic data. Next, we showed that the highest age gap was associated with the risk of multiple health‐related outcomes, especially circulatory diseases and related chronic disorders. Meanwhile, age gap was positively associated with all‐cause and cause‐specific mortality. We further identified 35 modifiable factors for age gap, highlighting body fat‐free mass and water mass, basal metabolic rate, pulmonary functions, and smoking status as candidate factors for delaying aging. Finally, genetic analyses including GWAS and ExWAS identified *CST3* as a novel gene associated with biological aging.

Machine learning, a data‐driven strategy, has been widely used to discover the essential indicators of BA, build BA models, and identify accelerated aging individuals (Gialluisi et al., 2022; Tian et al., 2023; Zhong et al., 2020). A recent study has trained brain age models in amyloid‐negative cognitively normal subjects (Millar et al., 2023), which supported our feature selection and model development in healthy participants. Consistent with most previous studies, our study revealed that blood pressure, pulmonary function, and glycated hemoglobin were essential indicators for BA (Chen et al., 2023; Gao et al., 2023). Pulse pressure, the difference between systolic and diastolic blood pressure, was identified as an essential predictor for aging in our feature selection, while most previous studies used systolic blood pressure to build BA (Chen et al., 2023; Gao et al., 2023; Tian et al., 2023). Pulse pressure is a marker reflecting arterial stiffness (Safar, 2018) and a recent cross‐sectional study has shown that PP was associated with accelerated epigenetic aging (Xiao et al., 2022). A cohort study with 32,833 participants reported that PP gradually increased as early as in the fourth decade and continued throughout the life course (Ji et al., 2020). In addition to PP, our study also identified some novel predictors for BA. For instance, we found that cognitive functions were candidate predictors for aging, which were usually included in calculating brain or cognitive age (Anatürk et al., 2021; Tian et al., 2023; Yu et al., 2022). The great importance of cognitive functions in BA further suggests the close correlation between the aging body and the brain. In addition, cystatin C, an index of renal function and a potential predictor of cardiovascular risk (Chen et al., 2023; Shlipak et al., 2005) was the second most essential predictor for BA. Likely, another renal function index, sodium in the urine, was also a predictor of BA, thus highlighting that renal dysfunction was closely associated with aging.

Accelerated aging was associated with various categories of diseases. Recent studies have shown that aging acceleration was associated with cardiovascular diseases, depression and anxiety, diabetes, and cognitive impairment (Chen et al., 2023; Forrester et al., 2021; Gao et al., 2023; Gialluisi et al., 2022). Our study identified some novel associations of biological age gap, thus expanding the clinical significance of biological aging. We first reported the positive association between age gap and some chronic disorders (anemia and gout) and infections (any or bacterial infections). Notably, some inverse associations of age gap were also observed, especially with cancers. This unexpected association could be explained by issues in methodology, like unmeasured or residual confounding factors. Moreover, some biological mechanisms may explain the negative association. Several aging hallmarks (telomere attrition and stem cell exhaustion) were antineoplastic (López‐Otín, Pietrocola, et al., 2023). Moreover, the malignant cells were hyperactive with a rapid cell cycle and increased energy consumption, while the senescent cells were hypoactive with an arrested cell cycle (Aunan et al., 2017). To further investigate the implications of biological aging, we tested its associations with mortality, suggesting that age gap was a promising predictor of all‐cause mortality. Intriguingly, we found that age gap was positively associated with cause‐specific mortality, including cancers and diseases of multiple systems. In addition to the putative association between age gap and cancer and cardiovascular diseases (Tian et al., 2023), our study also identified novel associations of age gap with mortality due to diseases of other systems, including hemopoietic, endocrine, digestive, and musculoskeletal systems. Overall, our BA model showed predictive value in all‐cause and cause‐specific mortality.

Anti‐aging strategies have been the focus in aging research. Previous studies have proved multiple modifiable factors for aging‐related disorders (Schloss et al., 2020; Yu et al., 2020). Therefore, revealing the modifiable factors for aging will not only help delay aging, but also help prevent the occurrence of age‐related disorders. However, the modifiable factors of biological aging have not been systematically characterized. A recent large‐scale study in the UK Biobank has revealed 71 traits associated with leukocyte telomere length, a potential biomarker of aging (Bountziouka et al., 2022). Our study found 35 candidate modifiable factors of age gap. Some factors, including smoking and pulmonary function, have been shown to be associated with epigenetic aging acceleration (Oblak et al., 2021). Pulmonary function starts to decline at age 25, with FEV1 and FVC decreasing by about 23–30 mL per year (Roman et al., 2016). Therefore, respiratory muscle training and respiratory rehabilitation serve as promising strategies for delaying biological aging (Roman et al., 2016; Skloot, 2017). Previous studies have shown that body fat‐free mass decreased significantly in the elderly population (Kyle et al., 2001), which showed predictive value in mortality (Genton et al., 2013). However, the association between body water mass and biological aging was rarely reported. The impaired renal function, physical, and cognitive disabilities in older adults would reduce body water mass and increase the risk of dehydration, which in turn leads to higher mortality and morbidity (Hooper et al., 2014). Nevertheless, higher fat percentage was associated with accelerated aging in our results. These analyses highlighted the importance of body composition in the aged population and indicated that caloric restriction, aerobic, and resistance exercises were promising interventions for biological aging (Batsis & Villareal, 2018). Factors reflecting renal functions were also significantly associated with aging, like eGFR, urea, and potassium in the urine, thus supporting the importance of the kidney in aging (O'Sullivan et al., 2017). Our study also found that nervous feelings were associated with biological aging. An observational study has suggested that mood disorders were associated with accelerated aging (Simon et al., 2006). However, the causal associations need to be clarified as accelerated aging also contributed to anxiety (Gao et al., 2023). In addition, some associations should be interpreted with caution in our study. For instance, our study showed that current alcohol drinking was negatively associated with biological aging. The unexpected association could be explained by the unadjusted effect measures or residual confounding (Kojima et al., 2018, 2019). In addition, most previous studies suggested that tea had protective effects on aging (Feng et al., 2021; Zhang et al., 2021). However, some studies have found that excess tea intake has an adverse impact on cognition and is associated with Alzheimer's disease (Hu et al., 2022; Sun et al., 2023).

In addition to the modifiable factors for aging, genetic factors were reported to contribute to approximately 50% of the total variance of aging (Gialluisi et al., 2022). In an Eastern Asian cohort, Lin revealed that loci in *GCKR*, *APOE*, and *FGF5* were associated with BA acceleration (Lin, 2022). Our study has leveraged both genotyping and exome sequencing data to reveal *CST3* as a candidate risk gene for biological aging. *CST3* encodes cystatin C, a marker of glomerular filtration rate and an essential inhibitor of cysteine proteases (Sukhova et al., 2005). Notably, cystatin C is also the second most essential predictor of BA, so the results of our genetic association analyses further strengthen the evidence that cystatin C may play a role in biological age from the genetic perspective. In a community‐based cohort study of older adults aged more than 65 years, cystatin C was regarded as a marker of unsuccessful aging (Sarnak et al., 2008). However, most studies failed to identify any genetic associations between cystatin C and aging or age‐related disorders (Loew et al., 2005; van der Laan et al., 2016). Therefore, our study provided evidence linking cystatin C with aging at genetic level. Previous studies have reported the associations of *CST3* with some age‐related disorders, like pulmonary fibrosis (Kim et al., 2018), age‐related macular degeneration (Butler et al., 2015), and Alzheimer's disease (Bertram et al., 2007). *CST3* was involved with arterial remodeling, neurogenesis, and neurotrophic function (Levy et al., 2006; Mi et al., 2007; Sukhova et al., 2005). *CST3* physically binds to the TGF‐β receptor and antagonizes the TGF‐β pathway, an essential pathway in senescent‐associated secretory phenotype (SASP) (Aging Biomarker Consortium et al., 2023; Sokol & Schiemann, 2004). Consistently, our results found that *CST3* was highly expressed in astrocytes, the essential supportive cells within brain (Endo et al., 2022). In addition, a cohort study demonstrated that cystatin C was a candidate marker for inflammation (Koenig et al., 2005). Our study further supported the hypothesis as the *CST3* locus, rs3761280, was probably associated with immune cell indices. Moreover, rs3761280 was also associated with the traits associated with metabolism (IGF‐1 and blood lipids). Therefore, our analyses highlighted the putative role of inflammatory and metabolic pathways in aging pathogenesis.

Our study had several strengths and implications. The large‐scale and multi‐dimensional phenotypes available in the UK Biobank have enabled us to develop an integrative BA model and systematically analyze the modifiable and unmodifiable factors for biological aging. We demonstrated that biological aging was modifiable by multiple factors. Given the associations between biological aging and multiple health‐related outcomes, these factors had the potential to delay aging and thus reduce the risk of age‐related disorders. However, several limitations in our study should be noticed. First, the development of BA model was restricted to the healthy participants from a subpopulation of the large cohort. Second, the analysis of the modifiable factors for age gap was cross‐sectional, thus caution was necessary about making causal inferences. Third, the participants in the UK Biobank were mostly White British population and tended to be healthier and wealthier (Keyes & Westreich, 2019). The generalizability of our BA model needs external validation in different ethnicities or cohorts.

In conclusion, our study has developed an integrative BA model consisting of multi‐dimensional indicators, and the biological age gap showed predictive value in incident disease, all‐cause, and cause‐specific mortality. Our study further systematically revealed the modifiable and unmodifiable factors for biological aging, which provided promising candidates for further experimental and clinical research.

## 4 MATERIALS AND METHODS

### 4.1 Study participants

The present study analyzed the data from the UK Biobank, which consisted of 502,409 participants aged 37–73 years at the time of the first assessment (from 2006 to 2010) (Bycroft et al., 2018). The baseline data including biological sample (blood and urine) assays, physical measurements, socioeconomic characteristics, and genotyping data were collected at baseline from 22 assessment centers across the UK (Bycroft et al., 2018). The UK Biobank has approval from the North West Multi‐center Research Ethics Committee and all participants in the UK Biobank have provided written informed consent.

### 4.2 Development of biological age and age gap

To develop BA, we first screened the healthy participants in the UK Biobank. Those who had any health‐related outcomes at baseline or would develop any health‐related outcomes during the follow‐up were excluded. In addition, the participants who withdrew from the UK Biobank were also excluded, leaving 59,316 healthy participants. The health‐related outcomes were defined as the first occurrences (Category 1712) in the UK Biobank, which included 1165 health‐related outcomes categorized into 16 categories.

The study took all phenotypes (*n* = 8276) available in the UK Biobank into consideration at first and then took several steps to filter the phenotypes to develop a biological age model. In the first step, the phenotypes with high missing values (> 30% in all participants from the UK Biobank, *n* = 7482) were excluded. Then in the second step, we further excluded the categorical phenotypes (*n* = 448) and nonclinically relevant phenotypes (*n* = 303). In addition, the variables were averaged if tested for both the right and left sides of the body, like hand grip strength, the fat mass of the legs or arms. The variables were also averaged if tested more than once at baseline, like diastolic and systolic blood pressure, pulse rate, FVC, and FEV1. We also included four derived variables, including pulse pressure (the deviation of systolic and diastolic blood pressure; PP), mean arterial pressure (the sum of diastolic pressure and one‐third of pulse pressure; MAP), waist–hip ratio (the ratio of waist and hip circumference; WHR), and FEV1/FVC. Overall, there were 118 phenotypes considered for developing BA (see Supplements for further details). Then the data was imputed using the multiple imputations by chained equations approach, with five imputed data sets and 10 iterations (White et al., 2011).

Then in the third step, we used LightGBM algorithm to filter the candidate phenotypic features. Briefly, the 118 features were ranked based on their information gains, an inherent approach within tree‐based machine learning algorithms, which can be considered as the predictive contributions to estimating chronological age. We selected the top 50 phenotypes and then performed hierarchical clustering on the Spearman rank‐order correlations to alleviate the multicollinearity issue, in such highly correlated features were clustered together and we kept only a single predictor within clusters under an arbitrary adopted threshold of 0.7. After the removal of correlated predictors, all phenotypic features were re‐ranked based on a newly developed LightGBM classifier. Next, in the fourth step, consecutive classifiers were developed with sequentially added predictors based on the updated predictor importance ranking orders. The stopping point was reached when the difference between the previous and the present predictor was less than 0.02 (Table S3). In addition, it was noted that no significant improvement in model performance could be observed when additional predictors come into the model. Overall, the top 20 predictors were identified for the development of BA via the LightGBM algorithm. The employed LightGBM algorithm works by starting from a weak base learner, usually a decision tree model, and sequentially training each new learner to correct the errors from the previously trained ones. In such a manner, the predictions can be added up to produce a strong overall final predictive model. The hyperparameter tuning was performed by an exhaustive selection from 500 candidate sets of parameters and finally the optimal set based on the performance measurement of MAE. The hyperparameters to develop the BA model were as follows: *learning_rate* = 0.05; *max_depth* = 5; *n_estimators* = 800; *num_leaves* = 31; *subsample* = 0.8; *colsample_bytree* = 0.8. The supporting information about the parameters can be found on the website of LightGBM documentation (https://lightgbm.readthedocs.io/en/latest/Parameters‐Tuning.html). The LightGBM algorithm was implemented by the R package lightgbm version 3.3.3. under the R software version 4.2.0. The model was developed and validated using a five‐fold cross‐validation strategy that the validation set (one‐fold of data) was kept untouched and merely used for evaluation purposes, while the hyperparameters tuning and post‐calibration were performed within inner‐looped cross‐validation within the training sets (four‐fold of data). For comparison purposes, we employed standard regression, lasso regression, to calculate BA with the same phenotypes. Lasso regression was conducted by the R package glmnet and performed 10‐fold cross‐validation.

### 4.3 Health‐related outcomes characterization

The diagnoses and medical conditions of the participants were obtained through hospital inpatient record data, primary care data, death register data, and self‐reported data from the UK National Health Services. The diseases were recorded by the distinct International Classification of Disease (ICD)‐10 system. A total of 78 common health‐related outcomes reported in a previous study were investigated in our study (Kivimäki et al., 2021, 2022), and the unhealthy participants with full data to predict BA were included (*n* = 257,336). We excluded the diseases with less than 200 cases among the unhealthy participants and the miscellaneous outcomes (i.e., self‐harm and road accidents), leaving 70 common health‐related outcomes. Regarding cause‐specific mortality, those with less than 50 cases were excluded from the analysis. The details of the health‐related outcomes can be found in Table S4. Regarding mortality, the UK Biobank receives death notifications, including age at death and primary causes of death determined by ICD‐10, by linkage to national death registries. The cause‐specific mortality (including both primary and contributory causes of mortality) was defined using the following codes based on the ICD‐10 system. For incident disease, the end of follow‐up was defined as the date of first diagnosis of the disease, death, loss to follow up, or end of hospital inpatient data collection on December 31, 2021. For all‐cause and cause‐specific mortality, the end of follow‐up was defined as the date of death, loss to follow up, or end of hospital inpatient data collection on December 31, 2021.

### 4.4 Modifiable factors

We collected 118 potentially modifiable factors in the UK Biobank data. And we have confirmed that the 118 modifiable factors had a low proportion (<30%) of missing values. After excluding the factors included in the BA model, the modifiable factors could be subclassified into 11 categories: (1) alcohol (e.g., alcohol intake frequency), (2) anthropometry (e.g., body mass index), (3) blood chemistry (e.g., glucose, hemoglobin), (4) chronobiology (e.g., sleep duration), (5) diet (e.g., tea intake), (6) early life and sexual health (e.g., age first sexual intercourse), (7) general health (e.g., FEV1), (8) physical activity (e.g., walking pace), (9) psychological (e.g., nervous feelings), (10) smoking (smoking status), and (11) socioeconomic (e.g., education). The information about the field ID and processing of the phenotypes is presented in Table S7. Data of the modifiable factors was imputed using the multiple imputation by chained equations approach, with five imputed data sets and 10 iterations.

### 4.5 Genomewide association analysis

PLINK software (version 2.0) was used to perform GWAS analysis (Purcell et al., 2007). The quality control of the genotype sequencing data was conducted as follows: the individuals with missing genotype rate >0.05, mismatch between self‐reported and genetic sex, putative sex chromosome aneuploidy, heterozygosity rate outliers, putative third‐degree relatives >10 were excluded. The variants with call rate <0.95, minor allele frequency (MAF) < 0.005, Hardy–Weinberg *p*‐value <10^−6^, or imputation quality score <0.5 were excluded. Only the individuals of White British ancestry were included in the GWAS analysis. Age at recruitment, gender, genotype array, and the top 10 principal components (PCs) were used as the covariates. The healthy participants GWAS was used as the main analysis, with about 9008 participants. To test the robustness of the results, we also performed GWAS of both healthy and unhealthy participants and the final sample size of it was 210,801.

### 4.6 Exomewide association analysis

Regarding common variants (at least 10 total carriers with age gap characteristics of White British ancestry), the associations with age gap were analyzed using a linear regression model using PLINK 2.0 (Purcell et al., 2007). Age at recruitment, gender, and the top 10 PCs were covariates. Regarding rare variants, the SKAT‐0 test by SAIGE‐GENE+ strategy was conducted to analyze the rare‐variant gene associations (Zhou et al., 2022).

SAIGE‐GENE+ method is used for region‐based association analysis that is capable of processing large‐scale samples and can collapse the ultra‐rare variants (which are defined as minor allele carrier [MAC] ≤ 10) to a single marker and then test the collapsed variant together with all other variants with MAC > 10, which reduces the data sparsity due to the effects of ultra‐rare variants (Zhou et al., 2020, 2022). Three different maximum minor allele frequency (MAF) cutoffs (1%, 0.1%, and 0.05%) and three different variant annotations (loss‐of‐function, missense, and loss‐of‐function and/or missense), followed by aggregating multiple SKAT‐O tests using the Cauchy combination or minimum *p*‐value for each gene or region (Li et al., 2020; Liu & Xie, 2020). SnpEff Version 5.1 was used to annotate and classify the variants of all samples (Cingolani et al., 2012). The LOF variants include the variants annotated as frameshift, splicing donor, splicing acceptor, and stop gain. The missense variants include the variants predicted as deleteriousness in Sorting Intolerant From Tolerant (SIFT) (Vaser et al., 2016), Polymorphism Phenotyping v2 (PolyPhen2) HDIV (Adzhubei et al., 2013) and PolyPhen2 HVAR (Adzhubei et al., 2013); likelihood ratio test (LRT) (Chun & Fay, 2009); and MutationTaster (Schwarz et al., 2010), and are further collapsed for each gene. The ExWAS model was adjusted by age at the recruitment, gender, and first 10 PCs.

### 4.7 Heritability estimation

The heritability of the SNP was estimated by the genome‐based restricted maximum likelihood (GREML) method implemented in the genomewide complex trait analysis (GCTA) software v1.93.2 (Yang et al., 2011). Age at recruitment, gender, genotype array, and the top 10 principal components (PCs) were used as the covariates.

### 4.8 Genomic risk loci characterization and gene mapping

FUMA was used to identify the genomic risk loci and map the genes (Watanabe et al., 2017). The SNPs with FDR <0.05 and linkage disequilibrium (LD) *r* ^2^ < 0.6 with any other SNPs were considered independent significant SNPs. The SNPs with LD *r* ^2^ < 0.1 with any other SNPs were considered genomic risk loci. The 1000G Phase 3 European project was used as the reference. Positional mapping (based on the physical distance within 10 kb of the SNP) method was used to conduct gene mapping analysis of the genomic risk loci.

### 4.9 Functional enrichment of genes

The functional enrichment of the genes was conducted using the R package clusterProfiler (Wu et al., 2021). The mapped genes were analyzed for the enrichment of biological processes, molecular function, and cellular component from Gene Ontology (GO) (Gene Ontology Consortium, 2015) and pathway from Kyoto Encyclopedia of Genes and Genomes (KEGG) (Kanehisa & Goto, 2000). The default parameters of a minimum of 5 and a maximum of 2000 genes per category were used. The terms or the pathways with FDR less than 0.05 were considered as enrichment.

### 4.10 Genetic correlation analysis

Genetic correlation analysis of biological age gap with diseases was conducted by LDSC v.1.0.1 (Bulik‐Sullivan et al., 2015). The summary‐level GWAS data of the diseases were obtained from FinnGen (Kurki et al., 2023). The precomputed European LD scores from the 1000 Genomes Project phase 3 in the LDSC package were used and the LDSC analysis was restricted to Hapmap3 SNPs.

### 4.11 Expression‐based analysis

Regarding the expression level of the genes in different tissues or organs, bulk RNA sequencing data from GTEx were obtained (GTEx Consortium, 2015).

To further test the cell type expression levels, a large human brain single‐nucleus RNA sequencing data set by Garcia et al. (2022) was obtained, including 61,862 individual cells including neurons, glia, and cerebrovascular cells. The R package Seurat was used for the main analysis and visualization (Butler et al., 2018). The annotation of the cell type was conducted using the metadata file provided by the authors (Garcia et al., 2022).

Regarding colocalization analysis, we obtained eQTL data from 46 different organs or tissues from GTEx v7 (GTEx Consortium, 2015). The R package coloc was used for colocalization analysis (Giambartolomei et al., 2014). Colocalization analysis utilized approximate the Bayes factor to generate posterior probabilities (PP). Default parameters (*p*1 = 10^−4^, *p*2 = 10^−4^, *p*12 = 10^−5^) were used. Strong evidence of colocalization was defined as PPH3 + PPH4 ≥ 0.99 and PPH4/PPH3 ≥ 5. Suggestive evidence of colocalization was defined as PPH3 + PPH4 ≥ 0.90 and PPH4/PPH3 ≥ 3 (Codd et al., 2021; Jin et al., 2016).

### 4.12 Statistical analysis

The Cox proportional hazard regression model was used to test the longitudinal associations of biological age gap with the risks of common health‐related outcomes, all‐cause, and cause‐specific mortality. Regarding the association with incident diseases, the participants with the specific diagnosis before or at the time of recruitment were excluded from the models. Age at recruitment, gender, ethnicity, education score, smoking status, alcohol drinking status, TDI, overall health rating, and number of medications/treatments taken were used as covariates. Proportional hazards of the associations were tested using Schoenfeld's residuals. The second and the third biological age gap quartiles (Q2 and Q3) were set as the reference. Bonferroni correction was performed for multiple comparisons.

A multivariable linear regression model was used to test the modifiable factors for biological age gap, which was set as the response variable. For continuous traits, the data was *z*‐normalized before analysis. Age at recruitment and gender were used as the covariates. In the analyses of continuous traits with age gap, the outlier values (defined as the values 1.5 times interquantile range (IQR) lower than the lower quartile or 1.5 times IQR higher than the upper quartile) were winsorized at the 5% and 95% percentile values. Bonferroni correction was used for multiple comparisons.

Regarding the pleiotropic analysis of rs3761280, a multivariable linear regression model was used. A total of 94 biological traits were considered and analyzed with an analysis of variance (ANOVA) test. Only the traits with statistical significance (*p* < 0.05) among the rs3761280 genotypes were further analyzed. Age at recruitment and gender were adjusted in the model.

R software version 4.2.0 was used to conduct data cleaning, analyses, and visualization. A two‐sided *p*‐value of <0.05 was considered statistically significant.

## CODE AVAILABILITY

This study used open source software and codes, specifically R (https://www.r‐project.org/), lightgbm (https://github.com/Microsoft/LightGBM), PLINK (https://www.cog‐genomics.org/plink/), GCTA (http://cnsgenomics.com/software/gcta/), FUMA (https://fuma.ctglab.n/l), MAGMA (https://ctg.cncr.nl/software/magma), SAIGE‐GENE+ (https://saigegit.github.io/SAIGE‐doc/), LDSC (https://github.com/bulik/ldsc/), clusterProfiler (https://github.com/YuLab‐SMU/clusterProfiler), Seurat (https://satijalab.org/seurat/index.html), and coloc (https://github.com/chr1swallace/coloc).

## AUTHOR CONTRIBUTIONS

Jian‐Feng Feng, Wei Cheng, and Jin‐Tai Yu designed the study. Wei‐Shi Liu and Jia You conducted the main analyses and drafted the manuscript. Yi‐Jun Ge and Bang‐Sheng Wu conducted the genetic analyses. Shi‐Dong Chen and Ya‐Ru Zhang contributed to the interpretation of the results. Yi Zhang, Shu‐Yi Huang, and Ling‐Zhi Ma contributed to data collection. Jian‐Feng Feng, Wei Cheng, and Jin‐Tai Yu critically revised the manuscript. All authors reviewed and approved the final version of the manuscript and all authors had full access to the data in the study and accept responsibility to submit for publication.

## FUNDING INFORMATION

This study was supported by grants from the Science and Technology Innovation 2030 Major Projects (2022ZD0211600), National Natural Science Foundation of China (92249305, 82271471, 82071201, 82071997), Shanghai Municipal Science and Technology Major Project (2018SHZDZX01), Research Start‐up Fund of Huashan Hospital (2022QD002), Excellence 2025 Talent Cultivation Program at Fudan University (3030277001), Shanghai Talent Development Funding for The Project (2019074), Shanghai Rising‐Star Program (21QA1408700), 111 Project (B18015), and ZHANGJIANG LAB, Tianqiao and Chrissy Chen Institute, the State Key Laboratory of Neurobiology and Frontiers Center for Brain Science of Ministry of Education, Shanghai Center for Brain Science and Brain‐Inspired Technology, Fudan University.

## CONFLICT OF INTEREST STATEMENT

The authors declared no conflicts of interest in the article.

## Supporting information


Data S1.

## Data Availability

The main data, including individual‐level phenotype and genetic data used in this study, were accessed from the UK Biobank under application number 19542. The summary‐level GWAS data used in this study were acquired from the FinnGen study, which was available from the website: https://www.finngen.fi/fi. The bulk RNA‐seq and eQTL data used in this study were acquired from the GTEx consortium (https://www.gtexportal.org/home/index.html). The snRNA‐seq used in this study was acquired from the GEO database (https://www.ncbi.nlm.nih.gov/geo/) with accession number: GSE173731.
