# Supplementary material for: Association of biological age with health outcomes and its modifiable factors
Source: Aging Cell. 2023 Sep 18;22(12):e13995. doi: 10.1111/acel.13995 (PMC10726867; doi:10.1111/acel.13995)
Supplement: Supplementary file 1 — Data S1. [file ACEL-22-e13995-s001.docx]

**Content of Supplementary Materials**

**Figure S1**. Flow chart of the development of biological age model. 2

**Figure S2**. Correlations heatmap and clustering dendrogram of the top 50 candidate predictors of BA model. 3

**Figure S3**. The associations of modifiable factors and age gap in healthy male participants. 5

**Figure S4**. The associations of modifiable factors and age gap in healthy male participants. 6

**Figure S5**. The associations of modifiable factors and age gap in healthy female participants. 7

**Figure S6**. Shared modifiable factors for age gap. 8

**Figure S7**. Q-Q plot for GWAS analysis of age gap in healthy participants. 9

**Figure S8**. Q-Q plot for ExWAS analysis of age gap in healthy participants. 10

**Figure S9**. ExWAS gene-based rare variant analysis of age gap. 11

**Figure S10**. GO and KEGG analysis of the mapped genes associated with age gap. 12

**Figure S11**. Regional association plot of rs3761280 in healthy and unhealthy White British participants. 13

**Table S1**. Demographic characteristics of the healthy participants in the UK Biobank. 14

**Table S2**. Details about the phenotypes considered in constructing BA model. 15

**Table S3**. The biological age model performance at different numbers of predictors 20

**Table S4.** Feature selection using lasso regression. 21

**Table S5.** Demographic characteristics of the unhealthy participants in the UK Biobank. 23

**Table S6**. The common health-related outcomes included in the longitudinal analysis. 24

**Table S7**. Longitudinal associations of age gap with the risk of diseases. 26

**Table S8**. Longitudinal associations of age gap with the risk of mortality. 29

**Table S9**. Details of the modifiable factors. 31

**Table S10**. Results of multivariable regression analysis between modifiable factors and age gap. 36

**Table S11**. Results of multivariable regression analysis between modifiable factors and age gap in health male participants. 39

**Table S12**. Results of multivariable regression analysis between modifiable factors and age gap in healthy female participants. 42

**Table S13**. Genomic risk loci for age gap. 45

**Table S14**. The common variants associated with age gap after FDR corrections. 46

**Table S15**. GWAS summary-level data used in LDSC analysis. 47

**Table S16**. Results of LDSC analysis of age gap with health-related outcomes. 49

**Table S17**. Results of gene mapping analysis. 50

**Table S18**. Results of GO and KEGG analyses. 51

**Table S19**. Results of colocalization analysis of *CST3* tissue expression with age gap. 57

**Table S20**. Biological characteristics and rs3761280 genotypes. 59

**Table S21**. Results of linear regression analysis of rs3761280 with the biological traits. 63

**Figure S1. Flow chart of the development of biological age model.**


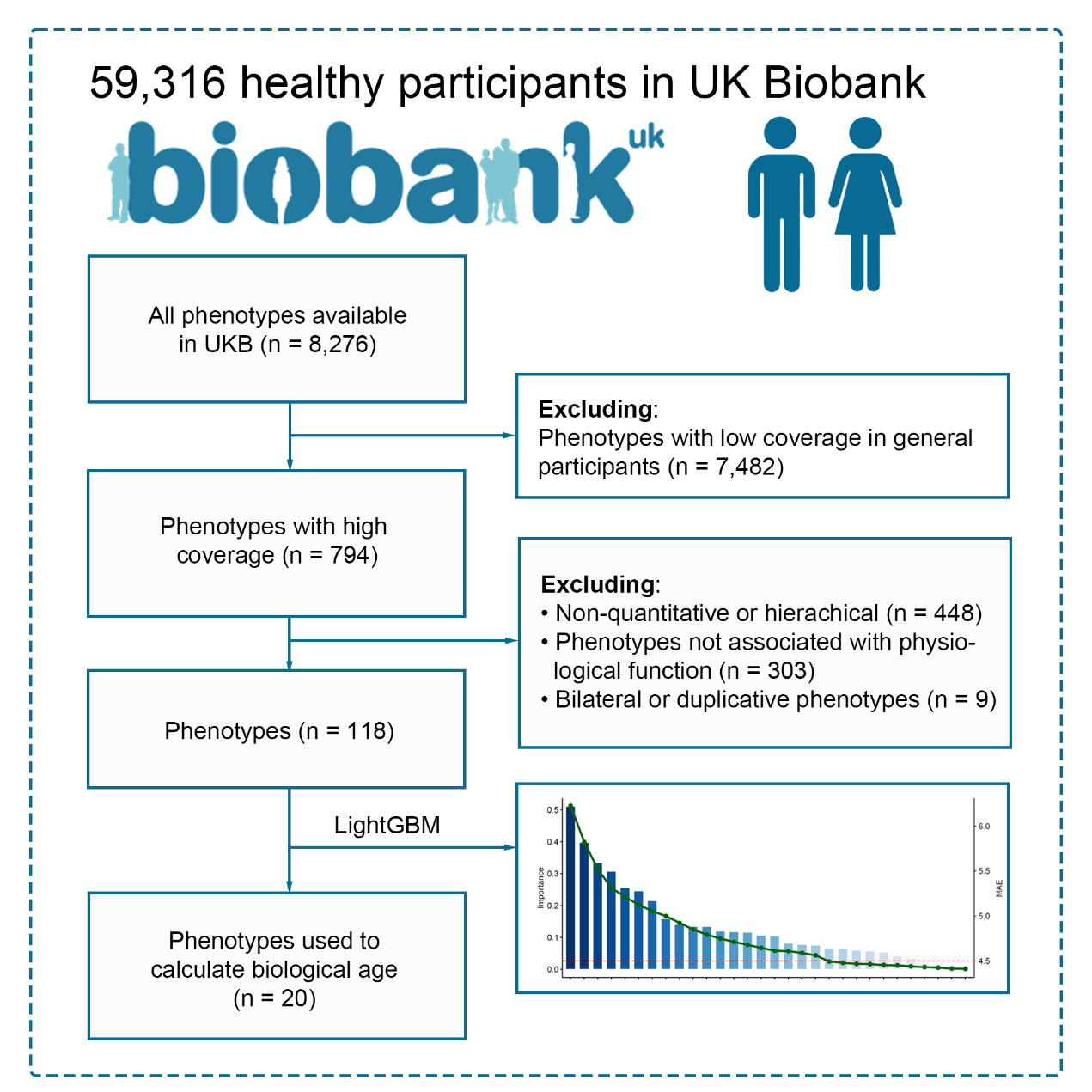


A total of 59,316 healthy participants in the UK Biobank were included in biological age model development. There were 8,276 phenotypes available in the UK Biobank and those with low coverage (less than 70% of all participants) were excluded, leaving 794 phenotypes. Then, we excluded the categorical phenotypes (*n* = 448) and the phenotypes not associated with the physiological function of the human body (i.e., height, fasting time, and duration of questionnaire; *n* = 303). Moreover, the phenotypes with bilateral assessment (i.e., hand grip strength and leg/arm mass) were averaged. And regarding the phenotypes with repeated measurements (i.e., parameters of pulmonary function and blood pressure), the average values were taken. And 118 phenotypes were included to develop biological age via Light GBM algorithm. The top 20 phenotypes were selected, where the MAE reached a value below 4.5.

LightGBM: Light Gradient Boosting Machine; MAE: mean absolute error; UKB: UK Biobank.

**Figure S2. Correlations heatmap and clustering dendrogram of the top 50 candidate predictors of biological age model.**


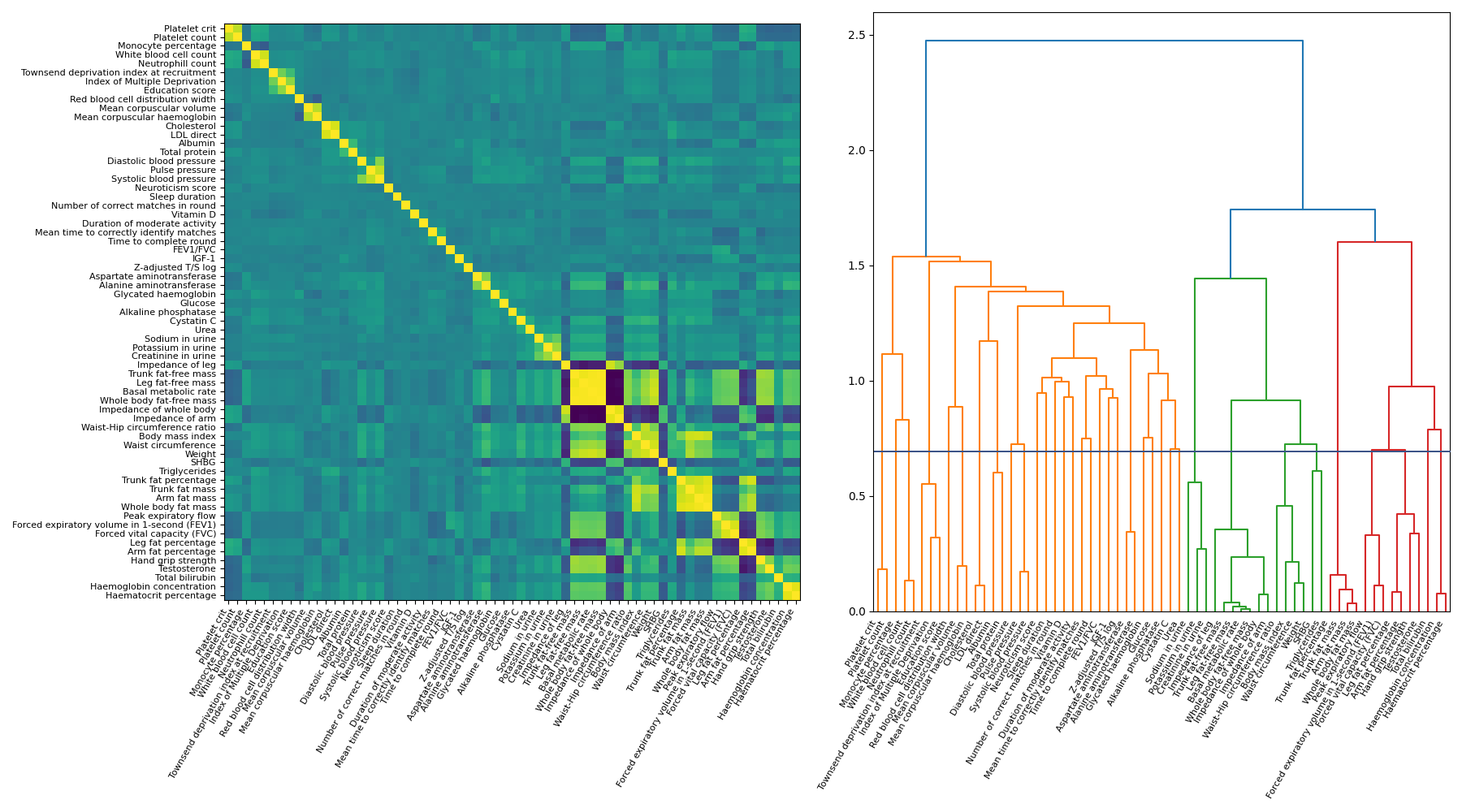


Left, the heatmap of spearman rank-order correlation between each pair of the top 50 candidate predictors of biological age model. Right, the dendrogram of hierarchical clustering based on the calculated correlations. The horizontal line at 0.7 was the cutoff of the clusters, and only one candidate predictor was chosen within each group of cluster.

FEV1: forced expiratory volume in 1-second; FVC: forced vital capacity; IGF-1: insulin growth factor 1; LDL: low-density lipoprotein; SHBG: sex hormone binding globulin

**Figure S3. Model performance generated by lasso regression.**


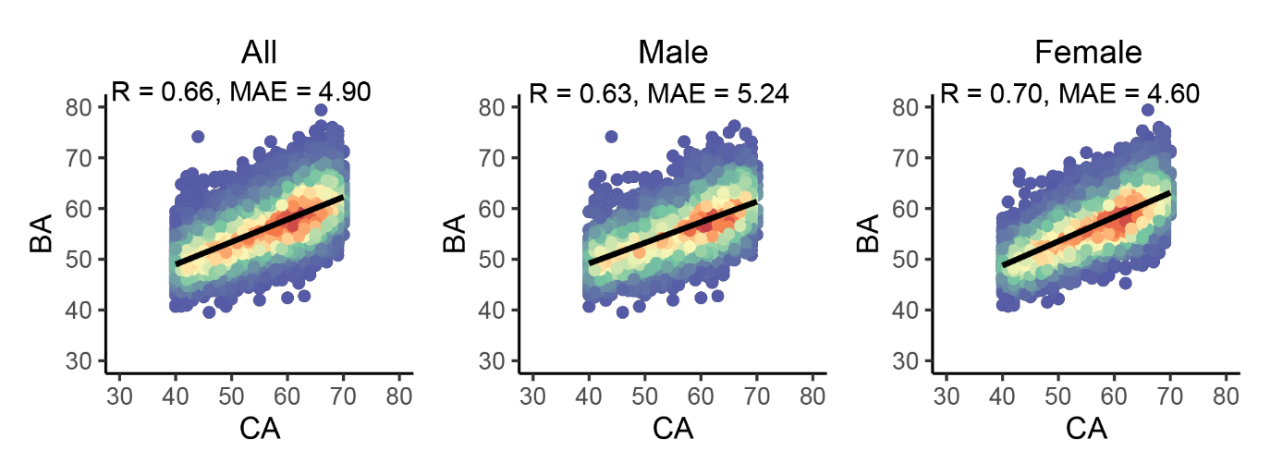


The scatter plot showing the distributions of biological age and chronological age of the participants. Each scatter indicated a single participant. The MAE and correlation coefficient of the model was shown in the left top part of the plot.

**Figure S4.** **The associations of the modifiable factors and biological age gap in healthy male participants.**


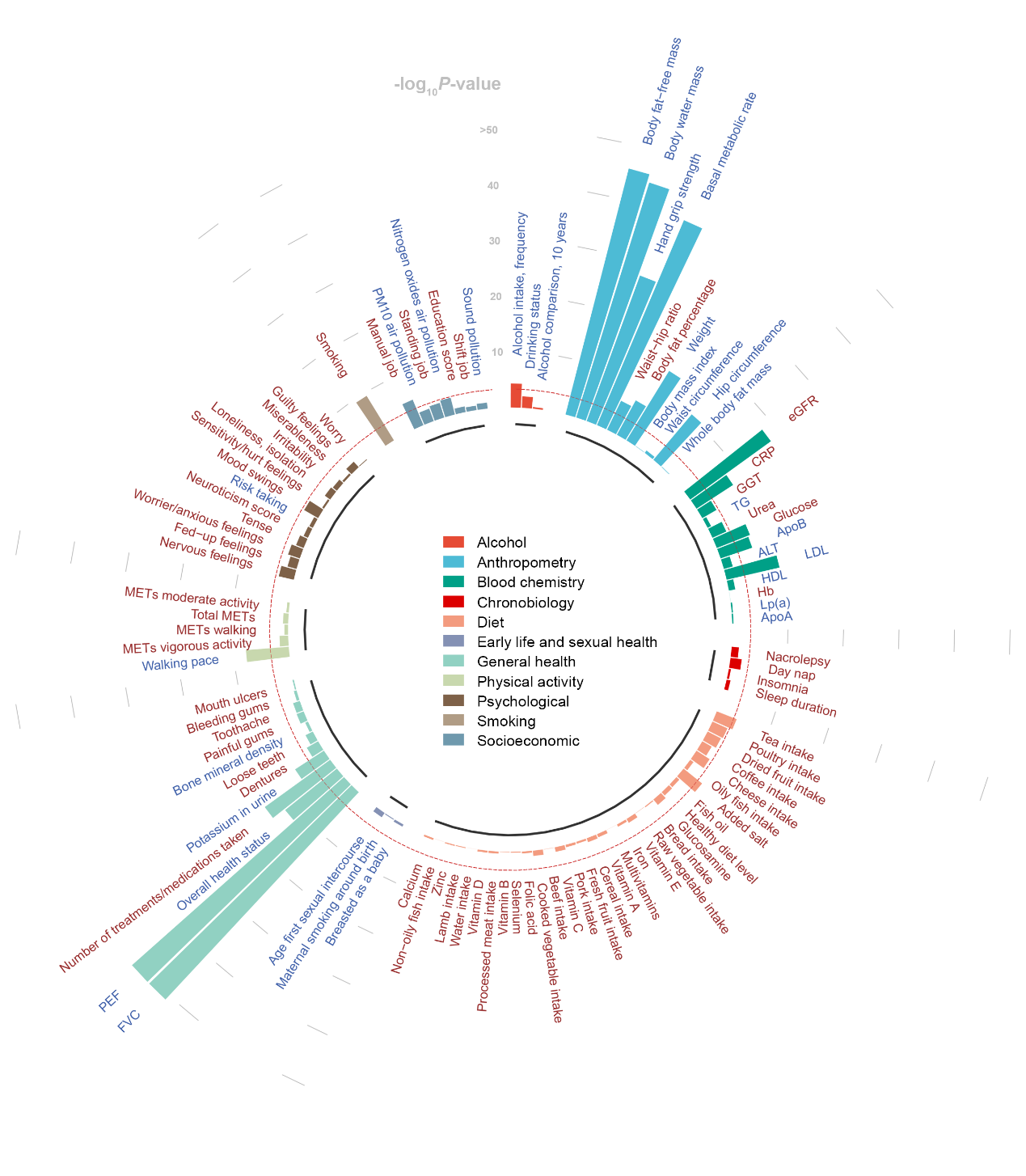


The circular barplot showing the associations of the modifiable factors with biological age gap. The association with *p*-value less than 1×10^-50^ were rounded to 1×10^-50^. The red dash line indicated the threshold of adjusted *p*-value (4.85×10^-4^). The modifiable factors were filled with different colors based on the categories. The red text indicated positive association with age gap (*β* > 0), and the light blue text indicated negative association with age gap (*β* < 0).

ALT: alanine aminotransferase; ApoA: apolipoprotein A; ApoB: apolipoprotein B; CRP: C-reactive protein; eGFR: estimated glomerular filtration rate; FVC: forced vital capacity; GGT: gamma glutamyltransferase; Hb: haemoglobin concentration; HDL: high-density lipoprotein cholesterol; LDL: low-density lipoprotein cholesterol; Lp(a): lipoprotein A; MET: Metabolic Equivalent Task; PEF: peak expiratory flow; PM10: particulate matter with diameter less than or equal to 10 micrometers; TG: triglycerides.

**Figure S5. The associations of the modifiable factors and biological age gap in healthy female participants.**


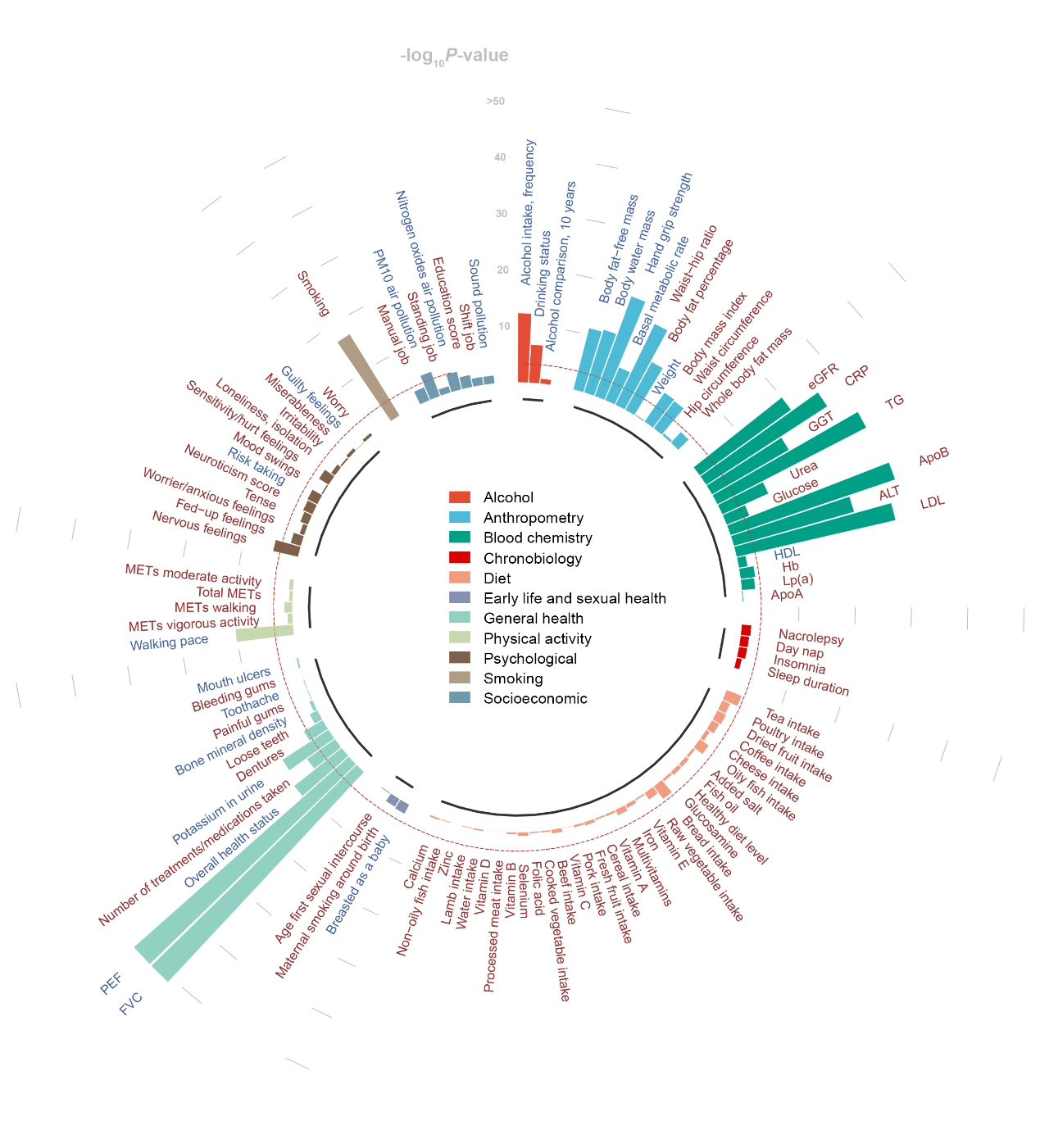


The circular barplot showing the associations of the modifiable factors with biological age gap. The association with *p*-value less than 1×10^-50^ were rounded to 1×10^-50^. The red dash line indicated the threshold of adjusted *p*-value (4.85×10^-4^). The modifiable factors were filled with different colors based on the categories. The red text indicated positive association with age gap (*β* > 0), and the light blue text indicated negative association with age gap (*β* < 0).

ALT: alanine aminotransferase; ApoA: apolipoprotein A; ApoB: apolipoprotein B; CRP: C-reactive protein; eGFR: estimated glomerular filtration rate; FVC: forced vital capacity; GGT: gamma glutamyltransferase; Hb: haemoglobin concentration; HDL: high-density lipoprotein cholesterol; LDL: low-density lipoprotein cholesterol; Lp(a): lipoprotein A; MET: Metabolic Equivalent Task; PEF: peak expiratory flow; PM10: particulate matter with diameter less than or equal to 10 micrometers; TG: triglycerides.

**Figure S6. Shared modifiable factors for biological age gap.**


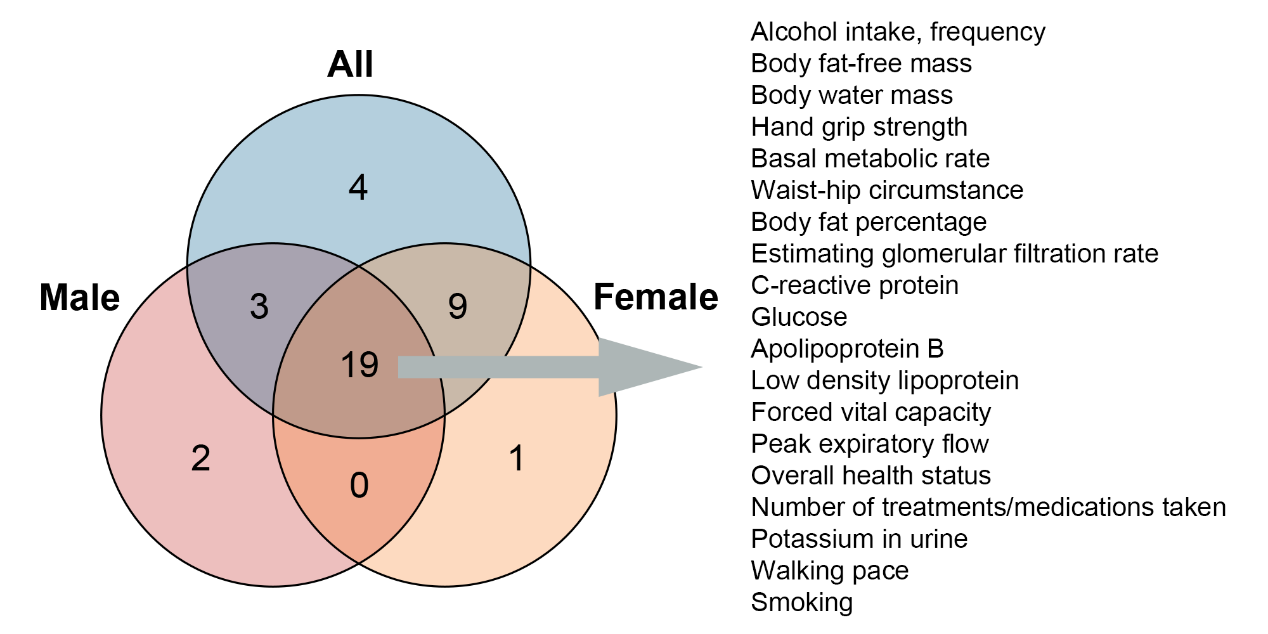


The venn plot showing the shared and specific modifiable factors for biological age gap in all, male, and female participants. The 19 shared modifiable factors for biological age gap were shown on the right.

**Figure S7. Quantile-quantile plot for GWAS analysis of biological age gap in healthy participants.**


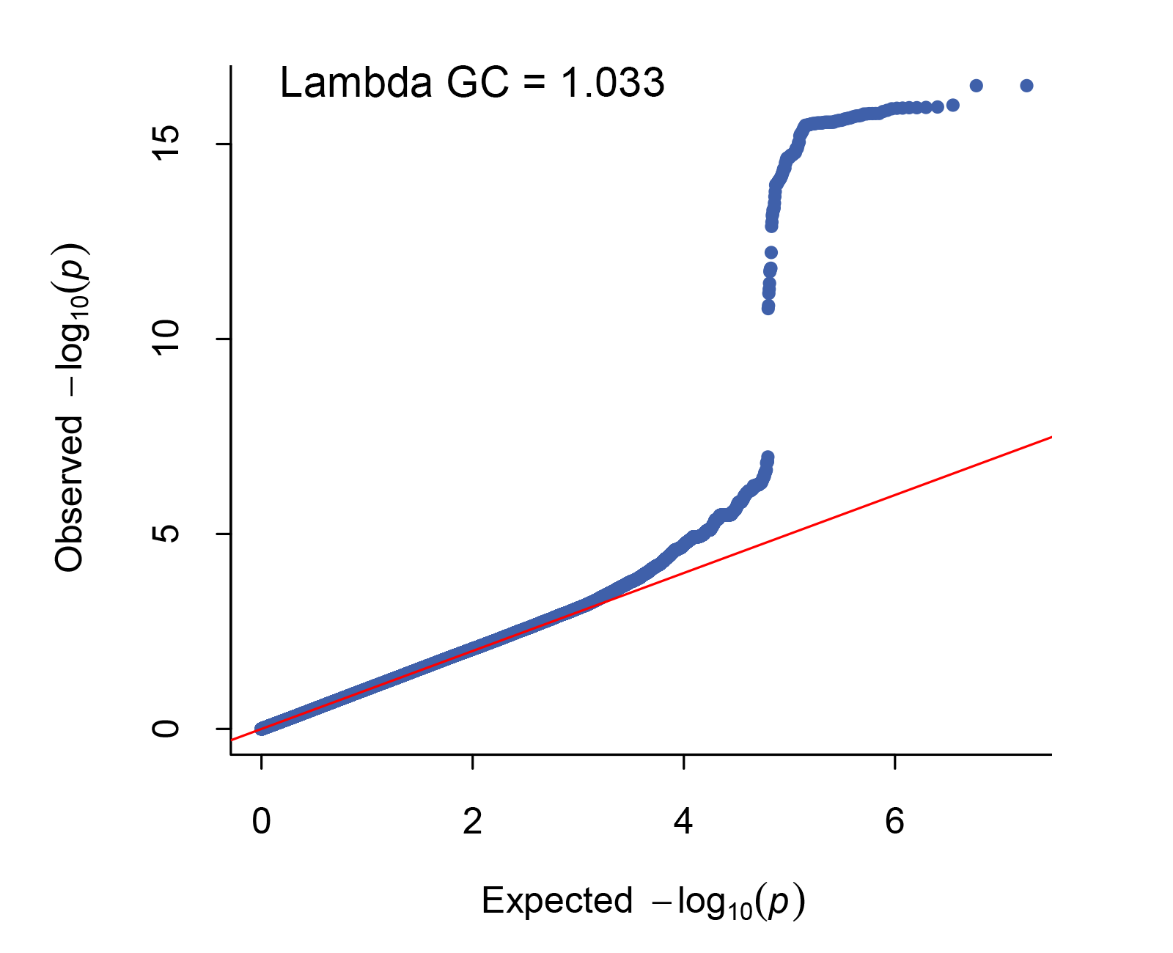


**Figure S8. Quantile-quantile plot for ExWAS analysis of biological age gap in healthy participants.**


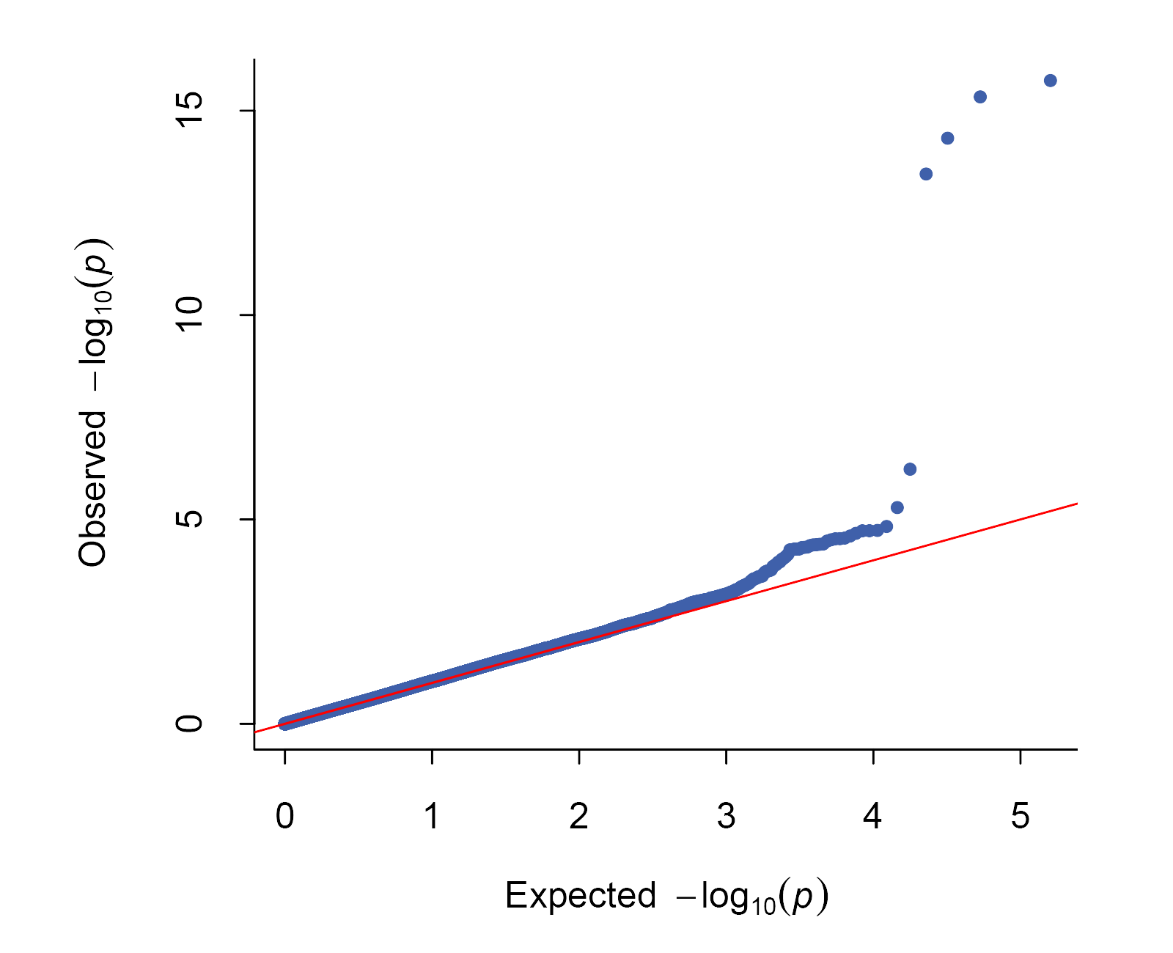


**Figure S9. ExWAS gene-based rare variant analysis of biological age gap.**


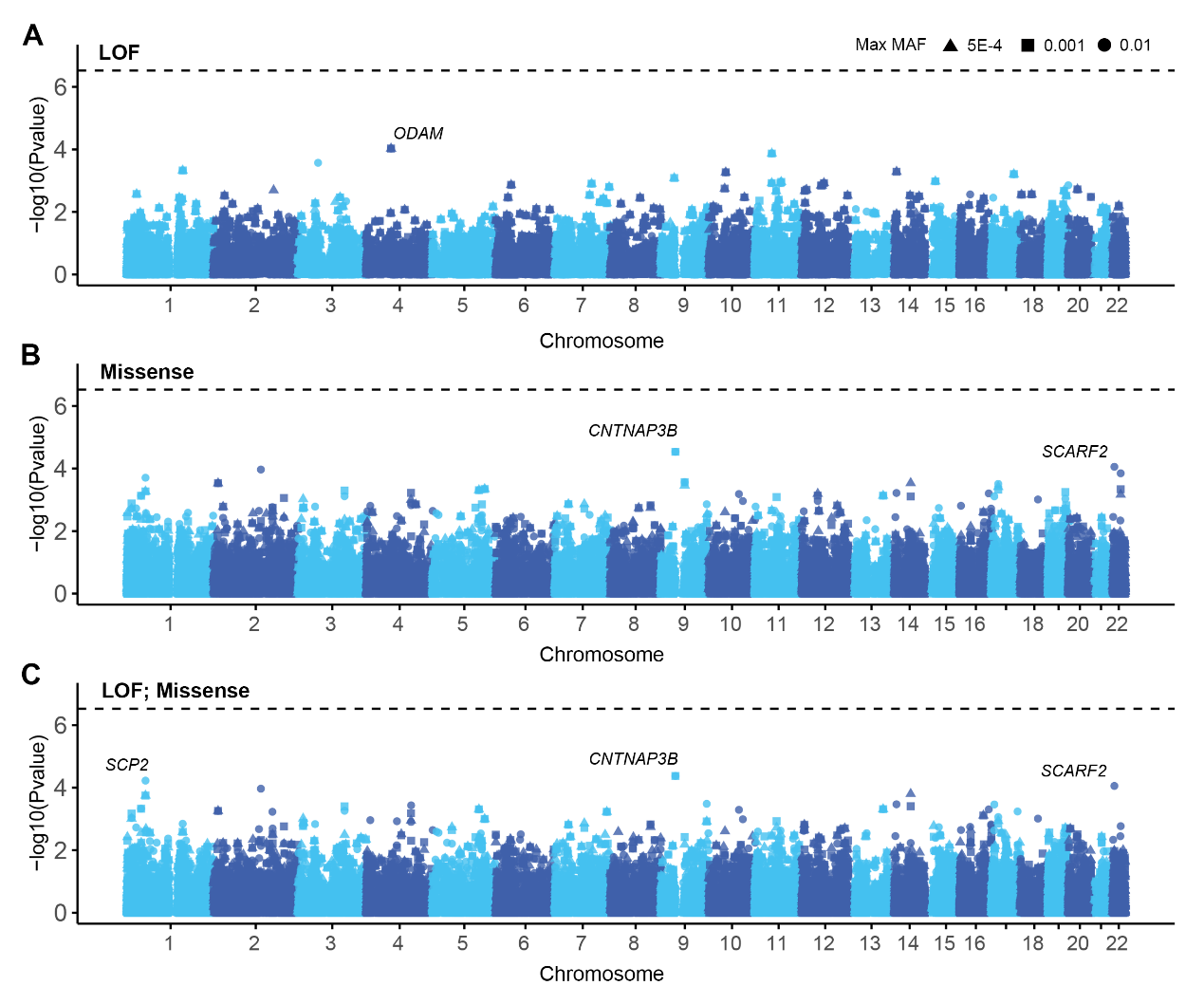


The manhattan plots showing the results of ExWAS gene-based rare variant (A: loss-of-function; B: missense; C: loss-of-function and/or missense) analysis of biological age gap in healthy participants. The genes with *p* < 10^-4^ were highlighted.

**Figure S10. GO and KEGG analysis of the mapped genes associated with biological age gap.**


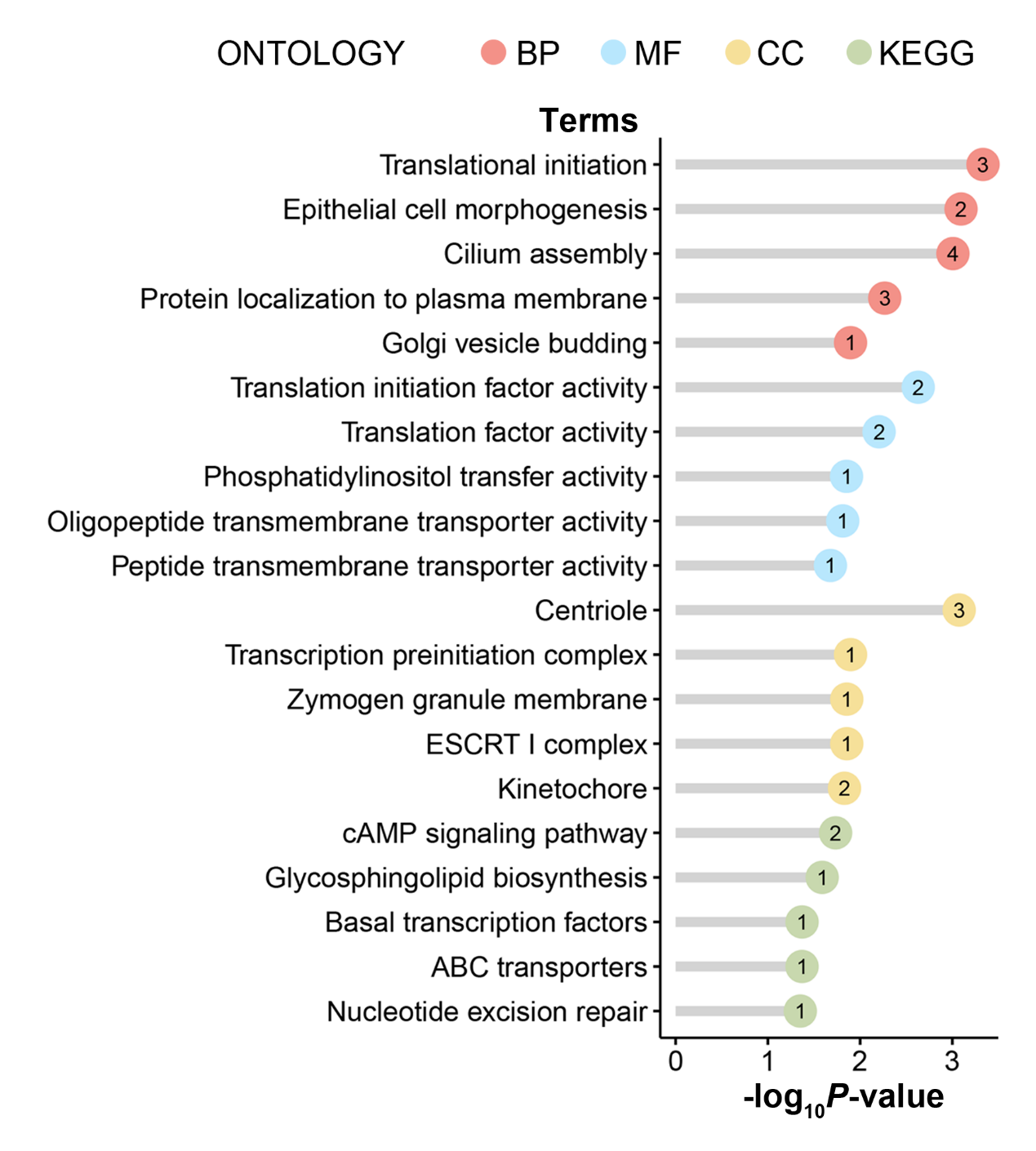


The *x*-axis indicated the -log10 P-value of the terms in GO or KEGG analysis. The *y*-axis indicated the terms in GO and KEGG.

BP: biological process; CC: cellular component; KEGG: Kyoto Encyclopedia of Genes and Genomes; MF: molecular function.

**Figure S11.** **Regional association plot of rs3761280 in healthy and unhealthy White British participants.**


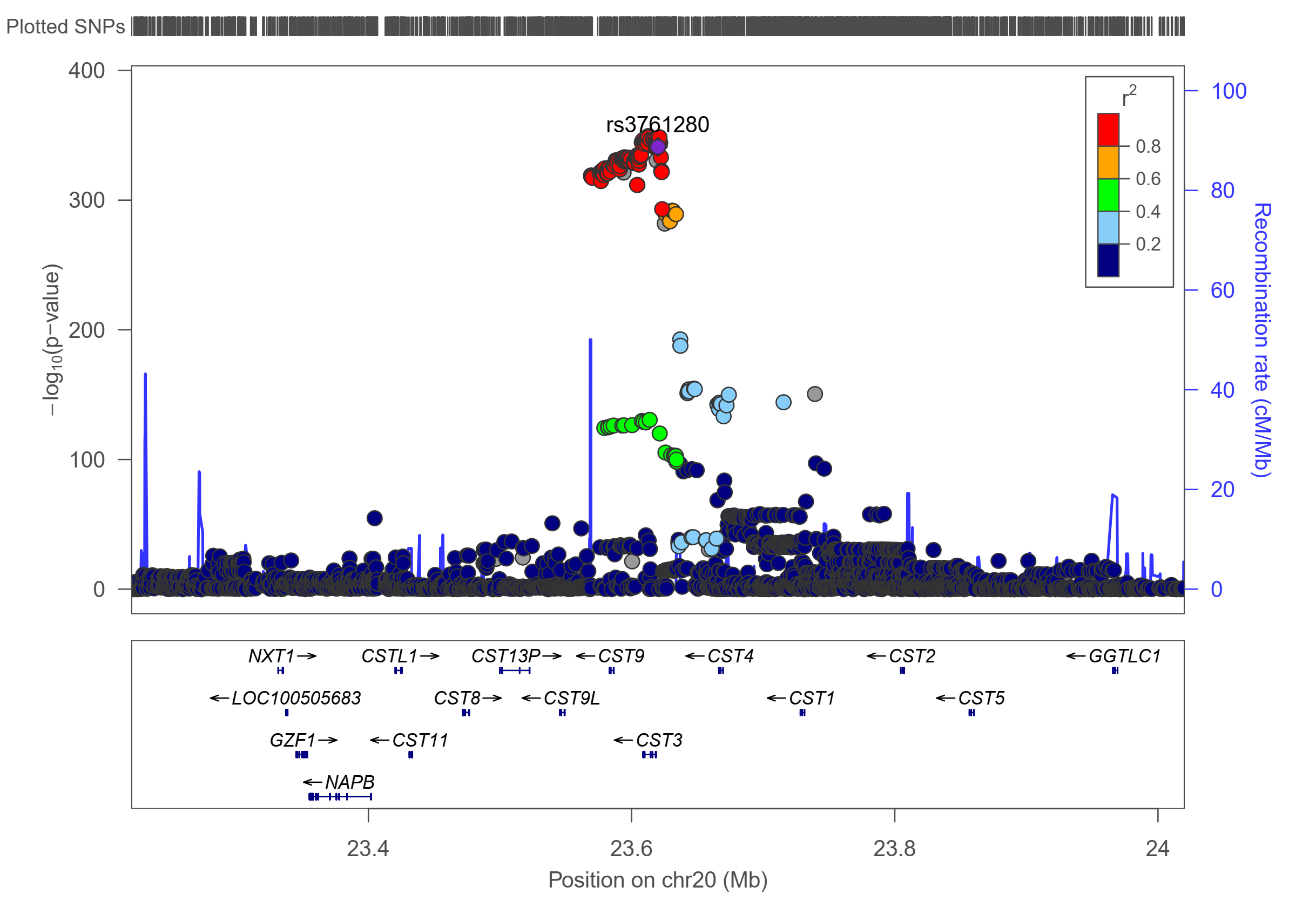


The region covering rs3761280 ± 0.4 Mb were shown in the locus zoom plot. The SNP rs3761280 was highlighted and filed with purple color. The colors within the dots indicated the levels of linkage disequilibrium.

**Table S1. Demographic characteristics of the healthy participants in the UK Biobank.**

| **Characteristics** | **Healthy participants**  **(*n* = 59,316)** |
| --- | --- |
| Age, years | 57 (50-63) |
| Gender |  |
| Male | 27,786 (46.8%) |
| Female | 31,530 (53.2%) |
| Ethnicity |  |
| White | 55,609 (93.8%) |
| Black | 997 (1.7%) |
| Asian | 1,401 (2.4%) |
| Mixed | 380 (0.6%) |
| Other | 1,523 (2.6%) |
| Smoking status |  |
| Current | 6,375 (10.7%) |
| Previous | 20,043 (33.8%) |
| Never | 32,898 (55.5%) |
| Drinking |  |
| Current | 54,580 (92.0%) |
| Previous | 2,088 (3.5%) |
| Never | 2,648 (4.5%) |

**Table S2. Details about the phenotypes considered in constructing biological age model.**

| **Phenotypes** | **Subject** | **FieldID** | **category** | **Note** |
| --- | --- | --- | --- | --- |
| Pulse rate, automated reading | 472,277 | 102 | Cardiovascular |  |
| Diastolic blood pressure | 472,277 | 4079 | Cardiovascular |  |
| Systolic blood pressure | 472,272 | 4080 | Cardiovascular |  |
| **Pulse pressure** | 472,272 | Derived | Cardiovascular | SBP – DBP |
| Mean arterial pressure | 472,272 | Derived | Cardiovascular | DBP + (SBP-DBP)/3 |
| Number of columns displayed in round | 497,764 | 396 | Cognition and mental |  |
| Number of rows displayed in round | 497,764 | 397 | Cognition and mental |  |
| Number of correct matches in round | 497,764 | 398 | Cognition and mental |  |
| Number of incorrect matches in round | 497,764 | 399 | Cognition and mental |  |
| **Time to complete round** | 497,764 | 400 | Cognition and mental |  |
| Sleep duration | 501,500 | 1160 | Cognition and mental |  |
| **Mean time to correctly identify matches** | 496,565 | 20023 | Cognition and mental |  |
| Neuroticism score | 401,483 | 20127 | Cognition and mental |  |
| **Z-adjusted T/S log (Telomere length)** | 472,506 | 22192 | Genomic |  |
| **Albumin** | 429,978 | 30600 | Hepatic |  |
| **Alkaline phosphatase** | 469,494 | 30610 | Hepatic |  |
| Alanine aminotransferase | 469,293 | 30620 | Hepatic |  |
| Apolipoprotein A | 427,413 | 30630 | Hepatic |  |
| Apolipoprotein B | 467,100 | 30640 | Hepatic |  |
| **Aspartate aminotransferase** | 467,689 | 30650 | Hepatic |  |
| Direct bilirubin | 398,518 | 30660 | Hepatic |  |
| Gamma glutamyltransferase | 469,234 | 30730 | Hepatic |  |
| Total bilirubin | 467,453 | 30840 | Hepatic |  |
| **SHBG** | 425,749 | 30830 | Hormonal |  |
| Testosterone | 425,106 | 30850 | Hormonal |  |
| White blood cell count | 478,057 | 30000 | Immune |  |
| Red blood cell count | 478,062 | 30010 | Immune |  |
| Haemoglobin concentration | 478,062 | 30020 | Immune |  |
| Haematocrit percentage | 478,062 | 30030 | Immune |  |
| **Mean corpuscular volume** | 478,060 | 30040 | Immune |  |
| Mean corpuscular haemoglobin | 478,059 | 30050 | Immune |  |
| Mean corpuscular haemoglobin concentration | 478,055 | 30060 | Immune |  |
| Red blood cell distribution width | 478,060 | 30070 | Immune |  |
| Platelet count | 478,059 | 30080 | Immune |  |
| **Platelet crit** | 478,055 | 30090 | Immune |  |
| Mean platelet volume | 478,054 | 30100 | Immune |  |
| Platelet distribution width | 478,054 | 30110 | Immune |  |
| Lymphocyte count | 477,173 | 30120 | Immune |  |
| Monocyte count | 477,173 | 30130 | Immune |  |
| Neutrophill count | 477,173 | 30140 | Immune |  |
| Eosinophill count | 477,173 | 30150 | Immune |  |
| Basophill count | 477,173 | 30160 | Immune |  |
| Nucleated red blood cell count | 477,162 | 30170 | Immune |  |
| Lymphocyte percentage | 477,179 | 30180 | Immune |  |
| Monocyte percentage | 477,179 | 30190 | Immune |  |
| Neutrophill percentage | 477,179 | 30200 | Immune |  |
| Eosinophill percentage | 477,179 | 30210 | Immune |  |
| Basophill percentage | 477,179 | 30220 | Immune |  |
| Nucleated red blood cell percentage | 477,158 | 30230 | Immune |  |
| Reticulocyte percentage | 469,747 | 30240 | Immune |  |
| Reticulocyte count | 469,747 | 30250 | Immune |  |
| Mean reticulocyte volume | 469,746 | 30260 | Immune |  |
| Mean sphered cell volume | 469,748 | 30270 | Immune |  |
| Immature reticulocyte fraction | 469,746 | 30280 | Immune |  |
| High light scatter reticulocyte percentage | 469,748 | 30290 | Immune |  |
| High light scatter reticulocyte count | 469,747 | 30300 | Immune |  |
| C-reactive protein | 468,460 | 30710 | Immune |  |
| Body fat percentage | 491,982 | 23099 | Metabolic |  |
| Whole body fat mass | 491,417 | 23100 | Metabolic |  |
| Whole body fat-free mass | 492,214 | 23101 | Metabolic |  |
| Whole body water mass | 492,257 | 23102 | Metabolic |  |
| Basal metabolic rate | 492,243 | 23105 | Metabolic |  |
| Leg fat percentage | 492,226 | Derived | Metabolic | Average of left and right |
| Leg fat mass | 492,191 | Derived | Metabolic | Average of left and right |
| **Leg fat-free mass** | 492,186 | Derived | Metabolic | Average of left and right |
| Arm fat percentage | 492,080 | Derived | Metabolic | Average of left and right |
| **Arm fat mass** | 492,038 | Derived | Metabolic | Average of left and right |
| Arm fat-free mass | 492,022 | Derived | Metabolic | Average of left and right |
| Trunk fat percentage | 491,960 | 23127 | Metabolic |  |
| Trunk fat mass | 491,933 | 23128 | Metabolic |  |
| Trunk fat-free mass | 491,849 | 23129 | Metabolic |  |
| Calcium | 429,835 | 30680 | Metabolic |  |
| **Cholesterol** | 469,481 | 30690 | Metabolic |  |
| Glucose | 429,470 | 30740 | Metabolic |  |
| **Glycated haemoglobin** | 466,394 | 30750 | Metabolic |  |
| HDL cholesterol | 429,774 | 30760 | Metabolic |  |
| **Insulin growth factor-1** | 466,933 | 30770 | Metabolic |  |
| LDL direct | 468,598 | 30780 | Metabolic |  |
| Lipoprotein A | 375,546 | 30790 | Metabolic |  |
| Phosphate | 429,154 | 30810 | Metabolic |  |
| Total protein | 429,507 | 30860 | Metabolic |  |
| Triglycerides | 469,106 | 30870 | Metabolic |  |
| **Vitamin D** | 448,246 | 30890 | Metabolic |  |
| Hand grip strength | 498,976 | Derived | Metabolic | Average of left and right |
| Waist circumference | 500,226 | 48 | Muscoskeletal |  |
| Hip circumference | 500,167 | 49 | Muscoskeletal |  |
| Waist-Hip circumference ratio | 500,167 | Derived | Muscoskeletal | Waist/Hip circumference |
| Seated height | 496,110 | 51 | Muscoskeletal |  |
| Body mass index | 499,282 | 21001 | Muscoskeletal |  |
| Weight | 499,612 | 21002 | Muscoskeletal |  |
| Impedance of whole body | 492,203 | 23106 | Muscoskeletal |  |
| Impedance of leg | 492,226 | Derived | Muscoskeletal | Average of left and right |
| Impedance of arm | 492,194 | Derived | Muscoskeletal | Average of left and right |
| Number of days/week walked 10+ minutes | 501,513 | 864 | Physical |  |
| Duration of walks | 477,764 | 874 | Physical |  |
| Duration of moderate activity | 410,943 | 894 | Physical |  |
| Summed days activity | 413,939 | 22033 | Physical |  |
| Summed minutes activity | 402,284 | 22034 | Physical |  |
| Summed MET minutes per week for all activity | 402,284 | 22040 | Physical |  |
| Health score | 432,626 | 26413 | Physical |  |
| Exposure to tobacco smoke at home | 462,665 | 1269 | Pulmonary |  |
| Exposure to tobacco smoke outside home | 462,665 | 1279 | Pulmonary |  |
| Forced vital capacity (FVC) | 453,589 | 3062 | Pulmonary |  |
| **Forced expiratory volume in 1-second (FEV1)** | 453,589 | 3063 | Pulmonary |  |
| Peak expiratory flow | 453,589 | 3064 | Pulmonary |  |
| FEV1/FVC | 453,589 | Derived | Pulmonary | FEV1/FVC |
| Creatinine in urine | 484,195 | 30510 | Renal |  |
| Potassium in urine | 483,174 | 30520 | Renal |  |
| **Sodium in urine** | 483,163 | 30530 | Renal |  |
| Urea | 469,163 | 30670 | Renal |  |
| Creatinine | 469,249 | 30700 | Renal |  |
| **Cystatin C** | 469,450 | 30720 | Renal |  |
| Urate | 468,917 | 30880 | Renal |  |
| **Townsend deprivation index at recruitment** | 501,763 | 189 | Social |  |
| Index of Multiple Deprivation | 432,626 | 26410 | Social |  |
| Education score | 432,626 | 26414 | Social |  |

The phenotypes included in the biological model were marked as bold red.

**Table S3. The biological age model performance at different numbers of predictors.**

| **Predictors** | **MAE** | **△MAE** |
| --- | --- | --- |
| Pulse pressure | 6.2189 | NA |
| Cystatin C | 5.8167 | 0.402 |
| Forced expiratory volume in 1-second (FEV1) | 5.5129 | 0.304 |
| Mean time to correctly identify matches | 5.3070 | 0.206 |
| Time to complete round | 5.2063 | 0.101 |
| Glycated haemoglobin | 5.1185 | 0.088 |
| IGF-1 | 5.0476 | 0.071 |
| Z-adjusted T/S log | 4.9932 | 0.054 |
| Leg fat-free mass | 4.9173 | 0.076 |
| Townsend deprivation index at recruitment | 4.8436 | 0.074 |
| Mean corpuscular volume | 4.7865 | 0.057 |
| SHBG | 4.7435 | 0.043 |
| Sodium in urine | 4.7059 | 0.038 |
| Vitamin D | 4.6740 | 0.032 |
| Platelet crit | 4.6393 | 0.035 |
| Alkaline phosphatase | 4.6083 | 0.031 |
| Aspartate aminotransferase | 4.6034 | 0.005 |
| Albumin | 4.5829 | 0.020 |
| Cholesterol | 4.5571 | 0.026 |
| Arm fat mass | 4.4876 | 0.069 |
| Neuroticism score | 4.4732 | 0.014 |
| Glucose | 4.4623 | 0.011 |
| Monocyte percentage | 4.4589 | 0.003 |
| Haemoglobin concentration | 4.4488 | 0.010 |
| Number of correct matches in round | 4.4454 | 0.003 |
| Sleep duration | 4.4341 | 0.011 |
| White blood cell count | 4.4261 | 0.008 |
| Duration of moderate activity | 4.4193 | 0.007 |
| Total bilirubin | 4.4096 | 0.010 |
| Red blood cell distribution width | 4.4064 | 0.003 |

IGF: insulin growth factor; NA: not applicable; SHBG: sex hormone binding globulin.

**Table S4. Feature selection by lasso regression.**

| **Phenotypes** |
| --- |
| Neuroticism score |
| Summed days activity |
| Summed minutes activity |
| Number of correct matches in round |
| Number of incorrect matches in round |
| Sleep duration |
| Exposure to tobacco smoke at home |
| Exposure to tobacco smoke outside home |
| Hand grip strength |
| Waist circumference |
| Pulse rate, automated reading |
| Townsend deprivation index at recruitment |
| Diastolic blood pressure |
| Mean time to correctly identify matches |
| Z-adjusted T/S log (Telomere length) |
| Body fat percentage |
| Whole body fat-free mass |
| White blood cell count |
| Red blood cell count |
| Mean corpuscular volume |
| Mean corpuscular haemoglobin concentration |
| Platelet count |
| Mean platelet volume |
| Lymphocyte count |
| Monocyte count |
| Eosinophill count |
| Basophill count |
| Nucleated red blood cell count |
| Reticulocyte percentage |
| Creatinine in urine |
| Albumin |
| Alkaline phosphatase |
| Alanine aminotransferase |
| Apolipoprotein A |
| Apolipoprotein B |
| Direct bilirubin |
| Urea |
| Creatinine |
| C-reactive protein |
| Glucose |
| Glycated haemoglobin |
| Insulin growth factor-1 |
| Lipoprotein A |
| Phosphate |
| SHBG |
| Vitamin D |
| FEV1/FVC |
| Pulse pressure |

**Table S5. Demographic characteristics of the unhealthy participants in the UK Biobank.**

| **Characteristics** | **Unhealthy participants**  **(*n* = 257,336)** |
| --- | --- |
| Age, years | 58 (50-63) |
| Gender |  |
| Male | 120,132 (46.7%) |
| Female | 137,204 (53.3%) |
| Ethnicity |  |
| White | 244,586 (95.0%) |
| Black | 3,256 (1.3%) |
| Asian | 5,044 (2.0%) |
| Mixed | 1,484 (0.6%) |
| Other | 2,966 (1.2%) |
| Smoking status |  |
| Current | 25,995 (10.1%) |
| Previous | 89,488 (34.8%) |
| Never | 141,853 (55.1%) |
| Drinking |  |
| Current | 238,373 (92.6%) |
| Previous | 8,557 (3.3%) |
| Never | 10,406 (4.0%) |
| Education score | 9.71 (3.79-20.74) |
| Townsend deprivation index | -1.41 (3.01) |
| Overall health rating |  |
| Excellent | 44,235 (17.2%) |
| Good | 152,207 (59.1%) |
| Fair | 51,576 (20.0%) |
| Poor | 9,318 (3.6%) |
| Number of treatments/medications | 2 (0-4) |

**Table S6. The common health-related outcomes included in the longitudinal analysis.**

| **Outcomes** | **ICD-10** |  |
| --- | --- | --- |
| Infections | A01-B89 |  |
| Bacterial infections | A01-A79 |  |
| Viral infections | A80-B34 |  |
| Cancer | C00-C97 |  |
| Colorectal cancer | C18, C20 |  |
| Lung cancer | C34 |  |
| Melanoma | C43-C44 |  |
| Breast cancer | C50 |  |
| Prostate cancer | C61 |  |
| Kidney cancer | C64 |  |
| Brain cancer | C71 |  |
| Leukaemia | C81-C96 |  |
| Diseases of the blood | D50-D89 |  |
| Anaemia | D50-D64 |  |
| Endocrine diseases | E00-E90 |  |
| Diabetes | E10-E14 |  |
| Obesity | E66 |  |
| Mental and behavioural disorders | F00-F99 |  |
| Dementia | F00-F03, G30, G31 |  |
| Disorders due to substance abuse | F10-F19 |  |
| Psychotic disorders | F20-F29 |  |
| Mood disorders | F30-F39 |  |
| Neurotic disorders | F40-F48 |  |
| Diseases of the nervous system | G00-G99 |  |
| Parkinson disease | G20 |  |
| Multiple sclerosis | G35 |  |
| Epilepsy | G40-G42 |  |
| Headaches | G43-G44 |  |
| Transient ischaemic attack | G45-G46 |  |
| Sleep disorders | G47 |  |
| Diseases of the eye | H00-H59 |  |
| Diseases of the ear | H60-H99 |  |
| Diseases of the circulatory system | I00-I99 |  |
| Hypertension | I10-I15 |  |
| Ischaemic heart disease | I20-I25 |  |
| Angina pectoris | I20 |  |
| Myocardial infarction | I21 |  |
| Pulmonary embolism | I26 |  |
| Arrhythmias | I46-I49 |  |
| Heart failure | I50 |  |
| Cerebrovascular diseases | I60-I69 |  |
| Stroke | I60-I61, I63-I64 |  |
| Intracerebral haemorrhage | I61 |  |
| Cerebral infarction | I63 |  |
| Atherosclerosis | I70 |  |
| Deep vein thrombosis | I80-I82 |  |
| Diseases of the respiratory system | J00-J99 |  |
| Influenza and Pneumonia | J09-J18 |  |
| Chronic obstructive bronchitis | J43-J44, J47 |  |
| Asthma | J45-J46 |  |
| Diseases of the digestive system | K00-K93 |  |
| Appendicitis | K35 |  |
| Inflammatory bowel disease | K50-K52 |  |
| Alcoholic liver disease | K70 |  |
| Diseases of liver | K70-K77 |  |
| Pancreatitis | K85 |  |
| Diseases of the skin | L00-L99 |  |
| Infections and excema | L00-L08, L20-L30 |  |
| Diseases of the musculoskeletal system | M00-M99 |  |
| Rheumatoid arthritis and related disorders | M05-M06, M08, M13, M30-M35, M45 |  |
| Gout | M10 |  |
| Osteoarthritis | M15-M19 |  |
| Sciatica | M50-M51 |  |
| Back pain | M54 |  |
| Soft tissue disorders | M60-M79 |  |
| Diseases of the genitourinary system | N00-N99 |  |
| Renal failure | N17-N19 |  |
| Pregnancy complications | O00-O03, O05-O29 |  |
| Circulatory and respiratory symptoms | R00-R09 |  |
| Digestive and abdominal symptoms | R10-R19 |  |

ICD: international classification of diseases.

**Table S7.** **Longitudinal associations of age gap with the risk of common health-related outcomes.**

| **Characteristics** | **ICD** | **Event/N** | **Q1** | | **Q2 & Q3** | | **Q4** | |
| --- | --- | --- | --- | --- | --- | --- | --- | --- |
|  |  |  | **HR (95% CI)** | **P** | **HR (95% CI)** | **P** | **HR (95% CI)** | **P** |
| Infections | A01-B89 | 28062 / 213137 | 0.95 (0.92-0.98) | 0.001 | Ref | Ref | 1.06 (1.02-1.11) | 0.001 |
| Bacterial infections | A01-A79 | 18012 / 246458 | 0.90 (0.87-0.94) | 2.70E-07 | Ref | Ref | 1.11 (1.06-1.16) | 4.14E-05 |
| Viral infections | A80-B34 | 9791 / 230308 | 0.96 (0.91-1.01) | 0.125 | Ref | Ref | 1.00 (0.94-1.07) | 0.949 |
| Cancer | C00-C97 | 34591 / 238658 | 1.05 (1.02-1.08) | 1.49E-04 | Ref | Ref | 0.99 (0.96-1.03) | 0.704 |
| Colorectal cancer | C18, C20 | 2908 / 255349 | 1.07 (0.98-1.17) | 0.113 | Ref | Ref | 1.01 (0.88-1.16) | 0.876 |
| Lung cancer | C34 | 2135 / 256327 | 0.90 (0.81-1.01) | 0.064 | Ref | Ref | 1.13 (0.96-1.34) | 0.141 |
| Melanoma | C43-C44 | 14280 / 249811 | 1.03 (0.99-1.07) | 0.172 | Ref | Ref | 0.98 (0.92-1.04) | 0.520 |
| Breast cancer | C50 | 4876 / 252046 | 1.07 (0.99-1.15) | 0.098 | Ref | Ref | 0.93 (0.86-1.02) | 0.113 |
| Prostate cancer | C61 | 6095 / 254555 | 1.18 (1.12-1.26) | 1.20E-08 | Ref | Ref | 0.93 (0.83-1.03) | 0.155 |
| Kidney cancer | C64 | 796 / 256165 | 1.18 (0.99-1.39) | 0.060 | Ref | Ref | 0.87 (0.67-1.13) | 0.304 |
| Brain cancer | C71 | 406 / 256374 | 1.03 (0.80-1.31) | 0.835 | Ref | Ref | 0.98 (0.70-1.37) | 0.889 |
| Leukaemia | C81-C96 | 2598 / 255350 | 0.96 (0.88-1.06) | 0.435 | Ref | Ref | 1.07 (0.92-1.23) | 0.386 |
| Diseases of the blood | D50-D89 | 21477 / 243329 | 0.93 (0.89-0.96) | 2.47E-05 | Ref | Ref | 1.12 (1.07-1.17) | 1.94E-06 |
| Anaemia | D50-D64 | 17568 / 246445 | 0.93 (0.89-0.96) | 1.43E-04 | Ref | Ref | 1.10 (1.05-1.16) | 2.68E-04 |
| Endocrine diseases | E00-E90 | 42600 / 193465 | 0.91 (0.89-0.93) | 8.45E-13 | Ref | Ref | 1.11 (1.07-1.14) | 2.41E-10 |
| Diabetes | E10-E14 | 10443 / 243954 | 0.73 (0.69-0.78) | 2.10E-28 | Ref | Ref | 1.50 (1.41-1.60) | 1.47E-34 |
| Obesity | E66 | 17702 / 249824 | 1.11 (1.07-1.16) | 4.31E-07 | Ref | Ref | 0.98 (0.93-1.03) | 0.372 |
| Mental and behavioural disorders | F00-F99 | 26847 / 214915 | 0.93 (0.90-0.96) | 3.32E-05 | Ref | Ref | 1.07 (1.03-1.12) | 3.01E-04 |
| Dementia | F00-F03, G30, G31 | 4029 / 256216 | 0.84 (0.78-0.91) | 5.22E-06 | Ref | Ref | 1.34 (1.14-1.57) | 3.95E-04 |
| Disorders due to substance abuse | F10-F19 | 13247 / 243754 | 0.91 (0.87-0.96) | 0.001 | Ref | Ref | 1.23 (1.17-1.30) | 5.26E-15 |
| Psychotic disorders | F20-F29 | 508 / 255903 | 0.80 (0.61-1.05) | 0.105 | Ref | Ref | 1.38 (1.04-1.84) | 0.026 |
| Mood disorders | F30-F39 | 9553 / 235477 | 1.01 (0.95-1.07) | 0.734 | Ref | Ref | 1.00 (0.94-1.06) | 0.934 |
| Neurotic disorders | F40-F48 | 12387 / 241646 | 1.00 (0.95-1.06) | 0.945 | Ref | Ref | 1.00 (0.95-1.06) | 0.863 |
| Diseases of the nervous system | G00-G99 | 26956 / 220263 | 1.00 (0.97-1.03) | 0.937 | Ref | Ref | 1.01 (0.97-1.06) | 0.495 |
| Parkinson disease | G20 | 1443 / 255998 | 1.10 (0.97-1.24) | 0.137 | Ref | Ref | 0.88 (0.68-1.14) | 0.331 |
| Multiple sclerosis | G35 | 240 / 255557 | 1.09 (0.72-1.65) | 0.689 | Ref | Ref | 1.20 (0.86-1.67) | 0.295 |
| Epilepsy | G40-G42 | 1457 / 254026 | 0.89 (0.77-1.02) | 0.085 | Ref | Ref | 1.35 (1.14-1.60) | 4.70E-04 |
| Headaches | G43-G44 | 3901 / 244469 | 1.09 (0.99-1.19) | 0.078 | Ref | Ref | 0.93 (0.85-1.02) | 0.135 |
| Transient ischaemic attack | G45-G46 | 5006 / 251814 | 0.90 (0.82-0.99) | 0.023 | Ref | Ref | 0.98 (0.85-1.14) | 0.823 |
| Sleep disorders | G47 | 2894 / 254426 | 1.11 (1.03-1.20) | 0.009 | Ref | Ref | 0.94 (0.86-1.03) | 0.178 |
| Diseases of the eye | H00-H59 | 38730 / 219696 | 0.98 (0.96-1.01) | 0.130 | Ref | Ref | 1.03 (1.00-1.07) | 0.063 |
| Diseases of the ear | H60-H99 | 21557 / 223849 | 1.04 (1.01-1.08) | 0.020 | Ref | Ref | 1.04 (1.00-1.09) | 0.047 |
| Diseases of the circulatory system | I00-I99 | 44783 / 162535 | 0.86 (0.83-0.88) | 8.94E-33 | Ref | Ref | 1.20 (1.16-1.23) | 1.37E-31 |
| Hypertension | I10-I15 | 30030 / 189765 | 0.70 (0.68-0.72) | 8.76E-112 | Ref | Ref | 1.42 (1.36-1.47) | 2.86E-71 |
| Ischaemic heart disease | I20-I25 | 17392 / 244153 | 0.81 (0.78-0.84) | 2.29E-27 | Ref | Ref | 1.26 (1.20-1.33) | 1.46E-17 |
| Angina pectoris | I20 | 8055 / 247293 | 0.79 (0.75-0.84) | 5.97E-16 | Ref | Ref | 1.13 (1.05-1.22) | 0.002 |
| Myocardial infarction | I21 | 5621 / 251324 | 0.77 (0.72-0.82) | 8.97E-14 | Ref | Ref | 1.26 (1.15-1.38) | 7.41E-07 |
| Pulmonary embolism | I26 | 3767 / 254570 | 1.12 (1.03-1.21) | 0.006 | Ref | Ref | 1.07 (0.95-1.20) | 0.282 |
| Arrhythmias | I46-I49 | 18054 / 249775 | 0.94 (0.91-0.98) | 0.002 | Ref | Ref | 1.11 (1.05-1.17) | 3.23E-04 |
| Heart failure | I50 | 7165 / 255442 | 0.78 (0.73-0.82) | 6.27E-17 | Ref | Ref | 1.50 (1.36-1.65) | 4.37E-17 |
| Cerebrovascular diseases | I60-I69 | 8690 / 252534 | 0.84 (0.80-0.89) | 1.27E-10 | Ref | Ref | 1.31 (1.20-1.42) | 2.45E-10 |
| Stroke | I60-I61, I63-I64 | 5342 / 252934 | 0.87 (0.81-0.93) | 3.57E-05 | Ref | Ref | 1.29 (1.17-1.43) | 1.04E-06 |
| Intracerebral haemorrhage | I61 | 928 / 256261 | 0.80 (0.68-0.94) | 0.007 | Ref | Ref | 1.10 (0.86-1.42) | 0.442 |
| Cerebral infarction | I63 | 3896 / 255764 | 0.82 (0.76-0.89) | 7.30E-07 | Ref | Ref | 1.27 (1.12-1.43) | 2.16E-04 |
| Atherosclerosis | I70 | 1450 / 256135 | 0.79 (0.69-0.91) | 0.001 | Ref | Ref | 1.77 (1.44-2.16) | 4.12E-08 |
| Deep vein thrombosis | I80-I82 | 3757 / 250664 | 1.06 (0.98-1.16) | 0.137 | Ref | Ref | 1.09 (0.97-1.21) | 0.134 |
| Diseases of the respiratory system | J00-J99 | 33842 / 166103 | 0.92 (0.90-0.95) | 1.40E-07 | Ref | Ref | 1.11 (1.07-1.15) | 1.24E-08 |
| Influenza and Pneumonia | J09-J18 | 13825 / 247008 | 0.87 (0.83-0.91) | 8.41E-10 | Ref | Ref | 1.31 (1.24-1.39) | 6.63E-19 |
| Chronic obstructive bronchitis | J43-J44, J47 | 9429 / 252297 | 0.75 (0.71-0.79) | 3.03E-25 | Ref | Ref | 1.51 (1.40-1.62) | 8.39E-27 |
| Asthma | J45-J46 | 6487 / 227997 | 0.88 (0.83-0.95) | 5.00E-04 | Ref | Ref | 1.14 (1.05-1.23) | 0.001 |
| Diseases of the digestive system | K00-K93 | 60965 / 164805 | 1.01 (0.99-1.03) | 0.417 | Ref | Ref | 1.01 (0.98-1.03) | 0.498 |
| Appendicitis | K35 | 1314 / 255020 | 1.13 (0.97-1.32) | 0.117 | Ref | Ref | 0.96 (0.82-1.11) | 0.557 |
| Inflammatory bowel disease | K50-K52 | 8306 / 247825 | 1.02 (0.96-1.08) | 0.551 | Ref | Ref | 1.09 (1.02-1.17) | 0.010 |
| Alcoholic liver disease | K70 |  | 0.63 (0.49-0.81) | 2.63E-04 | Ref | Ref | 1.66 (1.31-2.11) | 3.58E-05 |
| Diseases of liver | K70-K77 | 7003 / 254186 | 0.90 (0.85-0.97) | 0.003 | Ref | Ref | 1.19 (1.10-1.28) | 7.28E-06 |
| Pancreatitis | K85 | 1324 / 255534 | 0.92 (0.79-1.07) | 0.275 | Ref | Ref | 0.97 (0.81-1.16) | 0.721 |
| Diseases of the skin | L00-L99 | 34286 / 190197 | 1.01 (0.98-1.04) | 0.569 | Ref | Ref | 1.02 (0.98-1.05) | 0.340 |
| Infections and excema | L00-L08, L20-L30 | 20047 / 219654 | 0.98 (0.95-1.02) | 0.383 | Ref | Ref | 1.03 (0.98-1.08) | 0.202 |
| Diseases of the musculoskeletal system | M00-M99 | 47527 / 148426 | 1.01 (0.99-1.04) | 0.398 | Ref | Ref | 1.00 (0.97-1.03) | 0.926 |
| Rheumatoid arthritis and related disorders | M05-M06, M08, M13, M30-M35, M45 | 18362 / 245443 | 1.01 (0.98-1.05) | 0.469 | Ref | Ref | 0.98 (0.93-1.03) | 0.379 |
| Gout | M10 | 4563 / 250994 | 0.84 (0.78-0.91) | 9.25E-06 | Ref | Ref | 1.15 (1.04-1.27) | 0.007 |
| Osteoarthritis | M15-M19 | 31305 / 224555 | 1.04 (1.01-1.07) | 0.009 | Ref | Ref | 0.97 (0.93-1.00) | 0.076 |
| Sciatica | M50-M51 | 6714 / 247626 | 1.05 (0.98-1.12) | 0.185 | Ref | Ref | 0.88 (0.81-0.95) | 0.001 |
| Back pain | M54 | 16089 / 233104 | 1.01 (0.97-1.05) | 0.668 | Ref | Ref | 0.97 (0.92-1.02) | 0.212 |
| Soft tissue disorders | M60-M79 | 33863 / 209158 | 1.01 (0.98-1.04) | 0.578 | Ref | Ref | 1.00 (0.97-1.03) | 0.965 |
| Diseases of the genitourinary system | N00-N99 | 40527 / 174066 | 0.96 (0.94-0.99) | 0.008 | Ref | Ref | 1.02 (0.98-1.05) | 0.339 |
| Renal failure | N17-N19 | 16937 / 252898 | 0.69 (0.66-0.72) | 1.23E-76 | Ref | Ref | 1.52 (1.43-1.61) | 6.02E-45 |
| Pregnancy complications | O00-O03, O05-O29 | 242 / 246196 | 1.38 (0.87-2.17) | 0.168 | Ref | Ref | 1.09 (0.78-1.52) | 0.604 |
| Circulatory and respiratory symptoms | R00-R09 | 29279 / 241492 | 0.94 (0.91-0.97) | 1.40E-04 | Ref | Ref | 1.11 (1.07-1.15) | 1.45E-07 |
| Digestive and abdominal symptoms | R10-R19 | 34545 / 238865 | 0.98 (0.95-1.01) | 0.164 | Ref | Ref | 1.02 (0.99-1.06) | 0.213 |

CI: confidence interval; HR: hazard ratio; ICD: international classification of diseases.

**Table S8. Longitudinal associations of age gap with the risk of all-cause mortality and cause-specific mortality.**

| **Characteristics** | **ICD** | **Event/N** | **Q1** | | **Q2 & Q3** | | **Q4** | |
| --- | --- | --- | --- | --- | --- | --- | --- | --- |
|  |  |  | **HR (95% CI)** | **P** | **HR (95% CI)** | **P** | **HR (95% CI)** | **P** |
| All cause mortality | | 17181 / 256431 | 0.86 (0.83-0.89) | 8.73E-15 | Ref | Ref | 1.27 (1.19-1.34) | 4.29E-15 |
| Infections | A01-B89 | 745 / 239995 | 0.78 (0.65-0.94) | 0.009 | Ref | Ref | 1.32 (0.99-1.76) | 0.063 |
| Bacterial infections | A01-A79 | 633 / 239883 | 0.82 (0.67-1.00) | 0.045 | Ref | Ref | 1.39 (1.01-1.93) | 0.046 |
| Viral infections | A80-B34 | 92 / 239342 | 0.72 (0.39-1.30) | 0.273 | Ref | Ref | 1.16 (0.58-2.35) | 0.677 |
| Cancer | C00-C97 | 9685 / 248935 | 0.94 (0.90-0.99) | 0.020 | Ref | Ref | 1.14 (1.05-1.23) | 0.001 |
| Colorectal cancer | C18, C20 | 708 / 239958 | 0.96 (0.80-1.16) | 0.679 | Ref | Ref | 1.34 (1.02-1.76) | 0.034 |
| Lung cancer | C34 | 1629 / 240879 | 0.81 (0.72-0.92) | 0.001 | Ref | Ref | 1.20 (0.99-1.46) | 0.065 |
| Melanoma | C43-C44 | 240 / 239490 | 1.06 (0.77-1.45) | 0.717 | Ref | Ref | 0.90 (0.56-1.44) | 0.664 |
| Breast cancer | C50 | 757 / 240007 | 1.00 (0.82-1.22) | 0.994 | Ref | Ref | 1.12 (0.89-1.41) | 0.328 |
| Prostate cancer | C61 | 705 / 239955 | 1.05 (0.89-1.25) | 0.563 | Ref | Ref | 0.81 (0.53-1.23) | 0.319 |
| Kidney cancer | C64 | 307 / 239557 | 0.78 (0.59-1.02) | 0.074 | Ref | Ref | 1.04 (0.66-1.62) | 0.875 |
| Brain cancer | C71 | 406 / 239656 | 1.01 (0.79-1.29) | 0.960 | Ref | Ref | 1.01 (0.72-1.42) | 0.944 |
| Leukaemia | C81-C96 | 1068 / 240318 | 0.83 (0.72-0.96) | 0.014 | Ref | Ref | 1.49 (1.17-1.90) | 0.001 |
| Diseases of the blood | D50-D89 | 216 / 239466 | 0.70 (0.50-0.97) | 0.034 | Ref | Ref | 1.73 (0.99-3.01) | 0.054 |
| Anaemia | D50-D64 | 69 / 239319 | 0.67 (0.38-1.20) | 0.181 | Ref | Ref | 1.12 (0.32-3.88) | 0.859 |
| Endocrine diseases | E00-E90 | 1469 / 240719 | 0.71 (0.62-0.82) | 2.59E-06 | Ref | Ref | 1.93 (1.56-2.39) | 1.30E-09 |
| Diabetes | E10-E14 | 1155 / 240405 | 0.65 (0.56-0.77) | 4.06E-07 | Ref | Ref | 2.27 (1.77-2.91) | 1.20E-10 |
| Mental and behavioural disorders | F00-F99 | 852 / 240102 | 0.76 (0.64-0.90) | 0.001 | Ref | Ref | 1.49 (1.06-2.09) | 0.022 |
| Dementia | F00-F03, G30, G31 | 1183 / 240433 | 0.81 (0.70-0.92) | 0.002 | Ref | Ref | 1.49 (1.07-2.09) | 0.019 |
| Disorders due to substance abuse | F10-F19 | 113 / 239363 | 0.49 (0.24-0.97) | 0.041 | Ref | Ref | 1.61 (0.92-2.82) | 0.093 |
| Diseases of the nervous system | G00-G99 | 1546 / 240796 | 0.88 (0.78-0.99) | 0.039 | Ref | Ref | 1.41 (1.13-1.77) | 0.002 |
| Parkinson disease | G20 | 375 / 239625 | 1.05 (0.83-1.34) | 0.678 | Ref | Ref | 0.63 (0.29-1.33) | 0.222 |
| Multiple sclerosis | G35 | 50 / 239300 | 1.49 (0.64-3.45) | 0.351 | Ref | Ref | 2.39 (0.92-6.18) | 0.072 |
| Epilepsy | G40-G42 | 64 / 239314 | 0.36 (0.17-0.79) | 0.011 | Ref | Ref | 3.43 (1.35-8.73) | 0.010 |
| Diseases of the circulatory system | I00-I99 | 5853 / 245103 | 0.75 (0.70-0.80) | 7.67E-18 | Ref | Ref | 1.54 (1.38-1.71) | 3.55E-15 |
| Hypertension | I10-I15 | 1300 / 240550 | 0.62 (0.54-0.72) | 5.35E-11 | Ref | Ref | 1.70 (1.36-2.13) | 3.30E-06 |
| Ischaemic heart disease | I20-I25 | 2611 / 241861 | 0.65 (0.59-0.72) | 2.59E-16 | Ref | Ref | 1.58 (1.35-1.85) | 1.07E-08 |
| Myocardial infarction | I21 | 807 / 240057 | 0.74 (0.62-0.89) | 0.001 | Ref | Ref | 1.57 (1.20-2.07) | 0.001 |
| Pulmonary embolism | I26 | 555 / 239805 | 0.98 (0.79-1.21) | 0.843 | Ref | Ref | 1.04 (0.74-1.47) | 0.820 |
| Arrhythmias | I46-I49 | 776 / 240026 | 0.75 (0.63-0.89) | 0.001 | Ref | Ref | 1.71 (1.23-2.40) | 0.002 |
| Heart failure | I50 | 901 / 240151 | 0.62 (0.52-0.73) | 6.32E-08 | Ref | Ref | 1.66 (1.24-2.21) | 0.001 |
| Cerebrovascular diseases | I60-I69 | 1236 / 240486 | 0.81 (0.70-0.93) | 0.003 | Ref | Ref | 1.38 (1.08-1.77) | 0.010 |
| Stroke | I60-I61, I63-I64 | 986 / 240236 | 0.81 (0.70-0.95) | 0.010 | Ref | Ref | 1.34 (1.02-1.76) | 0.034 |
| Intracerebral haemorrhage | I61 | 276 / 239526 | 0.71 (0.52-0.96) | 0.024 | Ref | Ref | 1.43 (0.90-2.28) | 0.132 |
| Cerebral infarction | I63 | 148 / 239398 | 0.77 (0.52-1.14) | 0.198 | Ref | Ref | 0.48 (0.20-1.13) | 0.093 |
| Atherosclerosis | I70 | 69 / 239319 | 0.94 (0.52-1.71) | 0.838 | Ref | Ref | 1.52 (0.50-4.63) | 0.464 |
| Deep vein thrombosis | I80-I82 | 219 / 239469 | 0.98 (0.70-1.37) | 0.918 | Ref | Ref | 0.70 (0.40-1.21) | 0.203 |
| Diseases of the respiratory system | J00-J99 | 3908 / 243158 | 0.72 (0.66-0.78) | 1.24E-15 | Ref | Ref | 1.78 (1.56-2.03) | 1.35E-17 |
| Influenza and Pneumonia | J09-J18 | 2085 / 241335 | 0.74 (0.67-0.83) | 1.63E-07 | Ref | Ref | 1.78 (1.49-2.13) | 2.72E-10 |
| Chronic obstructive bronchitis | J43-J44, J47 | 1057 / 240307 | 0.57 (0.48-0.68) | 1.66E-10 | Ref | Ref | 1.79 (1.37-2.34) | 2.18E-05 |
| Asthma | J45-J46 | 71 / 239321 | 0.67 (0.34-1.31) | 0.244 | Ref | Ref | 1.21 (0.44-3.33) | 0.708 |
| Diseases of the digestive system | K00-K93 | 1316 / 240566 | 0.72 (0.62-0.83) | 9.96E-06 | Ref | Ref | 1.76 (1.44-2.16) | 5.05E-08 |
| Inflammatory bowel disease | K50-K52 | 67 / 239317 | 0.77 (0.40-1.48) | 0.429 | Ref | Ref | 1.97 (0.78-5.01) | 0.153 |
| Alcoholic liver disease | K70 | 168 / 239418 | 0.34 (0.19-0.60) | 2.24E-04 | Ref | Ref | 2.04 (1.30-3.20) | 0.002 |
| Diseases of liver | K70-K77 | 485 / 239735 | 0.51 (0.39-0.68) | 2.58E-06 | Ref | Ref | 2.21 (1.65-2.97) | 1.28E-07 |
| Pancreatitis | K85 | 59 / 239309 | 1.14 (0.59-2.19) | 0.705 | Ref | Ref | 1.23 (0.43-3.50) | 0.697 |
| Diseases of the skin | L00-L99 | 84 / 239334 | 0.68 (0.38-1.21) | 0.190 | Ref | Ref | 1.58 (0.68-3.68) | 0.285 |
| Diseases of the musculoskeletal system | M00-M99 | 359 / 239609 | 0.72 (0.55-0.95) | 0.021 | Ref | Ref | 2.57 (1.68-3.95) | 1.56E-05 |
| Rheumatoid arthritis and related disorders | M05-M06, M08, M13, M30-M35, M45 | 199 / 239449 | 0.61 (0.41-0.91) | 0.016 | Ref | Ref | 2.51 (1.50-4.20) | 4.36E-04 |
| Diseases of the genitourinary system | N00-N99 | 1044 / 240294 | 0.69 (0.59-0.82) | 1.07E-05 | Ref | Ref | 2.01 (1.51-2.66) | 1.45E-06 |
| Renal failure | N17-N19 | 796 / 240046 | 0.66 (0.55-0.80) | 2.51E-05 | Ref | Ref | 2.24 (1.61-3.11) | 1.49E-06 |
| Circulatory and respiratory symptoms | R00-R09 | 78 / 239328 | 0.69 (0.39-1.23) | 0.209 | Ref | Ref | 2.00 (0.78-5.13) | 0.149 |

CI: confidence interval; HR: hazard ratio; ICD: international classification of diseases.

**Table S9. Details of the modifiable factors.**

| **Category** | **Trait** | **ID** | **description** |
| --- | --- | --- | --- |
| Alcohol  intake | Alcohol intake, frequency | 1558 | 1 = Never  2 = Occasionally  3 = 1-3 times/month  4 = 1-2 times/week  5 = 3-4 times/week  6 = Daily |
|  | Alcohol comparison, 10 years | 1628 | 1 = Less nowadays  2 = About the same  3 = More nowadays |
|  | Drinking status | 20117 | 2 = Current  1 = Previous  0 = Never |
| Anthropometry | Hand grip strength^#^ | Derived | Mean of left and right |
|  | Waist circumference^#^ | 48 |  |
|  | Hip circumference^#^ | 49 |  |
|  | Waist-hip ratio^#^ | Derived | Ratio of waist and hip circumference |
|  | Weight^#^ | 21002 |  |
|  | Body fat percentage^#^ | 23099 |  |
|  | Whole body fat mass^#^ | 23100 |  |
|  | Whole body fat-free mass^#^ | 23101 |  |
|  | Whole body water mass^#^ | 23102 |  |
|  | Body mass index^#^ | 23104 |  |
|  | Basal metabolic rate^#^ | 23105 |  |
| Blood  biochemistry | Apolipoprotein A^#^ | 30630 |  |
|  | Apolipoprotein B^#^ | 30640 |  |
|  | Urea^#^ | 30670 |  |
|  | eGFR^#^ | 30700 |  |
|  | C-reactive protein^#^ | 30710 |  |
|  | Glucose^#^ | 30740 |  |
|  | High density lipoprotein cholesterol^#^ | 30760 |  |
|  | Low density lipoprotein cholesterol^#^ | 30780 |  |
|  | Lipoprotein A^#^ | 30790 |  |
|  | Triglycerides^#^ | 30870 |  |
|  | Haemoglobin concentration^#^ | 30020 |  |
|  | Alanine aminotransferase^#^ | 30620 |  |
|  | Gamma glutamyltransferase^#^ | 30730 |  |
| Chronobiology | Sleep duration | 1160 |  |
|  | Day nap | 1190 | 3 = Usually  2 = Sometimes  1 = Never/rarely |
|  | Insomnia | 1200 | 3 = Usually  2 = Sometimes  1 = Never/rarely |
|  | Narcolepsy | 1220 | 3 = All of the time  2 = Often  1 = Sometimes  0 = Never/rarely |
| Diet | Cooked vegetable intake | 1289 | Heaped tablespoons of cooked vegetables eat per day. |
|  | Raw vegetable intake | 1299 | Heaped tablespoons of raw vegetables eat per day. |
|  | Fresh fruit intake | 1309 | Pieces of fresh fruit eat per day. |
|  | Dried fruit intake | 1319 | Pieces of dried fruit eat per day. |
|  | Oily fish intake | 1329 | 5 = Once or more daily  4 = 5-6 times a week  3 = 2-4 times a week  2 = Once a week  1 = Less than once a week  0 = Never |
|  | Non-oily fish intake | 1339 | 5 = Once or more daily  4 = 5-6 times a week  3 = 2-4 times a week  2 = Once a week  1 = Less than once a week  0 = Never |
|  | Processed meat intake | 1349 | 5 = Once or more daily  4 = 5-6 times a week  3 = 2-4 times a week  2 = Once a week  1 = Less than once a week  0 = Never |
|  | Poultry intake | 1359 | 5 = Once or more daily  4 = 5-6 times a week  3 = 2-4 times a week  2 = Once a week  1 = Less than once a week  0 = Never |
|  | Beef intake | 1369 | 5 = Once or more daily  4 = 5-6 times a week  3 = 2-4 times a week  2 = Once a week  1 = Less than once a week  0 = Never |
|  | Lamb intake | 1379 | 5 = Once or more daily  4 = 5-6 times a week  3 = 2-4 times a week  2 = Once a week  1 = Less than once a week  0 = Never |
|  | Pork intake | 1389 | 5 = Once or more daily  4 = 5-6 times a week  3 = 2-4 times a week  2 = Once a week  1 = Less than once a week  0 = Never |
|  | Cheese intake | 1408 | 5 = Once or more daily  4 = 5-6 times a week  3 = 2-4 times a week  2 = Once a week  1 = Less than once a week  0 = Never |
|  | Bread intake | 1438 | Slices of bread eat each week. |
|  | Cereal intake | 1458 | Bowls of cereal eat a week. |
|  | Added salt | 1478 | 4 = Always  3 = Usually  2 = Sometimes  1 = Never/rarely |
|  | Tea intake | 1488 | Cups of tea drink each day |
|  | Coffee intake | 1498 | Cups of coffee drink each day |
|  | Water intake | 1528 |  |
|  | Healthy diet level | Derived | A healthy diet is characterized by a higher consumption of fruits, nuts, vegetables, whole grains, fish, and dairy products, and a lower consumption of refined grains, processed meats, unprocessed red meats, and sugar-sweetened beverages. Healthy diet level was thus generated from the cumulative sum of the level of consumption of the components, ranging from 0 to 7. |
|  | Vitamin supplement | 6155 | 1 = Vitamin A  2 = Vitamin B  3= Vitamin C  4 = Vitamin D  5 = Vitamin E  6 = Folic acid  7 = Multivitamins |
|  | Mineral supplement | 6179 | 1= Fish oil  2= Glucosamine  3= Calcium  4 = Zinc  5= Iron  6 = Selenium |
| Early life and sexual health | Breasted as a baby | 1677 | 0 = No  1 = Yes |
|  | Maternal smoking around birth | 1787 | 0 = No  1 = Yes |
|  | Age first sexual intercourse | 2139 |  |
| General health | Bone mineral density^#^ | 78 |  |
|  | Overall health status | 2178 | 4 = Poor  3 = Fair  2 = Good  1 = Excellent |
|  | Peak expiratory flow^#^ | 3064 |  |
|  | Forced vital capacity (FVC)^#^ | 3062 |  |
|  | Number of treatments/medications taken | 137 |  |
|  | Dental problems | 6149 | 1= Mouth ulcers  2 = Painful gums  3 = Bleeding gums  4 = Loose teeth  5 = Toothache  6 = Dentures |
|  | Potassium in urine^#^ | 30520 |  |
| Physical activity | Walking pace | 924 | 1 = Slow pace  2 = Steady average pace  3 = Brisk pace |
|  | METs walking^#^ | 22037 |  |
|  | METs moderate activity^#^ | 22038 |  |
|  | METs vigorous activity^#^ | 22039 |  |
|  | Total METs^#^ | 22040 |  |
| Psychosocial | Mood swings | 1920 | 0 = No  1 = Yes |
|  | Miserableness | 1930 | 0 = No  1 = Yes |
|  | Irritability | 1940 | 0 = No  1 = Yes |
|  | Sensitivity/hurt feelings | 1950 | 0 = No  1 = Yes |
|  | Fed-up feelings | 1960 | 0 = No  1 = Yes |
|  | Nervous feelings | 1970 | 0 = No  1 = Yes |
|  | Worrier/anxious feelings | 1980 | 0 = No  1 = Yes |
|  | Tense | 1990 | 0 = No  1 = Yes |
|  | Worry too long after embarrassment | 2000 | 0 = No  1 = Yes |
|  | Loneliness, isolation | 2020 | 0 = No  1 = Yes |
|  | Guilty feelings | 2030 | 0 = No  1 = Yes |
|  | Risk taking | 2040 | 0 = No  1 = Yes |
|  | Neuroticism score | 20127 |  |
| Smoking | Smoking | 20116 | 0 = Never  1 = Previous  2 = Current |
| Socioeconomic | Standing job | 806 | 4 = Always  3 = Usually  2 = Sometimes  1 = Never/rarely |
|  | Manual job | 816 | 4 = Always  3 = Usually  2 = Sometimes  1 = Never/rarely |
|  | Shift job | 826 | 4 = Always  3 = Usually  2 = Sometimes  1 = Never/rarely |
|  | Education score^#^ | 26414 |  |
|  | Nitrogen dioxide air pollution^#^ | 24003 |  |
|  | Sound pollution^#^ | 24024 |  |
|  | PM10 air pollution^#^ | 24005 |  |

MET: Metabolic Equivalent Task; PM10: particulate matter with diameter less than or equal to 10 micrometers.

^#^: z-mormalized.

**Table S10. Results of the multivariable regression analysis between modifiable factors and biological age gap.**

| **Description** | **Category** | **Beta** | ***p*-value** |
| --- | --- | --- | --- |
| Alcohol intake, frequency | Alcohol | 0.207 | 1.18E-16^**^ |
| Alcohol comparison, 10 years | Alcohol | 0.057 | 2.70E-01 |
| Drinking status | Alcohol | -0.818 | 6.05E-09^**^ |
| Hand grip strength | Anthropometry | -0.806 | 7.45E-45^**^ |
| Waist circumference | Anthropometry | 0.142 | 1.42E-03^*^ |
| Hip circumference | Anthropometry | -0.134 | 1.70E-03^*^ |
| Weight | Anthropometry | -0.254 | 2.62E-08^**^ |
| Waist-hip ratio | Anthropometry | 0.480 | 8.24E-21^**^ |
| Body fat percentage | Anthropometry | 0.425 | 7.90E-17^**^ |
| Whole body fat mass | Anthropometry | 0.094 | 3.05E-02^*^ |
| Whole body fat-free mass | Anthropometry | -1.056 | 7.23E-53^**^ |
| Whole body water mass | Anthropometry | -1.040 | 6.18E-52^**^ |
| Body mass index | Anthropometry | 0.170 | 6.83E-05^**^ |
| Basal metabolic rate | Anthropometry | -0.841 | 1.20E-40^**^ |
| Apolipoprotein A | Blood chemistry | 0.007 | 8.66E-01 |
| Apolipoprotein B | Blood chemistry | 0.221 | 1.69E-08^**^ |
| Urea | Blood chemistry | 0.293 | 2.07E-11^**^ |
| eGFR | Blood chemistry | 0.759 | 1.02E-35^**^ |
| CRP | Blood chemistry | 0.771 | 6.88E-33^**^ |
| Glucose | Blood chemistry | 0.515 | 2.42E-11^**^ |
| HDL | Blood chemistry | -0.125 | 5.11E-03^*^ |
| LDL | Blood chemistry | 0.176 | 7.31E-06^**^ |
| Lipoprotein A | Blood chemistry | 0.065 | 9.59E-02 |
| Triglycerides | Blood chemistry | 0.331 | 2.57E-12^**^ |
| Haemoglobin concentration | Blood chemistry | 0.128 | 1.12E-02^*^ |
| Alanine aminotransferase | Blood chemistry | 0.294 | 1.56E-07^**^ |
| Gamma glutamyltransferase | Blood chemistry | 0.561 | 3.27E-14^**^ |
| Sleep duration | Chronobiology | 0.043 | 2.57E-01 |
| Day nap | Chronobiology | 0.196 | 2.21E-03^*^ |
| Insomnia | Chronobiology | 0.118 | 2.68E-02^*^ |
| Nacrolepsy | Chronobiology | 0.306 | 5.95E-04^*^ |
| Cooked vegetable intake | Diet | -0.022 | 4.08E-01 |
| Raw vegetable intake | Diet | -0.038 | 1.15E-01 |
| Fresh fruit intake | Diet | -0.031 | 2.64E-01 |
| Dried fruit intake | Diet | -0.112 | 2.47E-04^**^ |
| Oily fish intake | Diet | -0.114 | 7.67E-03^*^ |
| Non-oily fish intake | Diet | -0.005 | 9.12E-01 |
| Processed meat intake | Diet | 0.020 | 5.87E-01 |
| Poultry intake | Diet | -0.182 | 2.44E-04^**^ |
| Beef intake | Diet | -0.045 | 3.24E-01 |
| Lamb intake | Diet | 0.014 | 7.97E-01 |
| Pork intake | Diet | -0.054 | 3.00E-01 |
| Cheese intake | Diet | -0.122 | 2.04E-03^*^ |
| Bread intake | Diet | 0.008 | 1.06E-01 |
| Cereal intake | Diet | -0.016 | 2.51E-01 |
| Added salt | Diet | 0.096 | 2.47E-02^*^ |
| Tea intake | Diet | 0.079 | 1.22E-06^**^ |
| Coffee intake | Diet | -0.066 | 1.71E-03^*^ |
| Water intake | Diet | 0.009 | 6.49E-01 |
| Healthy diet level | Diet | -0.060 | 6.29E-02 |
| Vitamin A | Diet | 0.355 | 2.08E-01 |
| Vitamin B | Diet | 0.114 | 5.48E-01 |
| Vitamin C | Diet | 0.139 | 3.05E-01 |
| Vitamin D | Diet | 0.101 | 6.07E-01 |
| Vitamin E | Diet | 0.306 | 1.83E-01 |
| Folic acid | Diet | 0.171 | 5.00E-01 |
| Multivitamins | Diet | 0.116 | 2.05E-01 |
| Fish oil | Diet | 0.157 | 5.68E-02 |
| Glucosamine | Diet | -0.164 | 9.50E-02 |
| Calcium | Diet | 0.010 | 9.49E-01 |
| Zinc | Diet | 0.047 | 8.06E-01 |
| Iron | Diet | 0.265 | 2.03E-01 |
| Selenium | Diet | 0.161 | 5.18E-01 |
| Breasted as a baby | Early life and sexual health | -0.194 | 2.28E-02^*^ |
| Maternal smoking around birth | Early life and sexual health | 0.112 | 1.76E-01 |
| Age first sexual intercourse | Early life and sexual health | -0.010 | 4.17E-01 |
| Bone mineral density | General health | -0.078 | 8.86E-02 |
| Overall health status | General health | 0.629 | 5.73E-27^**^ |
| Peak expiratory flow | General health | -1.124 | 8.01E-127^**^ |
| Forced vital capacity (FVC) | General health | -1.668 | 1.70E-210^**^ |
| Number of treatments/medications taken | General health | 0.147 | 2.56E-19^**^ |
| Mouth ulcers | General health | -0.001 | 9.94E-01 |
| Painful gums | General health | 0.334 | 1.46E-01 |
| Bleeding gums | General health | 0.075 | 4.99E-01 |
| Loose teeth | General health | 0.561 | 3.04E-03^*^ |
| Toothache | General health | 0.197 | 2.81E-01 |
| Dentures | General health | 0.499 | 2.94E-06^**^ |
| Potassium in urine | General health | -0.325 | 5.36E-16^**^ |
| Walking pace | Physical activity | -0.579 | 6.16E-21^**^ |
| METs walking | Physical activity | 0.091 | 1.55E-02^*^ |
| METs moderate activity | Physical activity | 0.068 | 1.14E-01 |
| METs vigorous activity | Physical activity | 0.142 | 9.04E-03^*^ |
| Total METs | Physical activity | 0.087 | 5.98E-02 |
| Mood swings | Psychological | 0.189 | 1.29E-02^*^ |
| Miserableness | Psychological | 0.101 | 1.92E-01 |
| Irritability | Psychological | 0.119 | 1.59E-01 |
| Sensitivity/hurt feelings | Psychological | 0.181 | 1.85E-02^*^ |
| Fed-up feelings | Psychological | 0.263 | 6.84E-04^*^ |
| Nervous feelings | Psychological | 0.473 | 1.28E-07^**^ |
| Worrier/anxious feelings | Psychological | 0.233 | 2.41E-03^*^ |
| Tense/’highly strung’ | Psychological | 0.298 | 2.52E-03^*^ |
| Worry too long after embarrassment | Psychological | 0.074 | 3.29E-01 |
| Loneliness, isolation | Psychological | 0.221 | 2.30E-02^*^ |
| Guilty feelings | Psychological | 0.104 | 2.15E-01 |
| Risk taking | Psychological | -0.190 | 9.38E-03^*^ |
| Neuroticism score | Psychological | 0.032 | 6.13E-03^*^ |
| Smoking | Smoking | 1.301 | 9.86E-26^**^ |
| Standing job | Socioeconomic | 0.160 | 2.70E-06^**^ |
| Manual job | Socioeconomic | 0.219 | 3.67E-07^**^ |
| Shift job | Socioeconomic | 0.104 | 1.70E-03^*^ |
| Education score | Socioeconomic | 0.136 | 1.61E-03^*^ |
| Nitrogen oxides air pollution | Socioeconomic | -0.198 | 2.91E-06^**^ |
| PM10 air pollution | Socioeconomic | -0.211 | 7.46E-07^**^ |
| Sound pollution | Socioeconomic | -0.107 | 1.00E-02^*^ |

^*^: *p*-value < 0.05;

^**^: *p*-value < 4.81×10^-4^ (0.05/104).

**Table S11. Results of the multivariable regression analysis between modifiable factors and biological age gap in healthy male participants.**

| **Description** | **Category** | **Beta** | ***p*-value** |
| --- | --- | --- | --- |
| Alcohol intake, frequency | Alcohol | -0.155 | 5.14E-05^**^ |
| Drinking status | Alcohol | -0.070 | 5.29E-01 |
| Alcohol comparison, 10 years | Alcohol | -0.602 | 8.95E-03^*^ |
| Body fat-free mass | Anthropometry | -0.819 | 1.32E-28^**^ |
| Body water mass | Anthropometry | -0.074 | 3.02E-01 |
| Hand grip strength | Anthropometry | -0.512 | 2.06E-11^**^ |
| Basal metabolic rate | Anthropometry | -0.537 | 2.23E-15^**^ |
| Waist-hip circumstance | Anthropometry | 0.393 | 1.71E-06^**^ |
| Body fat percentage | Anthropometry | 0.464 | 9.07E-08^**^ |
| Weight | Anthropometry | -0.015 | 8.39E-01 |
| Body mass index | Anthropometry | -1.248 | 2.03E-46^**^ |
| Waist circumstance | Anthropometry | -1.218 | 5.34E-45^**^ |
| Hip circumstance | Anthropometry | -0.009 | 9.01E-01 |
| Whole body fat mass | Anthropometry | -1.063 | 8.80E-41^**^ |
| eGFR | Blood chemistry | -0.034 | 6.30E-01 |
| CRP | Blood chemistry | -0.282 | 1.01E-06^**^ |
| GGT | Blood chemistry | 0.192 | 1.90E-03^*^ |
| TG | Blood chemistry | 0.727 | 1.27E-18^**^ |
| Urea | Blood chemistry | 0.601 | 1.80E-08^**^ |
| Glucose | Blood chemistry | 0.531 | 3.93E-07^**^ |
| ApoB | Blood chemistry | -0.137 | 7.01E-02 |
| ALT | Blood chemistry | -0.373 | 1.77E-10^**^ |
| LDL | Blood chemistry | -0.030 | 5.98E-01 |
| HDL | Blood chemistry | -0.074 | 2.09E-01 |
| Hb | Blood chemistry | 0.001 | 9.90E-01 |
| Lp(a) | Blood chemistry | -0.162 | 2.27E-02^*^ |
| ApoA | Blood chemistry | 0.291 | 1.19E-03^*^ |
| Nacrolepsy | Chronobiology | 0.081 | 2.00E-01 |
| Day nap | Chronobiology | 0.231 | 9.52E-03^*^ |
| Insomnia | Chronobiology | 0.060 | 4.30E-01 |
| Sleep duration | Chronobiology | 0.211 | 5.17E-02 |
| Cooked vegetable intake | Diet | -0.060 | 1.17E-01 |
| Raw vegetable intake | Diet | -0.003 | 9.33E-01 |
| Fresh fruit intake | Diet | -0.031 | 4.47E-01 |
| Dried fruit intake | Diet | -0.145 | 2.08E-03^*^ |
| Oily fish intake | Diet | -0.079 | 2.06E-01 |
| Non-oily fish intake | Diet | 0.002 | 9.80E-01 |
| Processed meat intake | Diet | 0.042 | 4.41E-01 |
| Poultry intake | Diet | -0.243 | 1.11E-03^*^ |
| Beef intake | Diet | -0.006 | 9.35E-01 |
| Lamb intake | Diet | -0.027 | 7.35E-01 |
| Pork intake | Diet | -0.068 | 3.72E-01 |
| Cheese intake | Diet | -0.188 | 1.58E-03^*^ |
| Bread intake | Diet | -0.003 | 6.58E-01 |
| Cereal intake | Diet | -0.027 | 1.86E-01 |
| Tea intake | Diet | 0.251 | 5.51E-05^**^ |
| Coffee intake | Diet | 0.090 | 1.46E-04^**^ |
| Added salt | Diet | -0.076 | 1.17E-02^*^ |
| Fish oil | Diet | 0.000 | 9.91E-01 |
| Healthy diet level | Diet | -0.065 | 1.60E-01 |
| Glucosamine | Diet | 0.475 | 2.52E-01 |
| Vitamin E | Diet | 0.042 | 8.97E-01 |
| Iron | Diet | 0.291 | 1.56E-01 |
| Multivitamins | Diet | 0.268 | 4.64E-01 |
| Vitamin A | Diet | 0.531 | 1.63E-01 |
| Vitamin C | Diet | 0.339 | 4.77E-01 |
| Folic acid | Diet | 0.002 | 9.89E-01 |
| Selenium | Diet | 0.155 | 2.07E-01 |
| Vitamin B | Diet | -0.293 | 5.72E-02 |
| Vitamin D | Diet | 0.227 | 4.91E-01 |
| Water intake | Diet | 0.139 | 6.53E-01 |
| Zinc | Diet | 0.330 | 3.73E-01 |
| Calcium | Diet | -0.139 | 7.12E-01 |
| Breasted as a baby | Early life and sexual health | -0.112 | 3.88E-01 |
| Maternal smoking around birth | Early life and sexual health | -0.044 | 7.16E-01 |
| Age first sexual intercourse | Early life and sexual health | -0.025 | 1.19E-01 |
| FVC | General health | -0.066 | 2.99E-01 |
| PEF | General health | -0.887 | 1.21E-12^**^ |
| Overall health status | General health | -0.948 | 4.79E-59^**^ |
| Number of treatments/medications taken | General health | -1.519 | 5.64E-108^**^ |
| Potassium in urine | General health | 0.195 | 3.09E-15^**^ |
| Dentures | General health | 0.101 | 6.01E-01 |
| Loose teeth | General health | 0.778 | 4.64E-02^*^ |
| Bone mineral density | General health | 0.178 | 3.20E-01 |
| Painful gums | General health | 0.580 | 3.56E-02^*^ |
| Toothache | General health | 0.511 | 5.50E-02 |
| Bleeding gums | General health | 0.433 | 4.63E-03^*^ |
| Mouth ulcers | General health | -0.290 | 6.77E-07^**^ |
| Walking pace | Physical activity | -0.641 | 1.92E-08^**^ |
| METs vigorous activity | Physical activity | 0.067 | 2.20E-01 |
| METs walking | Physical activity | 0.054 | 3.82E-01 |
| Total METs | Physical activity | 0.142 | 2.83E-02^*^ |
| METs moderate activity | Physical activity | 0.097 | 1.22E-01 |
| Nervous feelings | Psychological | 0.361 | 1.31E-03^*^ |
| Fed-up feelings | Psychological | 0.093 | 4.25E-01 |
| Worrier/anxious feelings | Psychological | 0.188 | 1.21E-01 |
| Tense/’highly strung’ | Psychological | 0.084 | 4.51E-01 |
| Neuroticism score | Psychological | 0.266 | 2.13E-02^*^ |
| Risk taking | Psychological | 0.442 | 1.77E-03^*^ |
| Mood swings | Psychological | 0.304 | 6.11E-03^*^ |
| Sensitivity/hurt feelings | Psychological | 0.321 | 3.60E-02^*^ |
| Loneliness, isolation | Psychological | 0.034 | 7.66E-01 |
| Irritability | Psychological | 0.279 | 6.72E-02 |
| Miserableness | Psychological | 0.264 | 4.78E-02^*^ |
| Guilty feelings | Psychological | -0.126 | 2.08E-01 |
| Worry | Psychological | 0.025 | 1.46E-01 |
| Smoking | Smoking | 1.071 | 4.13E-10^**^ |
| Manual job | Socioeconomic | 0.364 | 1.80E-03^*^ |
| PM10 air pollution | Socioeconomic | 0.643 | 1.10E-05^**^ |
| Standing job | Socioeconomic | 0.244 | 1.63E-01 |
| Nitrogen oxides air pollution | Socioeconomic | 0.102 | 9.63E-02 |
| Education score | Socioeconomic | -0.206 | 7.61E-04^*^ |
| Shift job | Socioeconomic | -0.180 | 4.35E-03^*^ |
| Sound pollution | Socioeconomic | -0.104 | 8.47E-02 |

^*^: *p*-value < 0.05

^**^: *p*-value < 4.81×10^-4^ (0.05/104).

**Table S12. Results of the multivariable regression analysis between modifiable factors and biological age gap in healthy female participants.**

| **Description** | **Category** | **Beta** | ***p*-value** |
| --- | --- | --- | --- |
| Alcohol intake, frequency | Alcohol | -0.237 | 5.89E-13^**^ |
| Drinking status | Alcohol | -0.150 | 1.50E-01 |
| Alcohol comparison, 10 years | Alcohol | -0.922 | 1.93E-07^**^ |
| Body fat-free mass | Anthropometry | -0.903 | 1.44E-19^**^ |
| Body water mass | Anthropometry | 0.286 | 9.36E-07^**^ |
| Hand grip strength | Anthropometry | 0.042 | 4.06E-01 |
| Basal metabolic rate | Anthropometry | -0.025 | 7.03E-01 |
| Waist-hip circumstance | Anthropometry | 0.571 | 1.14E-16^**^ |
| Body fat percentage | Anthropometry | 0.416 | 2.00E-10^**^ |
| Weight | Anthropometry | 0.162 | 2.64E-03^*^ |
| Body mass index | Anthropometry | -0.891 | 6.14E-12^**^ |
| Waist circumstance | Anthropometry | -0.909 | 2.23E-12^**^ |
| Hip circumstance | Anthropometry | 0.264 | 5.92E-07^**^ |
| Whole body fat mass | Anthropometry | -0.591 | 5.14E-07^**^ |
| eGFR | Blood chemistry | 0.015 | 7.85E-01 |
| CRP | Blood chemistry | 0.633 | 1.55E-31^**^ |
| GGT | Blood chemistry | 0.375 | 1.18E-09^**^ |
| TG | Blood chemistry | 0.891 | 1.19E-20^**^ |
| Urea | Blood chemistry | 0.870 | 2.56E-26^**^ |
| Glucose | Blood chemistry | 0.457 | 5.20E-05^**^ |
| ApoB | Blood chemistry | -0.118 | 3.04E-02^*^ |
| ALT | Blood chemistry | 0.607 | 1.22E-29^**^ |
| LDL | Blood chemistry | 0.149 | 4.61E-03^*^ |
| HDL | Blood chemistry | 0.871 | 2.03E-30^**^ |
| Hb | Blood chemistry | 0.219 | 2.72E-03^*^ |
| Lp(a) | Blood chemistry | 0.897 | 7.15E-23^**^ |
| ApoA | Blood chemistry | 1.017 | 1.94E-16^**^ |
| Nacrolepsy | Chronobiology | 0.079 | 1.27E-01 |
| Day nap | Chronobiology | 0.197 | 3.25E-02^*^ |
| Insomnia | Chronobiology | 0.156 | 3.45E-02^*^ |
| Sleep duration | Chronobiology | 0.285 | 2.16E-02^*^ |
| Cooked vegetable intake | Diet | 0.019 | 5.99E-01 |
| Raw vegetable intake | Diet | -0.066 | 4.08E-02^*^ |
| Fresh fruit intake | Diet | -0.043 | 2.62E-01 |
| Dried fruit intake | Diet | -0.093 | 2.09E-02^*^ |
| Oily fish intake | Diet | -0.146 | 1.22E-02^*^ |
| Non-oily fish intake | Diet | -0.017 | 7.95E-01 |
| Processed meat intake | Diet | 0.001 | 9.82E-01 |
| Poultry intake | Diet | -0.122 | 3.30E-02^*^ |
| Beef intake | Diet | -0.073 | 2.22E-01 |
| Lamb intake | Diet | 0.019 | 8.12E-01 |
| Pork intake | Diet | -0.062 | 4.25E-01 |
| Cheese intake | Diet | -0.069 | 1.95E-01 |
| Bread intake | Diet | 0.027 | 1.66E-03^*^ |
| Cereal intake | Diet | -0.012 | 5.45E-01 |
| Tea intake | Diet | -0.054 | 3.80E-01 |
| Coffee intake | Diet | 0.070 | 1.56E-03^*^ |
| Added salt | Diet | -0.061 | 3.66E-02^*^ |
| Fish oil | Diet | 0.014 | 5.86E-01 |
| Healthy diet level | Diet | -0.059 | 1.90E-01 |
| Glucosamine | Diet | 0.292 | 4.49E-01 |
| Vitamin E | Diet | 0.163 | 4.81E-01 |
| Iron | Diet | 0.014 | 9.38E-01 |
| Multivitamins | Diet | 0.003 | 9.90E-01 |
| Vitamin A | Diet | 0.167 | 5.60E-01 |
| Vitamin C | Diet | 0.140 | 6.39E-01 |
| Folic acid | Diet | 0.203 | 9.06E-02 |
| Selenium | Diet | 0.145 | 1.94E-01 |
| Vitamin B | Diet | -0.114 | 3.70E-01 |
| Vitamin D | Diet | -0.106 | 5.32E-01 |
| Water intake | Diet | 0.001 | 9.95E-01 |
| Zinc | Diet | 0.302 | 2.30E-01 |
| Calcium | Diet | 0.393 | 2.32E-01 |
| Breasted as a baby | Early life and sexual health | -0.272 | 1.64E-02^*^ |
| Maternal smoking around birth | Early life and sexual health | 0.250 | 2.67E-02^*^ |
| Age first sexual intercourse | Early life and sexual health | 0.005 | 7.71E-01 |
| FVC | General health | -0.073 | 2.66E-01 |
| PEF | General health | -0.822 | 1.66E-11^**^ |
| Overall health status | General health | -1.507 | 2.42E-79^**^ |
| Number of treatments/medications taken | General health | -2.057 | 1.36E-114^**^ |
| Potassium in urine | General health | 0.113 | 8.60E-07^**^ |
| Dentures | General health | -0.101 | 5.20E-01 |
| Loose teeth | General health | 0.086 | 7.61E-01 |
| Bone mineral density | General health | 0.005 | 9.69E-01 |
| Painful gums | General health | 0.556 | 3.24E-02^*^ |
| Toothache | General health | -0.086 | 7.30E-01 |
| Bleeding gums | General health | 0.565 | 1.44E-04^**^ |
| Mouth ulcers | General health | -0.357 | 1.70E-10^**^ |
| Walking pace | Physical activity | -0.689 | 6.34E-11^**^ |
| METs vigorous activity | Physical activity | 0.107 | 3.96E-02^*^ |
| METs walking | Physical activity | 0.077 | 2.00E-01 |
| Total METs | Physical activity | 0.128 | 1.40E-01 |
| METs moderate activity | Physical activity | 0.070 | 3.00E-01 |
| Nervous feelings | Psychological | 0.055 | 5.92E-01 |
| Fed-up feelings | Psychological | 0.117 | 2.58E-01 |
| Worrier/anxious feelings | Psychological | 0.074 | 5.28E-01 |
| Tense/’highly strung’ | Psychological | 0.274 | 1.01E-02^*^ |
| Neuroticism score | Psychological | 0.255 | 1.41E-02^*^ |
| Risk taking | Psychological | 0.482 | 2.99E-05^**^ |
| Mood swings | Psychological | 0.152 | 1.53E-01 |
| Sensitivity/hurt feelings | Psychological | 0.271 | 3.61E-02^*^ |
| Loneliness, isolation | Psychological | 0.103 | 3.16E-01 |
| Irritability | Psychological | 0.175 | 1.63E-01 |
| Miserableness | Psychological | -0.011 | 9.19E-01 |
| Guilty feelings | Psychological | -0.273 | 1.15E-02^*^ |
| Worry | Psychological | 0.038 | 1.67E-02^*^ |
| Smoking | Smoking | 1.537 | 1.20E-17^**^ |
| Manual job | Socioeconomic | 0.194 | 7.27E-02 |
| PM10 air pollution | Socioeconomic | 0.498 | 3.40E-03^*^ |
| Standing job | Socioeconomic | 0.367 | 3.95E-02^*^ |
| Nitrogen oxides air pollution | Socioeconomic | 0.159 | 7.66E-03^*^ |
| Education score | Socioeconomic | -0.200 | 6.01E-04^*^ |
| Shift job | Socioeconomic | -0.242 | 2.65E-05^**^ |
| Sound pollution | Socioeconomic | -0.118 | 4.09E-02^*^ |

^*^: *p*-value < 0.05

^**^: *p*-value < 4.81×10^-4^ (0.05/104)

**Table S13. Genomic risk loci for biological age gap.**

| **SNP** | **CHR** | **BP** | **A1** | **A2** | **MAF** | **Beta** | **SE** | **P** |
| --- | --- | --- | --- | --- | --- | --- | --- | --- |
| rs115676662 | 5 | 170005452 | A | G | 0.015 | -0.876 | 0.179 | 1.04E-06 |
| rs537129925 | 7 | 5482336 | C | T | 0.008 | -1.646 | 0.309 | 1.06E-07 |
| rs12538964 | 7 | 154158573 | C | T | 0.13 | 0.485 | 0.098 | 7.42E-07 |
| rs113374973 | 9 | 111197612 | G | C | 0.017 | -1.001 | 0.196 | 3.31E-07 |
| rs139188100 | 10 | 8859989 | T | C | 0.009 | -1.425 | 0.271 | 1.52E-07 |
| 12:123693956_GT_G | 12 | 123693956 | G | GT | 0.047 | 0.874 | 0.169 | 2.34E-07 |
| rs145212059 | 17 | 39272347 | G | T | 0.008 | -1.566 | 0.298 | 1.45E-07 |
| rs141123456 | 18 | 8893166 | TA | T | 0.021 | -1.123 | 0.229 | 9.49E-07 |
| rs3761280 | 20 | 23620076 | G | A | 0.225 | -0.630 | 0.075 | 3.17E-17 |

A1: effect allele; A2: other allele; BP: base pair location; CHR: chromosome; MAF: minor allele frequency; SE: standard error; SNP: single nucleotide polymorphism.

**Table S14. The common variants associated with biological age gap after FDR corrections.**

| **SNP** | **CHR** | **BP** | **A1** | **A2** | **MAF** | **Beta** | **SE** | **P** | **FDR** |
| --- | --- | --- | --- | --- | --- | --- | --- | --- | --- |
| chr20:23602755:G:A | 20 | 23602755 | A | G | 0.23 | -0.556 | 0.071 | 4.72E-15 | 1.26E-10 |
| chr20:23603088:T:C | 20 | 23603088 | C | T | 0.22 | -0.542 | 0.071 | 3.55E-14 | 7.09E-10 |
| chr20:23637790:C:T | 20 | 23637790 | T | C | 0.22 | -0.594 | 0.072 | 1.83E-16 | 1.46E-11 |
| chr20:23637934:T:G | 20 | 23637934 | G | T | 0.22 | -0.587 | 0.072 | 4.61E-16 | 1.84E-11 |
| chr20:23685847:T:G | 20 | 23685847 | G | T | 0.12 | -0.461 | 0.092 | 5.91E-07 | 0.009 |

A1: effect allele; A2: other allele; BP: base pair location; CHR: chromosome; FDR: false-discovery rate; MAF: minor allele frequency; SE: standard error; SNP: single nucleotide polymorphism.

**Table S15. GWAS summary-level data used in linkage disequilibrium score correlation analysis.**

| **Disease** | **Finngen ID** | **Case** | **Control** |
| --- | --- | --- | --- |
| Prostate_cancer | finngen_R8_C3_PROSTATE_EXALLC | 11,590 | 110,189 |
| Anaemias | finngen_R8_D3_ANAEMIA | 23,830 | 81,923 |
| Diabetes | finngen_R8_T2D_WIDE | 33,043 | 284,971 |
| Obesity | finngen_R8_E4_OBESITY | 18,330 | 324,070 |
| Any mental disorder | finngen_R8_KRA_PSY_ANYMENTAL | 87,523 | 254,976 |
| Dementia | finngen_R8_F5_DEMENTIA | 13,517 | 325,306 |
| Substance abuse (more control exclusions) | finngen_R8_KRA_PSY_SUBSTANCE_EXMORE | 21,032 | 254,921 |
| Acute and transient psychotic disorders | finngen_R8_F5_PSYTRANS | 4,025 | 330,132 |
| Epilepsy | finngen_R8_G6_EPLEPSY | 10,354 | 264,662 |
| Transient ischemic attack | finngen_R8_I9_TIA | 16,115 | 311,743 |
| Sleep disorders (combined) | finngen_R8_SLEEP | 37,972 | 301,213 |
| Hypertension | finngen_R8_I9_HYPTENS | 98,683 | 243,756 |
| Ischaemic heart disease | finngen_R8_I9_IHD | 56,730 | 285,769 |
| Angina pectoris | finngen_R8_I9_ANGINA | 30,670 | 285,621 |
| Myocardial infarction | finngen_R8_I9_MI_STRICT | 21,609 | 285,621 |
| Pulmonary embolism | finngen_R8_I9_PULMEMB | 8,170 | 333,487 |
| Cardiac arrhythmias | finngen_R8_CARDIAC_ARRHYTM | 59,182 | 204,429 |
| All-cause Heart Failure | finngen_R8_I9_HEARTFAIL_ALLCAUSE | 23,622 | 317,939 |
| Cerebrovascular diseases | finngen_R8_FG_CEREBVASC | 25,899 | 168,000 |
| Stroke | finngen_R8_I9_STR | 22,329 | 309,698 |
| Intracerebral haemmorrhage | finngen_R8_I9_ICH | 3,232 | 309,693 |
| Atherosclerosis, excluding cerebral, coronary and PAD | finngen_R8_I9_ATHSCLE | 13,434 | 317,899 |
| Influenza and Pneumonia | finngen_R8_J10_INFLUPNEU | 55,880 | 286,619 |
| Chronic obstructive bronchitis | finngen_R8_J10_COPD | 16,410 | 283,589 |
| Asthma | finngen_R8_J10_ASTHMA_EXMORE | 37,253 | 187,112 |
| Inflammatory bowel disease | finngen_R8_K11_IBD_STRICT_PSC | 6,440 | 335,734 |
| Alcoholic liver disease | finngen_R8_K11_ALCOLIV | 2,513 | 332,951 |
| Diseases of liver | finngen_R8_K11_LIVER | 9,548 | 332,951 |
| Gout | finngen_R8_M13_GOUT | 7,461 | 221,323 |
| Sciatica | finngen_R8_M13_SCIATICA | 15,980 | 248,831 |
| Renal failure | finngen_R8_N14_RENFAIL | 12,199 | 330,300 |

**Table S16. Results of linkage disequilibrium score correlation analysis of biological age gap with health-related outcomes.**

| **Outcome** | **Category** | **Rg** | **SE** | **Z** | **P** |
| --- | --- | --- | --- | --- | --- |
| Prostate cancer | Cancer | -0.154 | 0.117 | -1.314 | 0.189 |
| Anaemia | Blood | 0.069 | 0.123 | 0.564 | 0.573 |
| Obesity | Endocrine | 0.158 | 0.075 | 2.098 | 0.036 |
| Diabetes | Endocrine | 0.131 | 0.079 | 1.660 | 0.097 |
| Any mental disorder | Mental and behavioral | 0.053 | 0.074 | 0.719 | 0.472 |
| Dementia | Mental and behavioral | -0.052 | 0.192 | -0.270 | 0.787 |
| Disorders due to substance abuse | Mental and behavioral | 0.026 | 0.081 | 0.326 | 0.745 |
| Psychotic disorders | Mental and behavioral | 0.077 | 0.171 | 0.450 | 0.653 |
| Epilepsy | Nervous | 0.001 | 0.167 | 0.008 | 0.994 |
| Transient ischemic attack | Nervous | 0.209 | 0.147 | 1.421 | 0.156 |
| Sleep disorders | Nervous | 0.050 | 0.096 | 0.522 | 0.601 |
| Hypertension | Circulatory | 0.205 | 0.075 | 2.733 | 0.006 |
| Ischaemic heart disease | Circulatory | 0.126 | 0.075 | 1.678 | 0.093 |
| Angina pectoris | Circulatory | 0.020 | 0.080 | 0.253 | 0.800 |
| Myocardial infarction | Circulatory | 0.204 | 0.097 | 2.092 | 0.037 |
| Pulmonary embolism | Circulatory | 0.364 | 0.173 | 2.108 | 0.035 |
| Arrhythmias | Circulatory | 0.031 | 0.080 | 0.387 | 0.699 |
| Heart failure | Circulatory | 0.211 | 0.124 | 1.697 | 0.090 |
| Cerebrovascular diseases | Circulatory | 0.191 | 0.106 | 1.810 | 0.070 |
| Stroke | Circulatory | 0.216 | 0.143 | 1.508 | 0.132 |
| Intracerebral haemmorrhage | Circulatory | -0.358 | 0.203 | -1.763 | 0.078 |
| Atherosclerosis | Circulatory | 0.247 | 0.122 | 2.021 | 0.043 |
| Influenza and Pneumonia | Respiratory | 0.278 | 0.125 | 2.226 | 0.026 |
| Chronic obstructive bronchitis | Respiratory | 0.284 | 0.099 | 2.868 | 0.004 |
| Asthma | Respiratory | 0.129 | 0.091 | 1.420 | 0.156 |
| Inflammatory bowel disease | Digestive | 0.101 | 0.320 | 0.316 | 0.752 |
| Alcoholic liver disease | Digestive | 0.115 | 0.187 | 0.615 | 0.539 |
| Diseases of liver | Digestive | 0.045 | 0.150 | 0.298 | 0.766 |
| Gout | Endocrine | 0.100 | 0.104 | 0.959 | 0.338 |
| Sciatica | Musculoskeletal | 0.137 | 0.097 | 1.416 | 0.157 |
| Renal failure | Genitourinary | 0.427 | 0.178 | 2.391 | 0.017 |

**Table S17. Results of gene mapping analysis.**

| **Methods** | **Genes** | **Number** |
| --- | --- | --- |
| Position | B3GNT4, CLIP1, RSRC2, KNTC1, HCAR1, HCAR3, DENR, CCDC62, HIP1R, VPS37B, ABCB9, OGFOD2, ARL6IP4, PITPNM2, MPHOSPH9, C12orf65, CDK2AP1, SBNO1, SETD8, RILPL2, SNRNP35, RILPL1, TMED2, DDX55, EIF2B1, GTF2H3, TCTN2, CCDC92, ZNF664, FAM101A, SOGA2 | 31 |

**Table S18. Results of Gene Ontology and Kyoto Encyclopedia of Genes and Genomes analyses.**

| **ONTOLOGY** | **ID** | **Description** | **pvalue** | **geneID** | **Count** |
| --- | --- | --- | --- | --- | --- |
| BP | GO:0006413 | translational initiation | 0.0005 | DENR/TMED2/EIF2B1 | 3 |
| BP | GO:0003382 | epithelial cell morphogenesis | 0.0008 | RILPL2/RILPL1 | 2 |
| BP | GO:0060271 | cilium assembly | 0.0010 | MPHOSPH9/RILPL2/RILPL1/TCTN2 | 4 |
| BP | GO:0044782 | cilium organization | 0.0013 | MPHOSPH9/RILPL2/RILPL1/TCTN2 | 4 |
| BP | GO:0061951 | establishment of protein localization to plasma membrane | 0.0027 | RILPL2/RILPL1 | 2 |
| BP | GO:0090150 | establishment of protein localization to membrane | 0.0042 | VPS37B/RILPL2/RILPL1 | 3 |
| BP | GO:0072659 | protein localization to plasma membrane | 0.0054 | RILPL2/RILPL1/TMED2 | 3 |
| BP | GO:1990778 | protein localization to cell periphery | 0.0087 | RILPL2/RILPL1/TMED2 | 3 |
| BP | GO:0010639 | negative regulation of organelle organization | 0.0101 | KNTC1/HIP1R/MPHOSPH9 | 3 |
| BP | GO:0010998 | regulation of translational initiation by eIF2 alpha phosphorylation | 0.0127 | TMED2 | 1 |
| BP | GO:0048194 | Golgi vesicle budding | 0.0127 | TMED2 | 1 |
| BP | GO:1903867 | extraembryonic membrane development | 0.0127 | TMED2 | 1 |
| BP | GO:0001731 | formation of translation preinitiation complex | 0.0140 | DENR | 1 |
| BP | GO:0032525 | somite rostral/caudal axis specification | 0.0140 | TMED2 | 1 |
| BP | GO:0036490 | regulation of translation in response to endoplasmic reticulum stress | 0.0140 | TMED2 | 1 |
| BP | GO:0060453 | regulation of gastric acid secretion | 0.0140 | HIP1R | 1 |
| BP | GO:0075522 | IRES-dependent viral translational initiation | 0.0140 | DENR | 1 |
| BP | GO:1901030 | positive regulation of mitochondrial outer membrane permeabilization involved in apoptotic signaling pathway | 0.0140 | HIP1R | 1 |
| BP | GO:0060670 | branching involved in labyrinthine layer morphogenesis | 0.0165 | TMED2 | 1 |
| BP | GO:0070816 | phosphorylation of RNA polymerase II C-terminal domain | 0.0165 | GTF2H3 | 1 |
| BP | GO:0035672 | oligopeptide transmembrane transport | 0.0177 | ABCB9 | 1 |
| BP | GO:0035791 | platelet-derived growth factor receptor-beta signaling pathway | 0.0177 | HIP1R | 1 |
| BP | GO:0043558 | regulation of translational initiation in response to stress | 0.0177 | TMED2 | 1 |
| BP | GO:0090158 | endoplasmic reticulum membrane organization | 0.0177 | TMED2 | 1 |
| BP | GO:0032933 | SREBP signaling pathway | 0.0190 | TMED2 | 1 |
| BP | GO:0006857 | oligopeptide transport | 0.0202 | ABCB9 | 1 |
| BP | GO:0018146 | keratan sulfate biosynthetic process | 0.0202 | B3GNT4 | 1 |
| BP | GO:0048207 | vesicle targeting, rough ER to cis-Golgi | 0.0202 | TMED2 | 1 |
| BP | GO:0048208 | COPII vesicle coating | 0.0202 | TMED2 | 1 |
| BP | GO:0071501 | cellular response to sterol depletion | 0.0202 | TMED2 | 1 |
| BP | GO:0019885 | antigen processing and presentation of endogenous peptide antigen via MHC class I | 0.0215 | ABCB9 | 1 |
| BP | GO:0036342 | post-anal tail morphogenesis | 0.0215 | TMED2 | 1 |
| BP | GO:2000369 | regulation of clathrin-dependent endocytosis | 0.0215 | HIP1R | 1 |
| BP | GO:0001696 | gastric acid secretion | 0.0227 | HIP1R | 1 |
| BP | GO:0006991 | response to sterol depletion | 0.0227 | TMED2 | 1 |
| BP | GO:0007096 | regulation of exit from mitosis | 0.0227 | KNTC1 | 1 |
| BP | GO:0019081 | viral translation | 0.0227 | DENR | 1 |
| BP | GO:0036499 | PERK-mediated unfolded protein response | 0.0227 | TMED2 | 1 |
| BP | GO:0042339 | keratan sulfate metabolic process | 0.0227 | B3GNT4 | 1 |
| BP | GO:0048268 | clathrin coat assembly | 0.0227 | HIP1R | 1 |
| BP | GO:0051382 | kinetochore assembly | 0.0227 | KNTC1 | 1 |
| BP | GO:1902018 | negative regulation of cilium assembly | 0.0227 | MPHOSPH9 | 1 |
| BP | GO:0002483 | antigen processing and presentation of endogenous peptide antigen | 0.0240 | ABCB9 | 1 |
| BP | GO:0034501 | protein localization to kinetochore | 0.0240 | KNTC1 | 1 |
| BP | GO:1903083 | protein localization to condensed chromosome | 0.0240 | KNTC1 | 1 |
| BP | GO:0031334 | positive regulation of protein-containing complex assembly | 0.0252 | CLIP1/HIP1R | 2 |
| BP | GO:0006901 | vesicle coating | 0.0252 | TMED2 | 1 |
| BP | GO:0043555 | regulation of translation in response to stress | 0.0252 | TMED2 | 1 |
| BP | GO:0060716 | labyrinthine layer blood vessel development | 0.0252 | TMED2 | 1 |
| BP | GO:1903902 | positive regulation of viral life cycle | 0.0265 | VPS37B | 1 |
| BP | GO:0032271 | regulation of protein polymerization | 0.0269 | CLIP1/HIP1R | 2 |
| BP | GO:0002064 | epithelial cell development | 0.0274 | RILPL2/RILPL1 | 2 |
| BP | GO:0039702 | viral budding via host ESCRT complex | 0.0277 | VPS37B | 1 |
| BP | GO:0060713 | labyrinthine layer morphogenesis | 0.0277 | TMED2 | 1 |
| BP | GO:0140467 | integrated stress response signaling | 0.0277 | TMED2 | 1 |
| BP | GO:0010640 | regulation of platelet-derived growth factor receptor signaling pathway | 0.0290 | HIP1R | 1 |
| BP | GO:0048199 | vesicle targeting, to, from or within Golgi | 0.0290 | TMED2 | 1 |
| BP | GO:0051383 | kinetochore organization | 0.0290 | KNTC1 | 1 |
| BP | GO:0034315 | regulation of Arp2/3 complex-mediated actin nucleation | 0.0302 | HIP1R | 1 |
| BP | GO:1901028 | regulation of mitochondrial outer membrane permeabilization involved in apoptotic signaling pathway | 0.0302 | HIP1R | 1 |
| BP | GO:0071459 | protein localization to chromosome, centromeric region | 0.0315 | KNTC1 | 1 |
| BP | GO:0019883 | antigen processing and presentation of endogenous antigen | 0.0327 | ABCB9 | 1 |
| BP | GO:0046755 | viral budding | 0.0327 | VPS37B | 1 |
| BP | GO:0050995 | negative regulation of lipid catabolic process | 0.0327 | HCAR1 | 1 |
| BP | GO:0002183 | cytoplasmic translational initiation | 0.0339 | DENR | 1 |
| BP | GO:0035459 | vesicle cargo loading | 0.0339 | TMED2 | 1 |
| BP | GO:0060669 | embryonic placenta morphogenesis | 0.0339 | TMED2 | 1 |
| BP | GO:0001835 | blastocyst hatching | 0.0352 | CCDC62 | 1 |
| BP | GO:0019076 | viral release from host cell | 0.0352 | VPS37B | 1 |
| BP | GO:0035188 | hatching | 0.0352 | CCDC62 | 1 |
| BP | GO:0035890 | exit from host | 0.0352 | VPS37B | 1 |
| BP | GO:0035891 | exit from host cell | 0.0352 | VPS37B | 1 |
| BP | GO:0071684 | organism emergence from protective structure | 0.0352 | CCDC62 | 1 |
| BP | GO:0002474 | antigen processing and presentation of peptide antigen via MHC class I | 0.0364 | ABCB9 | 1 |
| BP | GO:0010458 | exit from mitosis | 0.0376 | KNTC1 | 1 |
| BP | GO:0036258 | multivesicular body assembly | 0.0376 | VPS37B | 1 |
| BP | GO:0034508 | centromere complex assembly | 0.0389 | KNTC1 | 1 |
| BP | GO:0036257 | multivesicular body organization | 0.0389 | VPS37B | 1 |
| BP | GO:0060674 | placenta blood vessel development | 0.0401 | TMED2 | 1 |
| BP | GO:0090114 | COPII-coated vesicle budding | 0.0401 | TMED2 | 1 |
| BP | GO:0001893 | maternal placenta development | 0.0413 | TMED2 | 1 |
| BP | GO:0031116 | positive regulation of microtubule polymerization | 0.0413 | CLIP1 | 1 |
| BP | GO:0043162 | ubiquitin-dependent protein catabolic process via the multivesicular body sorting pathway | 0.0425 | VPS37B | 1 |
| BP | GO:0051125 | regulation of actin nucleation | 0.0425 | HIP1R | 1 |
| BP | GO:0019068 | virion assembly | 0.0438 | VPS37B | 1 |
| BP | GO:0034260 | negative regulation of GTPase activity | 0.0438 | TMED2 | 1 |
| BP | GO:0097345 | mitochondrial outer membrane permeabilization | 0.0438 | HIP1R | 1 |
| BP | GO:0045742 | positive regulation of epidermal growth factor receptor signaling pathway | 0.0450 | HIP1R | 1 |
| BP | GO:0006623 | protein targeting to vacuole | 0.0462 | VPS37B | 1 |
| BP | GO:0031112 | positive regulation of microtubule polymerization or depolymerization | 0.0462 | CLIP1 | 1 |
| BP | GO:0120033 | negative regulation of plasma membrane bounded cell projection assembly | 0.0462 | MPHOSPH9 | 1 |
| BP | GO:0007094 | mitotic spindle assembly checkpoint signaling | 0.0474 | KNTC1 | 1 |
| BP | GO:0071173 | spindle assembly checkpoint signaling | 0.0474 | KNTC1 | 1 |
| BP | GO:0071174 | mitotic spindle checkpoint signaling | 0.0474 | KNTC1 | 1 |
| BP | GO:1901186 | positive regulation of ERBB signaling pathway | 0.0474 | HIP1R | 1 |
| BP | GO:0051258 | protein polymerization | 0.0483 | CLIP1/HIP1R | 2 |
| BP | GO:0031577 | spindle checkpoint signaling | 0.0486 | KNTC1 | 1 |
| BP | GO:0045841 | negative regulation of mitotic metaphase/anaphase transition | 0.0499 | KNTC1 | 1 |
| BP | GO:0071392 | cellular response to estradiol stimulus | 0.0499 | CCDC62 | 1 |
| CC | GO:0005814 | centriole | 0.0008 | MPHOSPH9/RILPL1/CCDC92 | 3 |
| CC | GO:0097550 | transcription preinitiation complex | 0.0127 | GTF2H3 | 1 |
| CC | GO:0000813 | ESCRT I complex | 0.0139 | VPS37B | 1 |
| CC | GO:0042589 | zymogen granule membrane | 0.0139 | TMED2 | 1 |
| CC | GO:0000776 | kinetochore | 0.0148 | CLIP1/KNTC1 | 2 |
| CC | GO:0000439 | transcription factor TFIIH core complex | 0.0152 | GTF2H3 | 1 |
| CC | GO:0005675 | transcription factor TFIIH holo complex | 0.0165 | GTF2H3 | 1 |
| CC | GO:0036038 | MKS complex | 0.0165 | TCTN2 | 1 |
| CC | GO:0000779 | condensed chromosome, centromeric region | 0.0168 | CLIP1/KNTC1 | 2 |
| CC | GO:0042588 | zymogen granule | 0.0177 | TMED2 | 1 |
| CC | GO:0036064 | ciliary basal body | 0.0178 | RILPL2/RILPL1 | 2 |
| CC | GO:0030662 | coated vesicle membrane | 0.0210 | HIP1R/TMED2 | 2 |
| CC | GO:0001726 | ruffle | 0.0212 | CLIP1/HIP1R | 2 |
| CC | GO:0005828 | kinetochore microtubule | 0.0227 | KNTC1 | 1 |
| CC | GO:0030663 | COPI-coated vesicle membrane | 0.0227 | TMED2 | 1 |
| CC | GO:0032806 | carboxy-terminal domain protein kinase complex | 0.0240 | GTF2H3 | 1 |
| CC | GO:0030137 | COPI-coated vesicle | 0.0277 | TMED2 | 1 |
| CC | GO:0035371 | microtubule plus-end | 0.0290 | CLIP1 | 1 |
| CC | GO:0000775 | chromosome, centromeric region | 0.0337 | CLIP1/KNTC1 | 2 |
| CC | GO:0036452 | ESCRT complex | 0.0351 | VPS37B | 1 |
| CC | GO:0005689 | U12-type spliceosomal complex | 0.0364 | SNRNP35 | 1 |
| CC | GO:0032839 | dendrite cytoplasm | 0.0376 | HIP1R | 1 |
| CC | GO:0005669 | transcription factor TFIID complex | 0.0413 | GTF2H3 | 1 |
| CC | GO:1990752 | microtubule end | 0.0413 | CLIP1 | 1 |
| CC | GO:0000793 | condensed chromosome | 0.0416 | CLIP1/KNTC1 | 2 |
| MF | GO:0003743 | translation initiation factor activity | 0.0023 | DENR/EIF2B1 | 2 |
| MF | GO:0008135 | translation factor activity, RNA binding | 0.0062 | DENR/EIF2B1 | 2 |
| MF | GO:0090079 | translation regulator activity, nucleic acid binding | 0.0101 | DENR/EIF2B1 | 2 |
| MF | GO:0008526 | phosphatidylinositol transfer activity | 0.0140 | PITPNM2 | 1 |
| MF | GO:0035673 | oligopeptide transmembrane transporter activity | 0.0154 | ABCB9 | 1 |
| MF | GO:0008499 | UDP-galactose:beta-N-acetylglucosamine beta-1,3-galactosyltransferase activity | 0.0196 | B3GNT4 | 1 |
| MF | GO:1904680 | peptide transmembrane transporter activity | 0.0210 | ABCB9 | 1 |
| MF | GO:0008525 | phosphatidylcholine transporter activity | 0.0237 | PITPNM2 | 1 |
| MF | GO:0048531 | beta-1,3-galactosyltransferase activity | 0.0237 | B3GNT4 | 1 |
| MF | GO:0035615 | clathrin adaptor activity | 0.0251 | HIP1R | 1 |
| MF | GO:0140312 | cargo adaptor activity | 0.0265 | HIP1R | 1 |
| MF | GO:0031418 | L-ascorbic acid binding | 0.0279 | OGFOD2 | 1 |
| MF | GO:0051010 | microtubule plus-end binding | 0.0293 | CLIP1 | 1 |
| MF | GO:0042288 | MHC class I protein binding | 0.0306 | ABCB9 | 1 |
| MF | GO:0070182 | DNA polymerase binding | 0.0306 | CDK2AP1 | 1 |
| MF | GO:0120014 | phospholipid transfer activity | 0.0334 | PITPNM2 | 1 |
| MF | GO:0080025 | phosphatidylinositol-3,5-bisphosphate binding | 0.0388 | HIP1R | 1 |
| MF | GO:0035250 | UDP-galactosyltransferase activity | 0.0402 | B3GNT4 | 1 |
| MF | GO:0043325 | phosphatidylinositol-3,4-bisphosphate binding | 0.0402 | HIP1R | 1 |
| MF | GO:0031210 | phosphatidylcholine binding | 0.0429 | PITPNM2 | 1 |
| MF | GO:0008378 | galactosyltransferase activity | 0.0443 | B3GNT4 | 1 |
| MF | GO:0042887 | amide transmembrane transporter activity | 0.0483 | ABCB9 | 1 |
| KEGG | hsa04024 | cAMP signaling pathway | 0.0184 | HCAR1/HCAR3 | 2 |
| KEGG | hsa00601 | Glycosphingolipid biosynthesis - lacto and neolacto series | 0.0258 | B3GNT4 | 1 |
| KEGG | hsa02010 | ABC transporters | 0.0426 | ABCB9 | 1 |
| KEGG | hsa03022 | Basal transcription factors | 0.0426 | GTF2H3 | 1 |
| KEGG | hsa03420 | Nucleotide excision repair | 0.0445 | GTF2H3 | 1 |

**Table S19. Results of colocalization analysis of *CST3* tissue expression with biological age gap.**

| **Tissues** | **PPH0** | **PPH1** | **PPH2** | **PPH3** | **PPH4** | **PPH3+PPH4** | **PPH4/PPH3** | **Evidence** |
| --- | --- | --- | --- | --- | --- | --- | --- | --- |
| Adipose Subcutaneous | 2.29E-12 | 5.63E-01 | 1.37E-12 | 0.338 | 0.098 | 0.437 | 0.291 |  |
| Adipose Visceral Omentum | 2.58E-12 | 6.36E-01 | 1.32E-12 | 0.326 | 0.038 | 0.364 | 0.116 |  |
| Adrenal Gland | 2.52E-12 | 6.20E-01 | 1.37E-12 | 0.336 | 0.043 | 0.380 | 0.129 |  |
| Artery Aorta | 2.52E-13 | 6.22E-02 | 6.96E-13 | 0.171 | 0.767 | 0.938 | 4.492 | Suggestive |
| Artery Coronary | 2.43E-12 | 5.97E-01 | 1.47E-12 | 0.360 | 0.043 | 0.403 | 0.120 |  |
| Artery Tibial | 4.21E-13 | 1.04E-01 | 6.94E-13 | 0.170 | 0.726 | 0.896 | 4.261 |  |
| Brain Amygdala | 2.45E-12 | 6.02E-01 | 1.43E-12 | 0.351 | 0.047 | 0.398 | 0.135 |  |
| Brain Anterior cingulate cortex BA24 | 2.44E-12 | 5.98E-01 | 1.45E-12 | 0.357 | 0.045 | 0.402 | 0.127 |  |
| Brain Caudate basal ganglia | 2.37E-12 | 5.81E-01 | 1.51E-12 | 0.370 | 0.049 | 0.419 | 0.132 |  |
| Brain Cerebellar Hemisphere | 2.26E-12 | 5.57E-01 | 1.48E-12 | 0.364 | 0.079 | 0.443 | 0.218 |  |
| Brain Cerebellum | 7.75E-13 | 1.90E-01 | 2.46E-12 | 0.605 | 0.205 | 0.810 | 0.339 |  |
| Brain Cortex | 2.17E-12 | 5.31E-01 | 1.75E-12 | 0.430 | 0.039 | 0.469 | 0.091 |  |
| Brain Frontal Cortex BA9 | 2.42E-12 | 5.95E-01 | 1.48E-12 | 0.362 | 0.043 | 0.405 | 0.117 |  |
| Brain Hippocampus | 2.42E-12 | 5.92E-01 | 1.41E-12 | 0.345 | 0.063 | 0.408 | 0.181 |  |
| Brain Hypothalamus | 2.42E-12 | 5.94E-01 | 1.33E-12 | 0.328 | 0.078 | 0.406 | 0.238 |  |
| Brain Nucleus accumbens basal ganglia | 2.46E-12 | 6.03E-01 | 1.36E-12 | 0.335 | 0.062 | 0.397 | 0.185 |  |
| Brain Putamen basal ganglia | 2.49E-12 | 6.12E-01 | 1.40E-12 | 0.343 | 0.046 | 0.388 | 0.133 |  |
| Brain Spinal cord cervical c-1 | 2.44E-12 | 5.99E-01 | 1.43E-12 | 0.351 | 0.049 | 0.401 | 0.140 |  |
| Brain Substantia nigra | 2.42E-12 | 5.93E-01 | 1.44E-12 | 0.353 | 0.054 | 0.407 | 0.154 |  |
| Breast Mammary Tissue | 2.55E-12 | 6.27E-01 | 1.35E-12 | 0.334 | 0.039 | 0.373 | 0.117 |  |
| Colon Sigmoid | 3.79E-15 | 9.33E-04 | 3.95E-13 | 0.096 | 0.903 | 0.999 | 9.365 | Strong |
| Colon Transverse | 1.97E-12 | 4.84E-01 | 1.20E-12 | 0.294 | 0.222 | 0.516 | 0.757 |  |
| Esophagus Gastroesophageal Junction | 2.58E-12 | 6.35E-01 | 1.31E-12 | 0.323 | 0.043 | 0.365 | 0.132 |  |
| Esophagus Mucosa | 1.05E-13 | 2.59E-02 | 3.97E-13 | 0.097 | 0.877 | 0.974 | 9.051 | Suggestive |
| Esophagus Muscularis | 1.79E-12 | 4.40E-01 | 2.10E-12 | 0.518 | 0.042 | 0.560 | 0.080 |  |
| Heart Atrial Appendage | 6.90E-14 | 1.70E-02 | 5.96E-13 | 0.146 | 0.837 | 0.983 | 5.728 | Suggestive |
| Heart Left Ventricle | 2.28E-12 | 5.63E-01 | 1.61E-12 | 0.396 | 0.041 | 0.437 | 0.104 |  |
| Liver | 2.43E-12 | 5.99E-01 | 1.45E-12 | 0.356 | 0.045 | 0.401 | 0.127 |  |
| Lung | 1.34E-18 | 3.30E-07 | 3.20E-13 | 0.078 | 0.922 | 1.000 | 11.813 | Strong |
| Minor Salivary Gland | 2.47E-12 | 6.10E-01 | 1.38E-12 | 0.341 | 0.049 | 0.390 | 0.145 |  |
| Muscle Skeletal | 1.61E-16 | 3.98E-05 | 2.31E-13 | 0.056 | 0.944 | 1.000 | 16.831 | Strong |
| Nerve Tibial | 2.67E-14 | 6.59E-03 | 6.27E-13 | 0.154 | 0.840 | 0.993 | 5.460 | Strong |
| Ovary | 2.40E-12 | 5.90E-01 | 1.36E-12 | 0.335 | 0.075 | 0.410 | 0.222 |  |
| Pancreas | 2.36E-12 | 5.82E-01 | 1.51E-12 | 0.371 | 0.047 | 0.418 | 0.126 |  |
| Pituitary | 2.45E-12 | 6.03E-01 | 1.35E-12 | 0.333 | 0.064 | 0.397 | 0.193 |  |
| Prostate | 2.46E-12 | 6.07E-01 | 1.36E-12 | 0.334 | 0.058 | 0.393 | 0.174 |  |
| Skin Not Sun Exposed Suprapubic | 1.90E-15 | 4.67E-04 | 3.15E-13 | 0.077 | 0.923 | 1.000 | 12.038 | Strong |
| Skin Sun Exposed Lower leg | 1.86E-12 | 4.58E-01 | 1.06E-12 | 0.260 | 0.282 | 0.542 | 1.083 |  |
| Small Intestine Terminal Ileum | 2.32E-12 | 5.71E-01 | 1.38E-12 | 0.341 | 0.088 | 0.429 | 0.259 |  |
| Spleen | 2.19E-12 | 5.39E-01 | 1.33E-12 | 0.327 | 0.134 | 0.461 | 0.409 |  |
| Stomach | 1.66E-12 | 4.09E-01 | 1.04E-12 | 0.256 | 0.335 | 0.591 | 1.312 |  |
| Testis | 1.58E-21 | 3.90E-10 | 3.95E-13 | 0.096 | 0.904 | 1.000 | 9.380 | Strong |
| Thyroid | 1.92E-12 | 4.73E-01 | 1.37E-12 | 0.338 | 0.189 | 0.527 | 0.559 |  |
| Uterus | 2.45E-12 | 6.04E-01 | 1.39E-12 | 0.343 | 0.053 | 0.396 | 0.155 |  |
| Vagina | 2.36E-12 | 5.82E-01 | 1.37E-12 | 0.337 | 0.082 | 0.418 | 0.242 |  |
| Whole Blood | 1.62E-18 | 3.99E-07 | 3.49E-13 | 0.085 | 0.915 | 1.000 | 10.767 | Strong |

**Table S20. Biological characteristics and rs3761280 genotypes.**

| **Category** | **Trait** | **rs3761280 genotype** | | | **P** |
| --- | --- | --- | --- | --- | --- |
|  |  | **AA** | **AG** | **GG** |  |
| Cardiovascular | Pulse rate | 69.29±11.08 | 69.33±11.09 | 69.36±11.05 | 0.48 |
| Cardiovascular | Diastolic blood pressure, mmHg | 82.2±10.04 | 82.18±10.03 | 82.33±10.08 | 0.18 |
| Cardiovascular | Systolic blood pressure, mmHg | 137.68±18.41 | 137.76±18.45 | 138±18.64 | 0.07 |
| Cardiovascular | Pulse pressure, mmHg | 55.48±13.93 | 55.57±13.98 | 55.66±14.02 | 0.05 |
| Cardiovascular | Mean arterial pressure, mmHg | 100.7±11.71 | 100.71±11.71 | 100.89±11.82 | 0.12 |
| Cognition and mental | Number of correct matches in round | 4.37±0.71 | 4.37±0.71 | 4.36±0.73 | 0.33 |
| Cognition and mental | Number of incorrect matches in round | 2.34±1.88 | 2.35±1.88 | 2.36±1.9 | 0.20 |
| Cognition and mental | Time to complete round, second | 214.89±101.65 | 215.5±102.32 | 215.67±101.92 | 0.17 |
| Cognition and mental | Sleep duration, hour | 7.15±1.1 | 7.15±1.1 | 7.16±1.12 | 0.58 |
| Cognition and mental | Mean time to correctly identify matches, second | 557.58±112.47 | 558.93±113.58 | 559.91±116.11 | <0.001 |
| Cognition and mental | Neuroticism score | 4.11±3.26 | 4.11±3.27 | 4.13±3.28 | 0.62 |
| Genomic | Z-adjusted T/S log | 0.01±0.98 | 0±0.98 | -0.01±0.98 | 0.02 |
| Hepatic | Albumin | 45.22±2.58 | 45.22±2.58 | 45.2±2.57 | 0.59 |
| Hepatic | Alkaline phosphatase | 83.15±23.16 | 83.3±23.28 | 83.56±23.23 | 0.02 |
| Hepatic | Alanine aminotransferase | 23.36±12.45 | 23.38±12.44 | 23.47±12.47 | 0.56 |
| Hepatic | Apolipoprotein A | 1.54±0.27 | 1.54±0.27 | 1.54±0.27 | 0.003 |
| Hepatic | Apolipoprotein B | 1.03±0.24 | 1.03±0.24 | 1.03±0.24 | 0.47 |
| Hepatic | Aspartate aminotransferase | 26.03±8.26 | 26.06±8.21 | 26.06±8.1 | 0.63 |
| Hepatic | Direct bilirubin | 1.81±0.76 | 1.81±0.76 | 1.8±0.75 | 0.63 |
| Hepatic | Gamma glutamyltransferase | 36.49±33.47 | 36.68±33.67 | 36.8±33.61 | 0.15 |
| Hepatic | Total bilirubin | 9.11±4.2 | 9.11±4.19 | 9.1±4.2 | 0.85 |
| Hormonal | SHBG | 51.68±27.28 | 51.6±27.2 | 51.36±27.14 | 0.25 |
| Hormonal | Testosterone | 6.1±5.94 | 6.12±5.94 | 6.17±5.95 | 0.30 |
| Immune | White blood cell count | 6.86±1.74 | 6.86±1.74 | 6.87±1.74 | 0.21 |
| Immune | Red blood cell count | 4.52±0.41 | 4.52±0.41 | 4.52±0.41 | 0.90 |
| Immune | Haemoglobin concentration | 14.18±1.22 | 14.18±1.22 | 14.18±1.24 | 0.75 |
| Immune | Haematocrit percentage | 41.11±3.48 | 41.1±3.48 | 41.08±3.52 | 0.40 |
| Immune | Mean corpuscular volume | 91.13±4.41 | 91.13±4.43 | 91.08±4.41 | 0.32 |
| Immune | Mean corpuscular haemoglobin | 31.44±1.75 | 31.44±1.75 | 31.43±1.75 | 0.54 |
| Immune | Mean corpuscular haemoglobin concentration | 34.5±0.92 | 34.5±0.92 | 34.5±0.93 | 0.94 |
| Immune | Red blood cell distribution width | 13.48±0.9 | 13.49±0.91 | 13.49±0.9 | 0.02 |
| Immune | Platelet count | 252.66±57.85 | 252.72±57.56 | 252.45±58.21 | 0.82 |
| Immune | Platelet crit | 0.23±0.05 | 0.23±0.05 | 0.23±0.05 | 0.71 |
| Immune | Mean platelet volume | 9.33±1.07 | 9.33±1.07 | 9.33±1.08 | 0.99 |
| Immune | Platelet distribution width | 16.49±0.51 | 16.49±0.51 | 16.49±0.51 | 0.77 |
| Immune | Lymphocyte count | 1.94±0.6 | 1.95±0.61 | 1.96±0.61 | 0.01 |
| Immune | Monocyte count | 0.47±0.16 | 0.47±0.16 | 0.47±0.17 | 0.01 |
| Immune | Neutrophill count | 4.22±1.37 | 4.22±1.37 | 4.22±1.37 | 0.94 |
| Immune | Eosinophill count | 0.17±0.12 | 0.17±0.13 | 0.17±0.13 | 0.03 |
| Immune | Basophill count | 0.03±0.04 | 0.03±0.04 | 0.03±0.04 | 0.02 |
| Immune | Nucleated red blood cell count | 0±0.01 | 0±0.01 | 0±0.01 | 0.80 |
| Immune | Reticulocyte count | 0.06±0.02 | 0.06±0.02 | 0.06±0.02 | 0.72 |
| Immune | Mean reticulocyte volume | 105.91±7.56 | 105.94±7.54 | 105.9±7.64 | 0.45 |
| Immune | Mean sphered cell volume | 82.88±5.17 | 82.88±5.16 | 82.88±5.16 | 0.94 |
| Immune | Immature reticulocyte fraction | 0.29±0.06 | 0.29±0.06 | 0.29±0.06 | 0.53 |
| Immune | High light scatter reticulocyte percentage | 0.4±0.2 | 0.4±0.2 | 0.4±0.2 | 0.52 |
| Immune | High light scatter reticulocyte count | 0.02±0.01 | 0.02±0.01 | 0.02±0.01 | 0.74 |
| Immune | C-reactive protein | 2.51±3.71 | 2.52±3.71 | 2.53±3.67 | 0.58 |
| Metabolic | Body fat percentage | 31.32±8.5 | 31.34±8.48 | 31.36±8.55 | 0.72 |
| Metabolic | Whole body fat mass | 24.72±9.41 | 24.72±9.36 | 24.74±9.34 | 0.93 |
| Metabolic | Whole body fat-free mass | 53.35±11.47 | 53.31±11.42 | 53.33±11.36 | 0.59 |
| Metabolic | Whole body water mass | 39.04±8.39 | 39.01±8.36 | 39.02±8.3 | 0.61 |
| Metabolic | Basal metabolic rate | 6628.68±1357.31 | 6623.55±1350.4 | 6626.04±1339.4 | 0.54 |
| Metabolic | Leg fat mass | 4.25±1.83 | 4.24±1.83 | 4.25±1.83 | 0.82 |
| Metabolic | Leg fat-free mass | 8.94±2 | 8.93±1.99 | 8.93±1.97 | 0.50 |
| Metabolic | Arm fat mass | 1.27±0.64 | 1.27±0.64 | 1.27±0.64 | 0.63 |
| Metabolic | Arm fat-free mass | 2.92±0.82 | 2.92±0.82 | 2.92±0.81 | 0.86 |
| Metabolic | Trunk fat mass | 13.7±5.1 | 13.7±5.07 | 13.73±5.06 | 0.74 |
| Metabolic | Trunk fat-free mass | 29.65±5.96 | 29.64±5.94 | 29.65±5.93 | 0.67 |
| Metabolic | Calcium | 2.38±0.09 | 2.38±0.09 | 2.38±0.09 | 0.89 |
| Metabolic | Cholesterol | 5.69±1.13 | 5.69±1.13 | 5.68±1.14 | 0.02 |
| Metabolic | Glucose | 5.11±1.07 | 5.11±1.09 | 5.13±1.12 | 0.06 |
| Metabolic | Glycated haemoglobin | 36.04±6.1 | 36.07±6.16 | 36.14±6.23 | 0.06 |
| Metabolic | HDL cholesterol | 1.45±0.38 | 1.45±0.38 | 1.44±0.38 | 0.01 |
| Metabolic | IGF-1 | 21.44±5.54 | 21.37±5.53 | 21.34±5.52 | <0.001 |
| Metabolic | LDL direct | 3.56±0.86 | 3.55±0.86 | 3.55±0.86 | 0.30 |
| Metabolic | Lipoprotein A | 44.61±49.11 | 44.84±49.31 | 44.68±49.03 | 0.40 |
| Metabolic | Phosphate | 1.16±0.16 | 1.16±0.16 | 1.16±0.16 | 0.59 |
| Metabolic | Total protein | 72.49±4.04 | 72.5±4.04 | 72.52±4.05 | 0.34 |
| Metabolic | Triglycerides | 1.74±0.99 | 1.74±0.99 | 1.75±1 | 0.10 |
| Metabolic | Vitamin D | 48.47±20.77 | 48.46±20.84 | 48.27±20.84 | 0.46 |
| Musculoskeletal | Hand grip strength | 30.76±10.95 | 30.68±10.93 | 30.75±10.97 | 0.09 |
| Musculoskeletal | Waist circumference | 90.25±13.33 | 90.24±13.29 | 90.47±13.15 | 0.08 |
| Musculoskeletal | Hip circumference | 103.33±9.01 | 103.31±8.97 | 103.3±8.93 | 0.71 |
| Musculoskeletal | Seated height | 137.05±7.1 | 137.05±7.07 | 136.93±7.18 | 0.11 |
| Musculoskeletal | Body mass index | 27.39±4.69 | 27.38±4.68 | 27.39±4.64 | 0.78 |
| Musculoskeletal | Weight | 78.04±15.7 | 78±15.61 | 78.05±15.45 | 0.66 |
| Musculoskeletal | Impedance of whole body | 599.28±87.93 | 599.8±87.96 | 600.11±87.85 | 0.14 |
| Musculoskeletal | Impedance of leg | 246.96±34.68 | 247.34±34.74 | 247.56±34.77 | 0.001 |
| Musculoskeletal | Impedance of arm | 324.93±54.3 | 325.08±54.38 | 325.29±54.32 | 0.55 |
| Musculoskeletal | Waist-Hip circumference ratio | 0.87±0.09 | 0.87±0.09 | 0.87±0.09 | <0.001 |
| Pulmonary | Exposure to tobacco smoke at home | 0.53±4.43 | 0.55±4.65 | 0.54±4.46 | 0.26 |
| Pulmonary | Exposure to tobacco smoke outside home | 0.5±2.48 | 0.5±2.55 | 0.51±2.41 | 0.81 |
| Pulmonary | Forced vital capacity (FVC) | 3.6±0.98 | 3.6±0.98 | 3.59±0.99 | 0.07 |
| Pulmonary | Forced expiratory volume in 1-second (FEV1) | 2.73±0.77 | 2.72±0.77 | 2.72±0.78 | 0.29 |
| Pulmonary | Peak expiratory flow | 369.26±127.32 | 369.41±127.26 | 369.35±127.15 | 0.94 |
| Pulmonary | FEV1/FVC | 0.76±0.11 | 0.76±0.11 | 0.76±0.11 | 0.33 |
| Renal | Creatinine in urine | 8873.33±5704.1 | 8865.39±5688.73 | 8852.79±5681.49 | 0.85 |
| Renal | Potassium in urine | 63.11±33.65 | 63.02±33.59 | 62.87±33.56 | 0.53 |
| Renal | Sodium in urine | 77.43±44.01 | 77.32±44.1 | 77.53±44.06 | 0.70 |
| Renal | Urea | 5.39±1.31 | 5.39±1.31 | 5.38±1.3 | 0.60 |
| Renal | Creatinine | 72.07±14.66 | 72.1±14.64 | 71.97±14.51 | 0.55 |
| Renal | Cystatin C | 0.92±0.15 | 0.88±0.15 | 0.83±0.14 | <0.001 |
| Renal | Urate | 309.07±79.37 | 309.54±79.83 | 309.67±79.14 | 0.17 |

**Table S21. Results of linear regression analysis of rs3761280 with the biological traits.**

| **Biological traits** | **β** | **SE** | **P** | **Z-score** |
| --- | --- | --- | --- | --- |
| Impedance of leg | 0.345 | 0.086 | <0.001 | 3.991 |
| Mean time to correctly identify matches | 1.012 | 0.295 | 0.001 | 3.430 |
| Basophil count | 0.000 | 0.000 | 0.005 | 2.801 |
| Monocyte count | 0.001 | 0.000 | 0.010 | 2.585 |
| Red blood cell distribution width | 0.006 | 0.002 | 0.016 | 2.420 |
| Lymphocyte count | 0.004 | 0.002 | 0.018 | 2.357 |
| Alkaline phosphatase | 0.141 | 0.063 | 0.025 | 2.246 |
| Pulse pressure | 0.077 | 0.035 | 0.030 | 2.168 |
| Eosinophil count | 0.001 | 0.000 | 0.068 | 1.825 |
| Waist-Hip circumference ratio | 0.000 | 0.000 | 0.277 | 1.088 |
| HDL cholesterol | -0.002 | 0.001 | 0.029 | -2.181 |
| Cholesterol | -0.007 | 0.003 | 0.026 | -2.222 |
| Z-adjusted T/S log | -0.006 | 0.003 | 0.017 | -2.391 |
| Apolipoprotein A | -0.002 | 0.001 | 0.007 | -2.686 |
| IGF-1 | -0.049 | 0.015 | 0.001 | -3.333 |
| Cystatin C | -0.045 | 0.000 | <0.001 | -121.157 |

HDL: high density lipoprotein; IGF: insulin growth factor; SE: standard error.
